# Examining the Desirable Properties of ZnSnO_*y*_ by Annealing Treatment with a Real-Time Observation of Resistivity

**DOI:** 10.1021/acsomega.4c01857

**Published:** 2024-06-05

**Authors:** Aitkazy Kaisha, David Caffrey, Ardak Ainabayev, Olzat Toktarbaiuly, Margulan Ibraimov, Hongqiang Wang, Nurxat Nuraje, Igor V. Shvets

**Affiliations:** † School of Physics and Centre for Research on Adaptive Nanostructures and Nanodevices (CRANN), Trinity College Dublin, Dublin 2, Ireland; ‡ National Nanotechnology Laboratory of Open Type (NNLOT), Al-Farabi Kazakh National University, Al-Farabi Avenue 71, Almaty 050040, Kazakhstan; § Renewable Energy Laboratory National Laboratory Astana (NLA), Nazarbayev University, Kabanbay Batyr 53, Astana 010000, Kazakhstan; ∥ Physics Department School of Sciences and Humanities, Nazarbayev University, Qabanbay Batyr Avenue 53, Astana 010000, Kazakhstan; ⊥ Faculty of Physics and Technology, 71 Al-Farabi Avenue, Almaty 050040, Kazakhstan; # State Key Laboratory of Solidification Processing, Center for Nano Energy Materials, School of Materials Science and Engineering, Northwestern Polytechnical University, Shaanxi Joint Laboratory of Graphene (NPU), Xi’an 710072, P. R. China; ¶ Department of Chemical & Materials Engineering School of Engineering & Digital Science, Nazarbayev University, Astana 010000, Kazakhstan

## Abstract

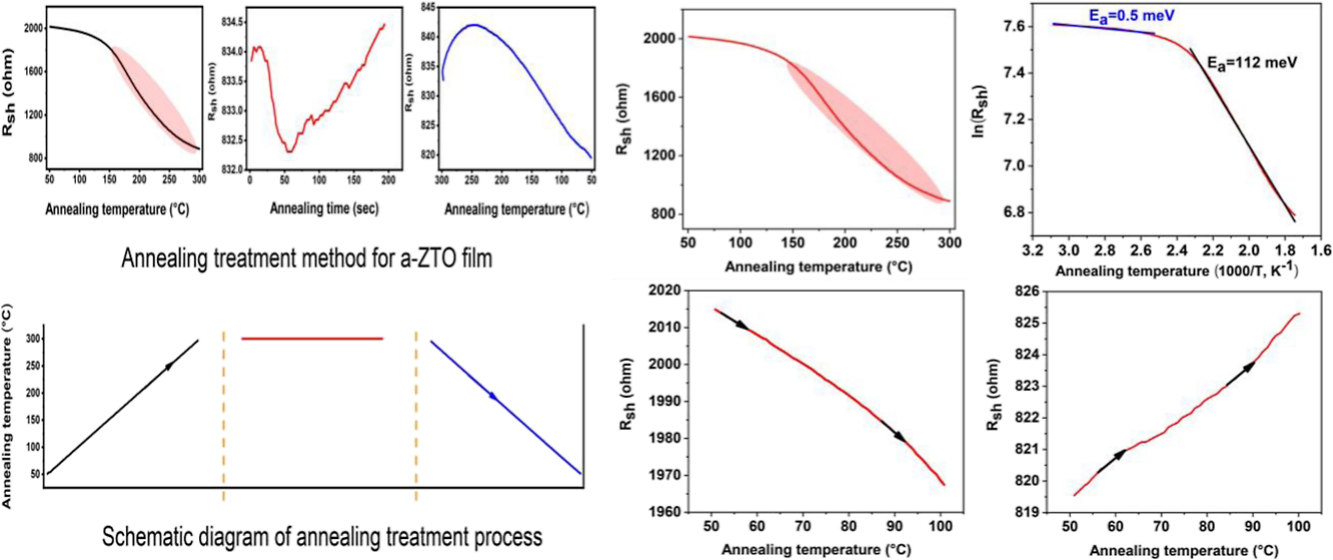

In this report, 38 nm-thick amorphous zinc–tin oxide (a-ZTO) films were deposited by radio frequency magnetron cosputtering. a-ZTO films were annealed by in situ monitoring of the sheet resistance improvements during the annealing process. A sharp drop in the slope of the sheet resistance curve was observed. The activation energies for the sheet resistance slope were calculated. The activation energy of the reaction for a sharp drop in the slope is much higher than the activation energy for the rest of the slope. Based on the activation energy values, six annealing temperatures were selected to saturate the highest conductivity at lower annealing temperatures and to identify the effects associated with annealing time. We found a direct correlation between annealing temperatures and the duration of the annealing treatment. a-ZTO films with a high conductivity of 320 S/cm were achieved by annealing at a temperature of 220 °C. It is noteworthy that the annealing temperature of 220 °C has clearly replaced the temperature of 300 °C. An irreversible decrease in resistivity was observed for all films. The conduction mechanism of films before and after annealing was determined. We confirm that all films individually exhibit semiconducting and metallic behaviors in the conduction mechanism before and after the lowest resistivity saturation.

## 1 Introduction

There is significant interest in transparent conducting oxide (TCO) thin films for optoelectronic devices, including thin film solar cells, OLEDs, flat panel displays, and antifrost window coatings.^1−6^ TCOs are semiconductors with a wide bandgap that simultaneously exhibit high conductivity and transparency in the visible range (*E*_g_ > 3 eV). Recently, there has been increased attention to amorphous oxide TCO materials, which offer the advantage of low synthesis temperatures and higher mechanical flexibility while maintaining high electrical quality.^3,4,7^ In recent decades, indium tin oxide and indium gallium zinc oxide have been extensively studied. As a result, a-IGZO has been achieved with a resistivity of around 10^–3^ Ω cm while exhibiting less than 10% absorption in the visible range of the light spectrum concurrently.^3,8−10^ Amorphous zinc–tin oxide (a-ZTO) has also been extensively studied and achieved a conductivity of about 450 S cm^–1^ while maintaining greater than 80% transparency in the visible region of the light spectrum.^11,12^ The search for more environmentally friendly substitutes was prompted by the shortage of indium material and concerns about environmental and health impacts.^13,14^ In this regard, one of the best alternatives is a-ZTO (a-ZnSnO), which is a low-cost and abundant compound material,^15^ which is already being used in solar cells and OLEDs, TFT, and displays.^2,5^ However, the key to the performance of ZnSnO has been the development of annealing approaches to control the defect profile of the films. This is particularly the case with films that are deposited at high temperatures, low temperatures, or at room temperature.^16−18^ The exact defect chemistry of a-ZTO is still being extensively investigated.^19^ However, most studies have been focused on measurements of the properties of a-ZTO films before and after annealing.^11,12,20−22^ Here, we present an in situ annealing study of ZTO, whose aim is to determine ideal annealing procedures for the films by examining the resistivity of the films during the annealing process. The changes in the conduction mechanisms will be examined for the first time. Finally, to ensure compatibility with cost-effective synthesis processes, the annealing temperatures are kept below 300 °C.

This work presents an in situ observed annealing study on a-ZTO to highlight the importance of the duration of the annealing time. To investigate the annealing temperature significance of the activation energy of the reaction that was derived from rearranged Arrhenius equation , where using ln(*R*_sh_) versus  graph slope. The highest region of activation energy of the reaction was found within a rapid decrease in the slope. This activation energy is used to determine the area of the greatest rating change over time. These studies allow us to select the lowest temperatures at which the largest changes in the resistivity of the samples occur by identifying a lower annealing temperature. We found a range of values where a slight improvement is achieved with increases in the annealing temperature. In this region, it was observed that an increase in the total annealing time served as a substitute for an increase in the temperature. Furthermore, we clearly show a change in the conduction mechanism of films after reaching the highest conductivity.

## 2 Experimental Details

Amorphous zinc-tin oxide (a-ZTO) films were synthesized by nonreactive radio frequency magnetron cosputtering. The films were sputtered using ZnO and SnO_2_ targets with a purity of 99.99% in an inert argon atmosphere. The power applied to the ZnO target was 28 W and to the SnO_2_ target was 50 W. At the same time, the total gas pressure of the sputtering chamber was kept constant at 1 × 10^–3^ mbar with a constant argon partial pressure. The base pressure of the sputtering chamber was 1 × 10^–5^ mbar. The substrate temperature used for all thin film deposits was 300 °C. The deposition temperature of 300 °C is currently regarded as one of the most effective synthesis temperatures for optimizing a-ZTO with our magnetron sputtering system. For this reason, this temperature was found to be suitable for the deposition of our best performing films as deposited in this work. However, a comprehensive optimization study is underway to find a low sputtering temperature in the future, and these optimized a-ZTO films will be used for annealing studies. The electrical properties of all a-ZTO films before and after annealing were determined by using the Hall measurement system. Namely, the carrier concentration, electron mobility, and resistivity of all films in this work were measured at room temperature using the Hall system in the Van der Pauw method, in which four contacts are contacted with silver wire and silver paint. These four contacts are placed at the four corners of the samples. The transmission and reflection spectra of the films were measured by using a PerkinElmer Lambda 650 UV–vis spectrometer. The thickness values of the films were measured by using X-ray reflectance (XRR) on a Bruker D8 Discover with a monochromatic Cu source. Meanwhile, the amorphous nature of the films was confirmed by X-ray diffraction (XRD) using a Bruker D8 Advance with a nonmonochromatic Cu source (XRD). UV–vis spectrometry, XRD, XRR, and other data can be found in the Supporting Information.

**Figure 1:**
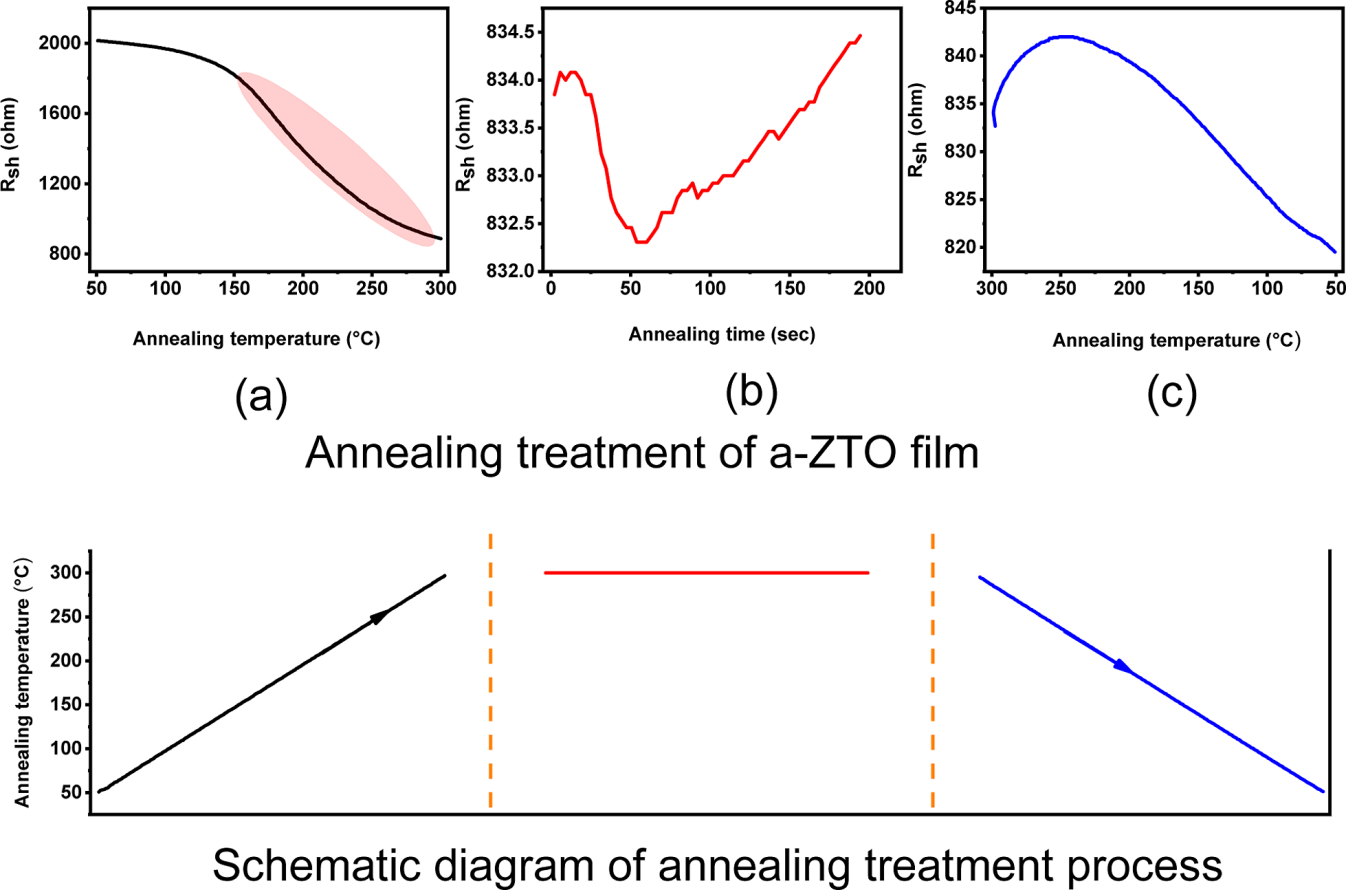
Schematic diagram of an in situ annealing method with a linear four-point probe: where (a) heating up from 50 °C to at a temperature, (b) maintain at the target temperature that continues to saturate the lowest sheet resistance, and (c) cooling down from the target temperature to 50 °C. Measurement progresses from (a) to (c) continuously and an example for 300 °C only. The lower schematic diagram corresponds to a representation of the annealing temperature cycles.

The sheet resistance change of the films was monitored in situ during annealing to achieve the best properties of the films. The annealing setup was also used in our previous works.^23−28^ The a-ZTO thin film was heated from 50 °C to an annealing temperature as shown in Figure 1a and then maintained at the target annealing temperature until the lowest resistivity was reached. Note that for this a-ZTO film, the annealing time for a stable temperature of 300 °C is about 50 s, but the annealing process took up to 200 s to ensure no improvement in the resistance slope as shown in Figure 1b. This means that the film reaches its maximum conductivity at a stable temperature of 300 °C without any decrease in the resistivity of the film, even if this continues for some time after reaching the lowest resistance. Finally, this film was allowed to cool from a stable temperature (300) to 50 °C at the same rate as the heating process, as shown in Figure 1c.
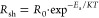
1
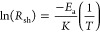
2

Figure 1a shows a change in the slope of the resistance enhancement curve (shaded red) that was found. The Arrhenius equation (eq 1 was applied to study the resistance curve. The activation energy of the reaction for a red-shaded region was determined using an equation as given (eq 2).

**Figure 2:**
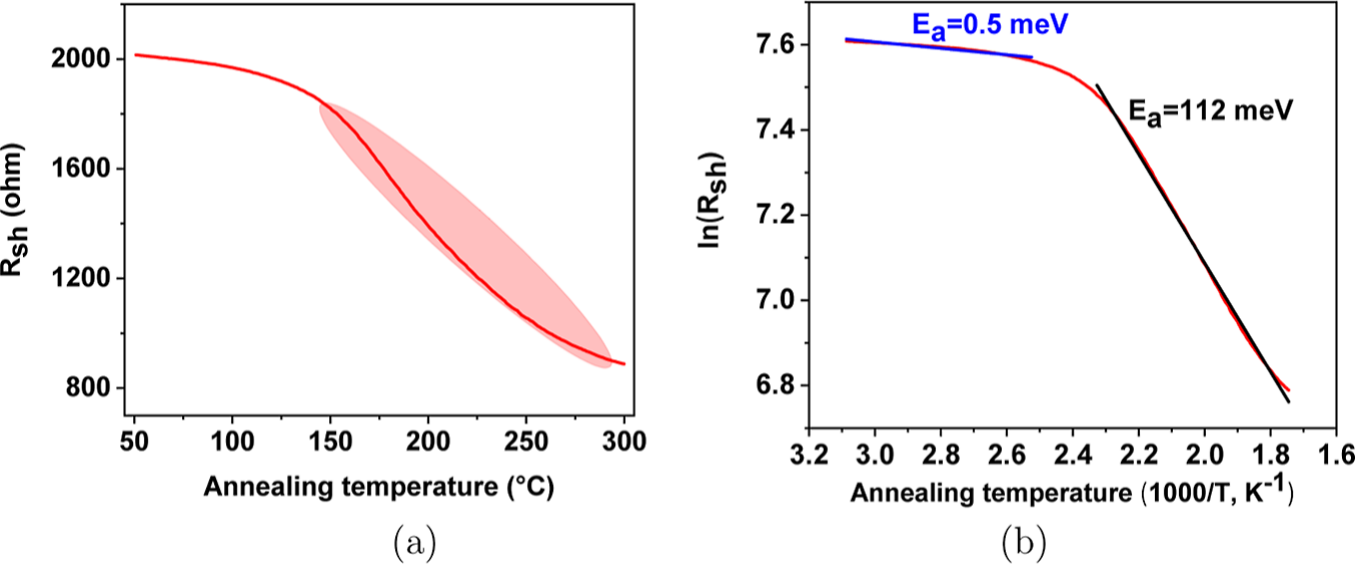
Method for calculating the activation energy of the reaction of a-ZTO annealed in a nitrogen environment: (a) *R*_sh_ versus annealing temperature and (b) calculation of activation energy using the Arrhenius equation (eq 2.

By examining *R*_sh_ versus the temperature slope in Figure 2a, it was found that the resistivity does not change significantly through thermal post-treatment in a nitrogen atmosphere up to annealing temperatures of 150 °C. In this temperature range, this is the same for all films in this work, and there is a reduction in resistivity consistent with the standard thermal activation of charge carriers in semiconductors. In all cases, the activation energy of the annealed samples is approximately 0.5 meV, an activation energy value is in line with a previous work, which was found in heavily doped a-ZTO.^29^ These positive activation energy values indicated that no films with degenerate doping behavior were observed before the lowest resistivity was achieved. A discussion on this matter will be given later.

Above the annealing temperature of 150 °C, there is a rapid change in slope in Figure 2b. This area is indicated by a shaded area in Figure 1a, and the largest activation energy value was found in this area; it can also be seen in Figure 1b. This indicates the onset of a secondary reaction in the film, the activation energy of which is significantly higher than that of thermal carrier excitation in semiconductors, while it may be the effect of a temperature increase on the conductivity of semiconductors. This reaction is observed to be irreversible, and the changes in resistance are permanent when cooling to room temperature, which indicates a fundamental change in sample properties. To determine the optimal low annealing temperature to achieve the highest conductivity values, we selected six different temperatures within the high activation energy region indicated by the red-shaded region in Figure 2.

All a-ZTO films deposited and annealed in this work show an amorphous state, which can be firmed by XRD data of films as given in the Supporting Information. Our previous annealed a-ZTO films showed even when a-ZTOs were annealed at 320 °C.^30^ The amorphous phase is consistent with other reports in which a-ZTO films annealed at 600 °C that do not show a crystalline structure in a previous work.^31^ To ensure the consistency of the annealing study, all samples were deposited with the same Zn/Sn ratio, suggesting that chemical composition plays a key role in the electrical properties of films, as a ZTO film with a high Sn concentration more charge carriers (conductive).^32^ Another meaning is to synthesize the average electrical properties of films, as shown in Figure 4. The chemical composition was studied by XPS and can be found in the Supporting Information. All films in this work were annealed in a nitrogen atmosphere with a purity of 99.99%.

## 3 Results and Discussion

To set baseline properties for thin films, the conductivity, carrier concentration, and carrier mobility properties of 38 nm-thick a-ZTO films were measured at room temperature. The average conductivity, carrier concentration, and mobility are individually about 120 S/cm, 6 × 10^19^ cm^–3^, and 12 cm^2^/(V s) as shown in Figure 4. As shown in the Supporting Information, all a-ZTO films exhibit high optical transparency (more than 80%) in the visible light spectrum.

**Figure 3:**
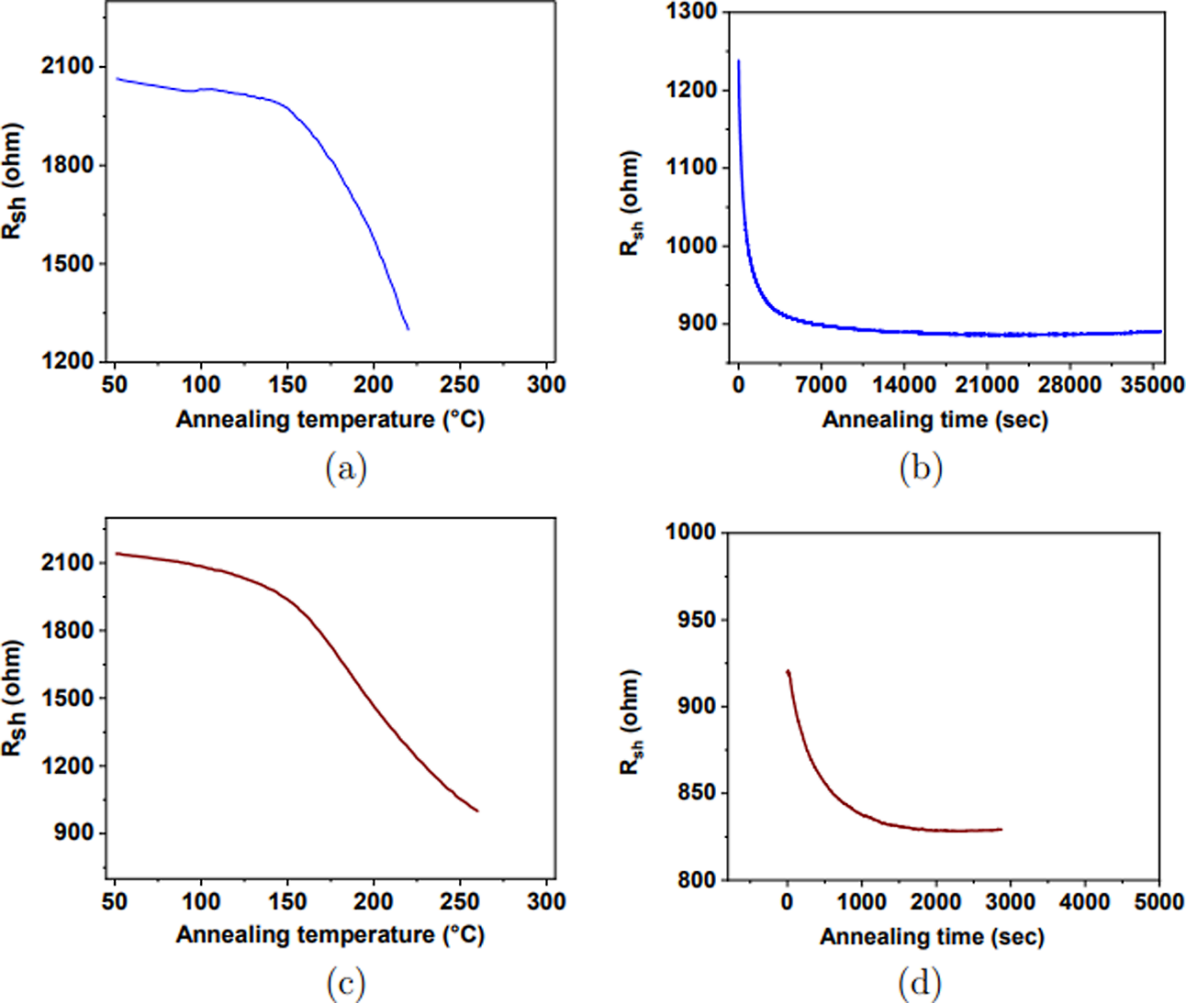
a-ZTO films annealed in a nitrogen atmosphere at 220 and 260 °C: (a,c) sheet resistance versus annealing temperature and (b,d) sheet resistance versus annealing time. Note that graphs of sheet resistance versus annealing temperature for other annealing temperatures can be seen in Supporting Information Figures S6 and S7.

All samples were annealed until the lowest sheet resistance was achieved in a nitrogen atmosphere, as described in Figure 1 above. This is also shown in Figure 3a,b for selected temperatures with in situ monitoring. Figure 3 shows that these two annealing times were 10 h 30 min for the annealing temperature 220 °C and 1 h 15 min for the annealing temperature 260 °C. This implies an increase in the annealing time while simultaneously reducing the annealing temperature or vice versa. These cases have shown that the annealing temperature 220 °C can be replaced by the annealing temperature 260 °C. One can compensate for lower annealing temperatures with increasing annealing time without sacrificing the final resistance values, as shown in Figure 3.

**Figure 4:**
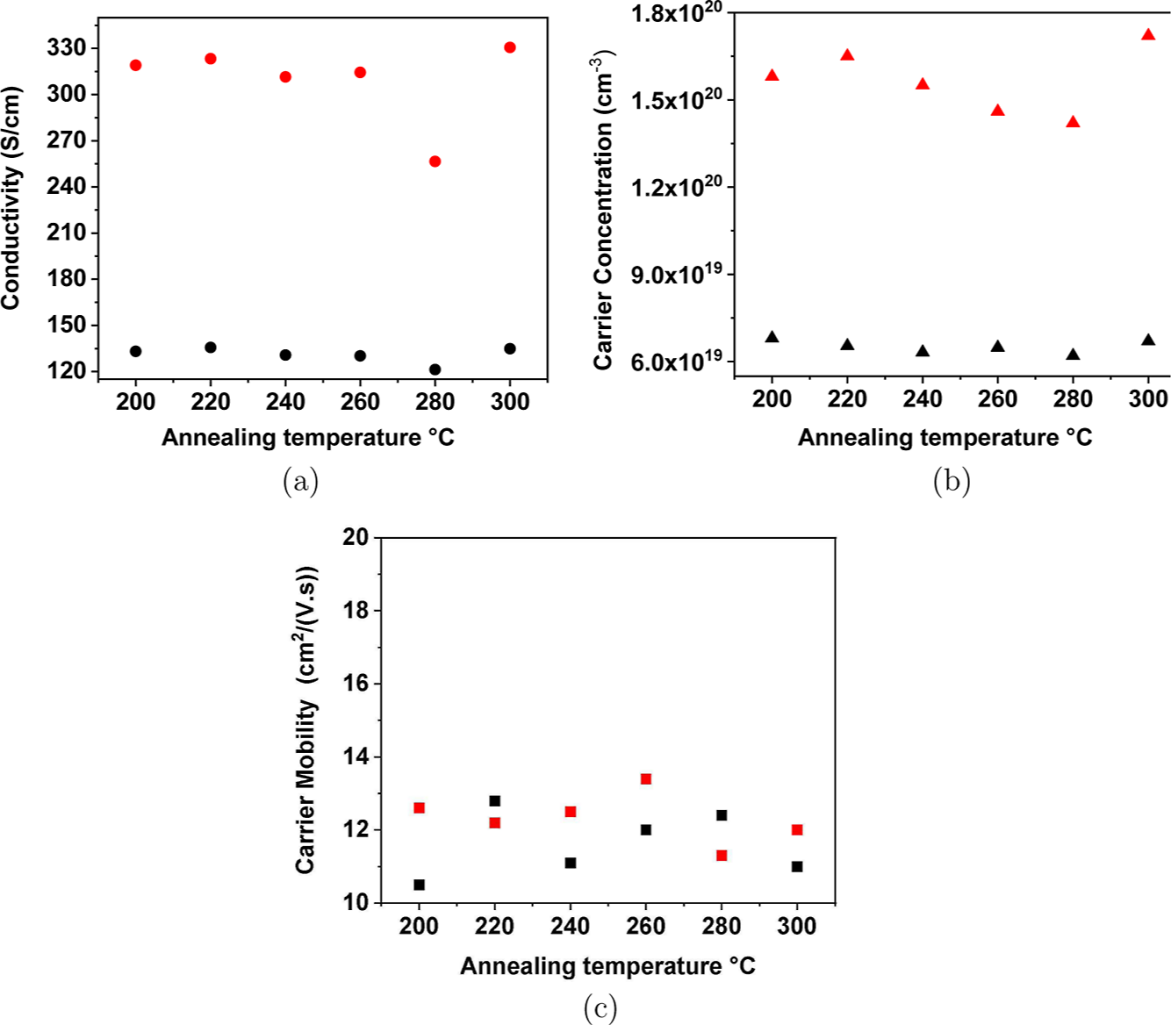
a-ZTO films annealed in a nitrogen atmosphere, (a) conductivity, (b) carrier concentration, and (c) carrier mobility. Here, (•), (▲), and (■) show the initial conductivity (a), carrier concentration (b), and carrier mobility (c) of as-deposited a-ZTO films, individually. Conductivity (a) (• red), carrier concentration (b) (▲ red), and carrier mobility (c) (■ red) for samples after annealing in a nitrogen atmosphere.

Figure 4a shows the conductivity and carrier concentration of these a-ZTO films as a function of annealing temperature and time. For each sample, the temperature was maintained until the resistivity reached a low value and saturated, and then, the temperature was lowered. At annealing temperatures in a nitrogen atmosphere (hereinafter referred to as an oxygen-poor environment), the carrier mobility is independent of the annealing temperatures and the atmosphere, as shown in Figure 4c, and only the carrier concentration increases after annealing. This mobility behavior is consistent with previous results showing that oxygen deficiencies and metal clusters act not only as carrier donors but also as scattering sites, thus limiting carrier mobility in the films. The results of a previous work suggest that electron mobility is increased by high-temperature annealing (>400 °C) in a high oxygen environment.^11,17,33^ The presence of metal vacancies (Sn–Zn or Sn–Sn defect complexes) and oxygen vacancies (V_O_) in a-ZTO is crucial for the electrical characteristics of a-ZTO films. Oxygen vacancies can act as shallow donors or acceptors in the ZTO lattice and lead to the generation of additional charge carriers (electrons or holes). Oxygen vacancies can also introduce localized defect states within the bandgap of a-ZTO, which can affect its electronic band structure.^34−37^ These defect states can trap charge carriers, alter charge carrier mobility, or influence recombination processes, thereby affecting the overall electrical performance of the material.^38^ It is worth noting that our annealing temperatures are lower than those in other studies, where the aim was to improve the carrier mobility of the mentioned films.^11,22,31^ Furthermore, annealing affects the intrinsic oxygen vacancies in the films observed in this letter and acts as charge carrier sources. This implies that annealing can modify the concentration of intrinsic oxygen within an amorphous network, which depends on the annealing atmosphere, meaning that the incorporation or change of oxygen concentration during annealing leads to variations in the electrical properties of the samples.^34^Figure 4c shows that the carrier mobility is a function of temperature; however, no improvement in the carrier mobility of the a-ZTO films was observed in this work because these films were grown in an oxygen-poor atmosphere and annealed at a low annealing temperature. It has been proven that the absence of oxygen cannot change the Zn and Sn scattering centers, Li et al. 2021 and Weng et al. 2019, which limits charge carrier mobility in oxide semiconductors.^36,37^ However, a comprehensive annealing study on a-ZTO films in an oxygen-rich atmosphere using our in situ annealing method will be discussed in our upcoming publication.

**Figure 5:**
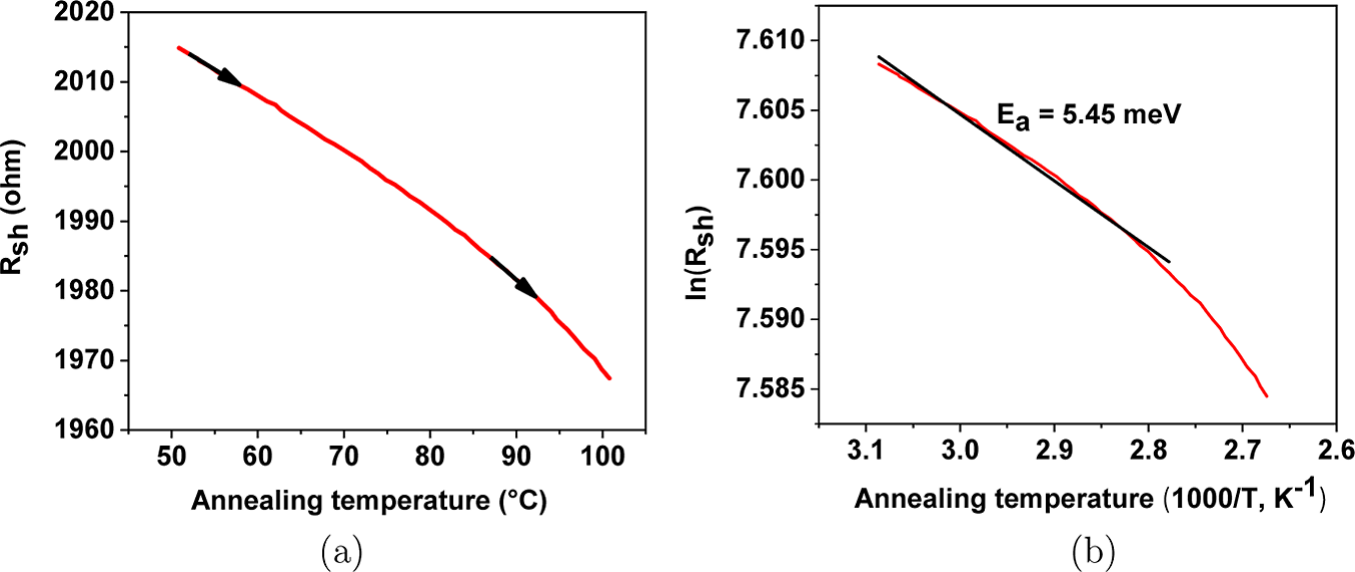
Illustration of *R*_sh_ versus annealing temperature slope for a-ZTO films prior to achieving the lowest resistivity between 50 and 100 °C: (a) sheet resistance versus annealing temperature slope between 50 and 100 °C and (b) activation energy for the slope between 50 and 100 °C.

Interestingly, it can be clearly seen from Figure 1c that the resistivity of the films gradually decreases as the temperature decreases. Therefore, the activation energies of six films were studied for annealing temperatures between 50 and 100 °C, which is before achieving the lowest resistivity, as shown in Figure 5.

**Figure 6:**
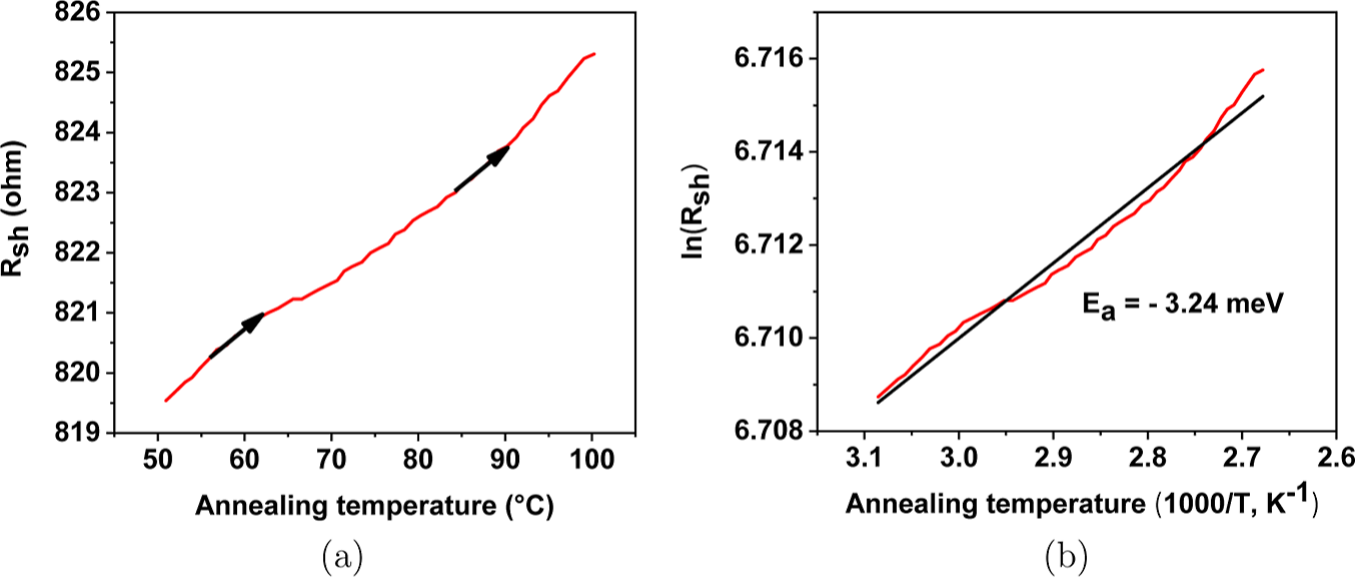
Annealing slope for a-ZTO films after achieving the lowest resistivity between 50 and 100 °C: (a) sheet resistance versus annealing temperature between 50 and 100 °C and (b) calculation of activation energy.

The positive values of activation energies were observed, as shown in Table 1. All positive values of activation energies imply that prior to achieving the highest conductivity, a-ZTO films showed a semiconducting behavior. The temperature range (between 50 and 100 °C) is relatively low. However, the elevated temperature activates a small number of electrons in the sample. The activation energies of six films after achieving the lowest resistivity were obtained, as shown in Table 1. *R*_sh_ versus annealing temperature slope is also shown in Figure 6a, where the increase in the anneal temperature leads to a decrease in conductivity.

**Table 1:** Activation Energy of Films Prior to and after Achieving the Lowest Resistivity of a-ZTO

a-ZTOs annealed at	200 °C	220 °C	240 °C	260 °C	280 °C	300 °C
prior to (meV)	5.1	4.8	4.62	5.51	5.45	5.45
after (meV)	–3.12	–3.58	–3.44	–1.67	–1.25	–3.24

The positive values of activation energies were observed as shown in Table 1. All positive values of activation energies indicate that a-ZTO films exhibit semiconducting behavior before achieving the highest conductivity. The temperature range between 50 and 100 °C is relatively low. However, the increased temperature activates a small number of electrons in the sample. The activation energies of six films after reaching the lowest resistivity were obtained as shown in Table 1. The slope of *R*_sh_ as a function of annealing temperature is also shown in Figure 6a, where the increase in the annealing temperature leads to a decrease in conductivity.

This means that the samples in this work exhibit degenerate-doped behavior that is similar to the metallic conductivity behavior. This can be explained by an increase in temperature with increasing number of ionized atoms, which result in a rapidly decreasing resistance which was confirmed in previous reports.^22,39^ Since the annealing temperature is sufficiently high, a large part of the dopants is completely ionized, which leads to an increase in resistivity with temperature in films, like metals. It is known that in metals, conductivity decreases with increasing temperature due to a high frequency of free electron collisions.^40^ It is proven that a-ZTO contains oxygen vacancies (V_O_) and metal clusters (Sn–Zn or Sn–Sn defect complexes), which play a key role in the electrical properties of a-ZTO films. These defect complexes are characterized by Sn and Zn, as well as oxygen. However, previous density functional theories showed^41^ that oxygen-deficient samples have such 1 Sn defects comparable to our a-ZTO films because our samples were deposited and annealed in an oxygen-deficient atmosphere. All indicate that Sn is a carrier driver, and Zn increases the carrier mobility in a-ZTO films. In our scenario, the carrier concentrations in a-ZTO films rise as the temperature or time increases. This suggests that more Sn and oxygen vacancies are probably becoming ionized rather than Zn ions.^42^ This implies that changes in the slope of *R*_sh_ versus the annealing temperature indicate that the conduction mechanism of films has changed, as shown in Figure 6a.

As shown in Table 1, it can also be taken into account that the activation energies of films with different values were found after achieving the lowest resistivity because samples were degenerated with different levels by six chosen temperatures. Sample annealed at 220 °C is more degenerated than sample annealed at 280 °C, and that is why the sample annealed at 280 °C shows less improvements in the final resistivity. This can be seen from the final carrier concentration of the films, as shown in Figure 4. To understand this, an extensive investigation of annealing temperature 280 °C needs to be studied due to its close temperature to the synthesis temperature. In this report, the aim is to find a lower annealing temperature and study the desirable properties of films by this annealing method; therefore, this region was not focused on. However, a lower annealing temperature should be suitable for specific substrates and device structures. An example would be in the case of polyimides, which can exhibit degradation at temperatures above 260 °C. Flexible polyethylene terephthalate (PET) and polyethylene furanoate (PEF) substrates need to keep the process temperature below the 250 °C, and therefore, annealing at >220 °C is desirable to enhance the electrical properties of films.^43,44^

## 4 Conclusions

In summary, this study developed a method for real-time in situ monitoring of resistance changes during the annealing process. This annealing approach was applied to study the annealing effects on amorphous zinc–tin oxide (a-ZTO) films, with the aim of improving their properties while keeping sample processing temperatures low. We focused our analysis on determining the most significant rate of change of sheet resistance in the temperature range from 220 to 300 °C. At the same time, we extracted activation energies within the temperature range with a large change in resistivity during the annealing process. In this temperature range, six temperatures were selected, and an annealing study on a-ZTO films was carried out. We used the data to optimize the annealing conditions to achieve excellent sample properties. Remarkably, by annealing at 220 °C, we achieved a significant improvement in conductivity, obtaining approximately 320 S/cm in 38 nm-thick a-ZTO films. It is noteworthy that this result was repeated when annealing at a higher temperature of 300 °C. This implies that annealing at lower temperatures can be compensated for by increasing the annealing time, resulting in comparable improvements in electrical properties. Furthermore, for all films, the remarkable observation was made that the annealing process progressed with a decrease in resistivity that was irreversible and the minimum resistance was reached. All a-ZTO films in this work changed from a semiconducting to a metallic state. This fascinating shift illustrates that the annealing treatment fundamentally changes the conduction mechanism of these films.

## Supporting Information Available

Examining the desirable properties of ZnSnO_y_ by annealing treatment with a real-time observation of resistivity (ZIP)
XPS data of the films; UV–vis spectra of films; a-ZTO bandgap; SEM data; XRR and XRD data; and annealing graphs (PDF)

## References

[ref1] Kamiya T.; Nomura K.; Hosono H. Present status of amorphous In-Ga-Zn-O thin-film transistors. Sci. Technol. Adv. Mater. 2010, 11, 044305 10.1088/1468-6996/11/4/044305. 27877346 PMC5090337

[ref2] Ellmer K.; Bikowski A. Intrinsic and Extrinsic Doping of ZnO and ZnO Alloys. J. Phys. D: Appl. Phys. 2016, 49, 413002 10.1088/0022-3727/49/41/413002.

[ref3] Chiang H. Q.; Wager J. F.; Hoffman R. L.; Jeong J.; Keszler D. A. High Mobility Transparent Thin-Film Transistors with Amorphous Zinc Tin Oxide Channel Layer. Appl. Phys. Lett. 2005, 86, 013503 10.1063/1.1843286.

[ref4] Nomura K.; Ohta H.; Takagi A.; Kamiya T.; Hirano M.; Hosono H.; Nomura K.; Ohta H.; Takagi A.; Kamiya T.; Hirano M.; Hosono H. Room-temperature fabrication of transparent flexible thin-film transistors using amorphous oxide semiconductors. Nature 2004, 432, 488–492. 10.1038/nature03090. 15565150

[ref5] Farrell L.; Norton E.; Smith C. M.; Caffrey D.; Shvets I. V.; Fleischer K. Synthesis of Nanocrystalline Cu Deficient CuCrO_2_ – A High Figure of Merit p-type Transparent Semiconductor. J. Mater. Chem. C 2016, 4, 126–134. 10.1039/C5TC03161C.

[ref6] Mauit O.; Caffrey D.; Ainabayev A.; Kaisha A.; Toktarbaiuly O.; Sugurbekov Y.; Sugurbekova G.; Shvets I. V.; Fleischer K. Growth of ZnO:Al by atomic layer deposition: Deconvoluting the contribution of hydrogen interstitials and crystallographic texture on the conductivity. Thin Solid Films 2019, 690, 137533 10.1016/j.tsf.2019.137533.

[ref7] Mereu R. A.; Le Donne A.; Trabattoni S.; Acciarri M.; Binetti S. Comparative study on structural, morphological and optical properties of Zn2SnO4 thin films prepared by r.f. sputtering using Zn and Sn metal targets and ZnO-SnO2 ceramic target. J. Alloys Compd. 2015, 626, 112–117. 10.1016/j.jallcom.2014.11.150.

[ref8] Ellmer K. Past achievements and future challenges in the development of optically transparent electrodes. Nat. Photonics 2012, 6, 809–817. 10.1038/nphoton.2012.282.

[ref9] Kim D.; Kim Y. G.; Kang B. H.; Lee J. H.; Chung J.; Kim H. J. Fabrication of indium gallium zinc oxide phototransistors: Via oxide-mesh insertion for visible light detection. J. Mater. Chem. C 2020, 8, 165–172. 10.1039/C9TC04982G.

[ref10] Lee D.-H.; Han S.-Y.; Herman G. S.; Chang C.-H. Inkjet Printed High-Mobility Indium Zinc Tin Oxide Thin Film Transistors. J. Mater. Chem. 2009, 19, 3135 10.1039/b822893k.

[ref11] Rucavado E.; Jeangros Q.; Urban D. F.; Holovský J.; Remes Z.; Duchamp M.; Landucci F.; Dunin-Borkowski R. E.; Körner W.; Elsässer C.; Hessler-Wyser A.; Morales-Masis M.; Ballif C. Enhancing the optoelectronic properties of amorphous zinc tin oxide by subgap defect passivation: A theoretical and experimental demonstration. Phys. Rev. B 2017, 95, 245204–245210. 10.1103/physrevb.95.245204.

[ref12] Lee S. M.; Joo Y. H.; Kim C. I. Influences of film thickness and annealing temperature on properties of sol-gel derived ZnO-SnO 2 nanocomposite thin film. Appl. Surf. Sci. 2014, 320, 494–501. 10.1016/j.apsusc.2014.09.099.

[ref13] Wäger P. A.; Lang D. J.; Wittmer D.; Bleischwitz R.; Hagelüken C. Towards a more sustainable use of scarce metals. A review of intervention options along the metals life cycle. Gaia 2012, 21, 300–309. 10.14512/gaia.21.4.15.

[ref14] Hines C. J.; Roberts J. L.; Andrews R. N.; Jackson M. V.; Deddens J. A. Use of and occupational exposure to indium in the United States. J. Occup. Environ. Hyg. 2013, 10, 723–733. 10.1080/15459624.2013.836279. 24195539 PMC4476525

[ref15] Niang K. M.; Cho J.; Sadhanala A.; Milne W. I.; Friend R. H.; Flewitt A. J. Zinc tin oxide thin film transistors produced by a high rate reactive sputtering: Effect of tin composition and annealing temperatures. Phys. Status Solidi A 2017, 214, 1600470 10.1002/pssa.201600470.

[ref16] Fernandes C.; Santa A.; Santos A.; Bahubalindruni P.; Deuermeier J.; Martins R.; Fortunato E.; Barquinha P. A Sustainable Approach to Flexible Electronics with Zinc-Tin Oxide Thin-Film Transistors. Adv. Electron. Mater. 2018, 4, 1–10. 10.1002/aelm.201800032.

[ref17] Troughton J.; Atkinson D.; Atkinson D. Amorphous InGaZnO and metal oxide semiconductor devices: an overview and current status. J. Mater. Chem. C 2019, 7, 12388–12414. 10.1039/c9tc03933c.

[ref18] Seo S. J.; Hwang Y. H.; Bae B. S. Postannealing process for low temperature processed sol-gel zinc tin oxide thin film transistors. Electrochem. Solid-State Lett. 2010, 13, 357–360. 10.1149/1.3474606.

[ref19] Shah A. Thin-Film Silicon Solar Cells; EPFL Press: Lausanne, Switzerland, 2010; pp 1–488.

[ref20] Kumar N.; Joshi B.; Asokan K. The effects of thermal annealing on the structural and electrical properties of zinc tin oxide thin films for transparent conducting electrode applications. Phys. B 2019, 558, 5–9. 10.1016/j.physb.2019.01.016.

[ref21] Jain V. K.; Kumar P.; Kumar M.; Jain P.; Bhandari D.; Vijay Y. K. Study of post annealing influence on structural, chemical and electrical properties of ZTO thin films. J. Alloys Compd. 2011, 509, 3541–3546. 10.1016/j.jallcom.2010.10.212.

[ref22] Ahn B. D.; Choi D. W.; Choi C.; Park J. S. The effect of the annealing temperature on the transition from conductor to semiconductor behavior in zinc tin oxide deposited atomic layer deposition. Appl. Phys. Lett. 2014, 105, 092103 10.1063/1.4895102.

[ref23] Biswas P.; Ainabayev A.; Zhussupbekova A.; Jose F.; O’Connor R.; Kaisha A.; Walls B.; Shvets I. V. Tuning of oxygen vacancy-induced electrical conductivity in Ti-doped hematite films and its impact on photoelectrochemical water splitting. Sci. Rep. 2020, 10, 7463–7510. 10.1038/s41598-020-64231-w. 32366858 PMC7198511

[ref24] Ali D.; Butt M. Z.; Coughlan C.; Caffrey D.; Shvets I. V.; Fleischer K. Nitrogen grain-boundary passivation of In-doped ZnO transparent conducting oxide. Phys. Rev. Mater. 2018, 2, 043402–043410. 10.1103/physrevmaterials.2.043402.

[ref25] Ainabayev A.; Mullarkey D.; Walls B.; Caffrey D.; Zhussupbekov K.; Zhussupbekova A.; Ilhan C.; Kaisha A.; Biswas P.; Tikhonov A.; Murtagh O.; Shvets I. Epitaxial Grown VO2 with Suppressed Hysteresis and Low Room Temperature Resistivity for High-Performance Thermal Sensor Applications. ACS Appl. Nano Mater. 2023, 6, 2917–2927. 10.1021/acsanm.2c05297.

[ref26] Syrlybekov A.; Arca E.; Verre R.; O Coileain C.; Toktarbaiuly O.; Khalid A.; Zhang H.; Shvets I. V. Induced morphological changes on vicinal MgO (100) subjected to high-temperature annealing: step formation and surface stability. Surf. Interface Anal. 2015, 47, 969–977. 10.1002/sia.5805.

[ref27] Toktarbaiuly O.; Syrlybekov A.; Nuraje N.; Sugurbekova G.; Shvets I. V. Surface faceting of vicinal SrTiO3(100). Mater. Today: Proc. 2022, 71, 69–77. 10.1016/j.matpr.2022.08.283.

[ref28] Kaisha A. Controlling the Properties of a-ZTO and a-IGZO via Low Temperature Annealing and Novel Layered Structures. Ph.D. Thesis, Trinity College Dublin, The University of Dublin, 2023.

[ref29] Hu W.; Peterson R. L. Charge transport in solution-processed zinc tin oxide thin film transistors. J. Mater. Res. 2012, 27, 2286–2292. 10.1557/jmr.2012.134.

[ref30] Zhussupbekova A.; Kaisha A.; Vijayaraghavan R. K.; Shvets I. V.; Caffrey D. Importance of Local Bond Order to Conduction in Amorphous, Transparent, Conducting Oxides: The Case of Amorphous ZnSnOy. ACS Appl. Mater. Interfaces 2019, 11, 44399 10.1021/acsami.9b06210. 31638369

[ref31] Choi Y. Y.; Kang S. J.; Kim H. K. Rapid thermal annealing effect on the characteristics of ZnSnO3 films prepared by RF magnetron sputtering. Curr. Appl. Phys. 2012, 12, 104–107. 10.1016/j.cap.2012.05.014.

[ref32] Park B.; Nam S.; Kang Y.; Jeon S.-P.; Jo J.-W.; Park S. K.; Kim Y.-H. Cation Doping Strategy for Improved Carrier Mobility and Stability in Metal-Oxide Heterojunction Thin-Film Transistors. Mater. Today Electron. 2024, 8, 100090 10.1016/j.mtelec.2024.100090.

[ref33] Wahila M. J.; Lebens-Higgins Z. W.; Butler K. T.; Fritsch D.; Treharne R. E.; Palgrave R. G.; Woicik J. C.; Morgan B. J.; Walsh A.; Piper L. F. Accelerated optimization of transparent, amorphous zinc-tin-oxide thin films for optoelectronic applications. APL Mater. 2019, 7, 022509 10.1063/1.5053683.

[ref34] Sung N. E.; Lee H. K.; Chae K. H.; Singh J. P.; Lee I. J. Correlation of oxygen vacancies to various properties of amorphous zinc tin oxide films. J. Appl. Phys. 2017, 122, 085304 10.1063/1.5000138.

[ref35] Kim W.; Kang S.; Lee Y.; Mun S.; Choi J.; Lee S.; Hwang C. S. Electrical properties of amorphous Zn-Sn-O thin films depending on composition and post-deposition annealing temperature near crystallization temperature. J. Mater. Chem. C 2023, 11, 8254–8262. 10.1039/D2TC05090K.

[ref36] Li Z.; Wu Z.; Wang X.; Cao H.; Liang L.; Yang J.; Song Y. Ultrafast carrier dynamics of amorphous zinc tin oxide graded thin films. J. Phys. Chem. C 2021, 125, 9350–9355. 10.1021/acs.jpcc.0c10511.

[ref37] Weng S.; Chen R.; Zhong W.; Deng S.; Li G.; Yeung F. S. Y.; Lan L.; Chen Z.; Kwok H. S. High-Performance Amorphous Zinc-Tin-Oxide Thin-Film Transistors with Low Tin Concentration. IEEE J. Electron Devices Soc. 2019, 7, 632–637. 10.1109/JEDS.2019.2919424.

[ref38] Gunkel F.; Christensen D. V.; Chen Y. Z.; Pryds N. Oxygen vacancies: The (in)visible friend of oxide electronics. Appl. Phys. Lett. 2020, 116, 120505 10.1063/1.5143309.

[ref39] Colinge J. P.; Colinge C. A. Physics of Semiconductor Devices by J. P. Colinge and; Kluwer Academic Publishers: New York Boston Dordrecht, London Moscow, 2002; pp 1–451.

[ref40] Pierret R.; Purdue C. E. Semiconductor Device Fundamentals; School of Engineering University: New York, 1996; pp 1–792.

[ref41] Körner W.; Urban D. F.; Ramo D. M.; Bristowe P. D.; Elsässer C. Prediction of subgap states in Zn- and Sn-based oxides using various exchange-correlation functionals. Phys. Rev. B: Condens. Matter Mater. Phys. 2014, 90, 195142 10.1103/PhysRevB.90.195142.

[ref42] Liu L. C.; Chen J. S.; Jeng J. S. Role of oxygen vacancies on the bias illumination stress stability of solution-processed zinc tin oxide thin film transistors. Appl. Phys. Lett. 2014, 105, 4–7. 10.1063/1.4890579.

[ref43] Yang W.; List-Kratochvil E. J.; Wang C. Metal particle-free inks for printed flexible electronics. J. Mater. Chem. C 2019, 7, 15098–15117. 10.1039/C9TC05463D.

[ref44] Zardetto V.; Brown T. M.; Reale A.; Di Carlo A. Substrates for flexible electronics: A practical investigation on the electrical, film flexibility, optical, temperature, and solvent resistance properties. J. Polym. Sci., Part B: Polym. Phys. 2011, 49, 638–648. 10.1002/polb.22227.

