# Supplementary figures and images for: Examining the Desirable Properties of ZnSnOy by Annealing Treatment with a Real-Time Observation of Resistivity
Source: ACS Omega. 2024 Jun 5;9(24):26205–12. doi: 10.1021/acsomega.4c01857 (PMC11191124; doi:10.1021/acsomega.4c01857)

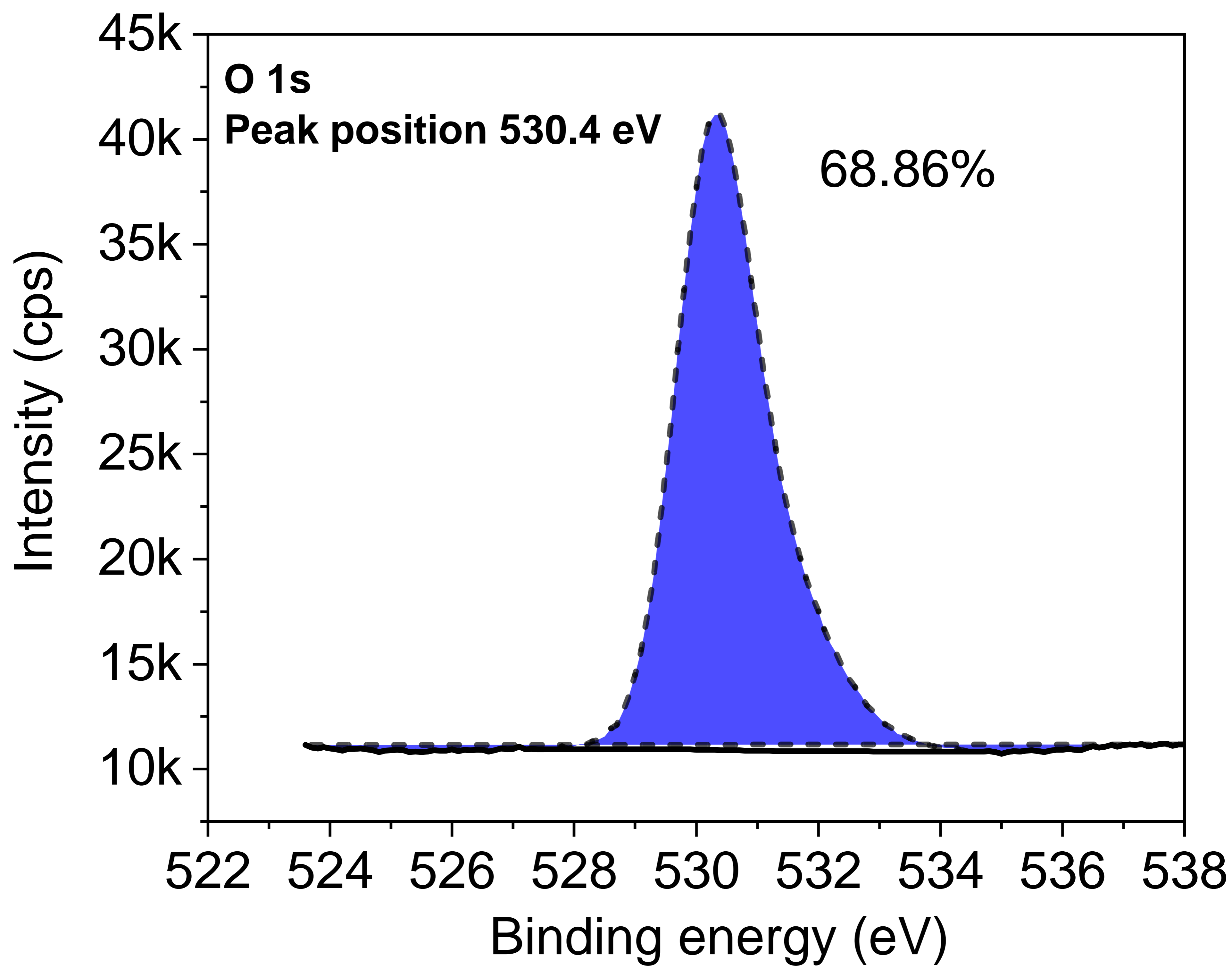

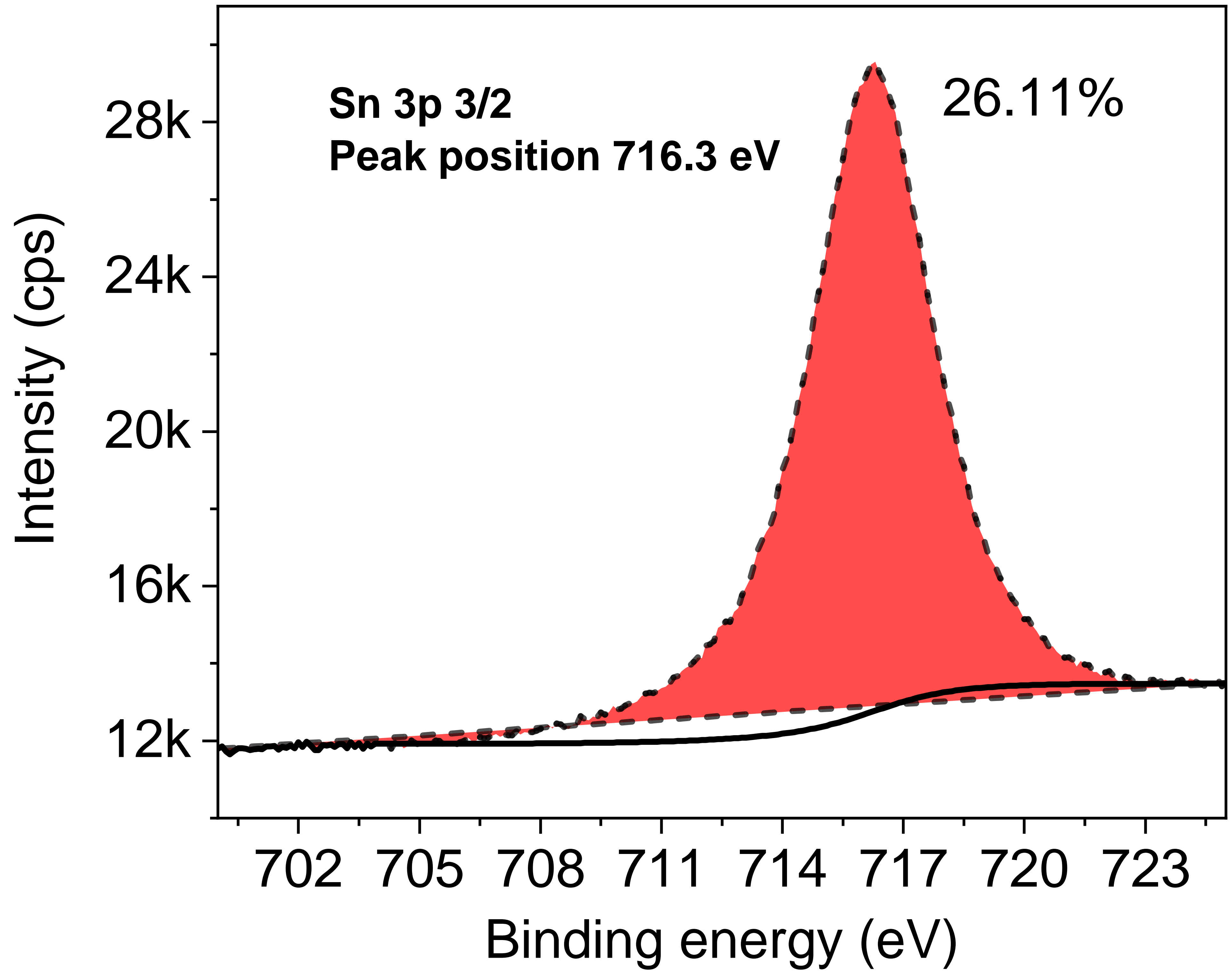

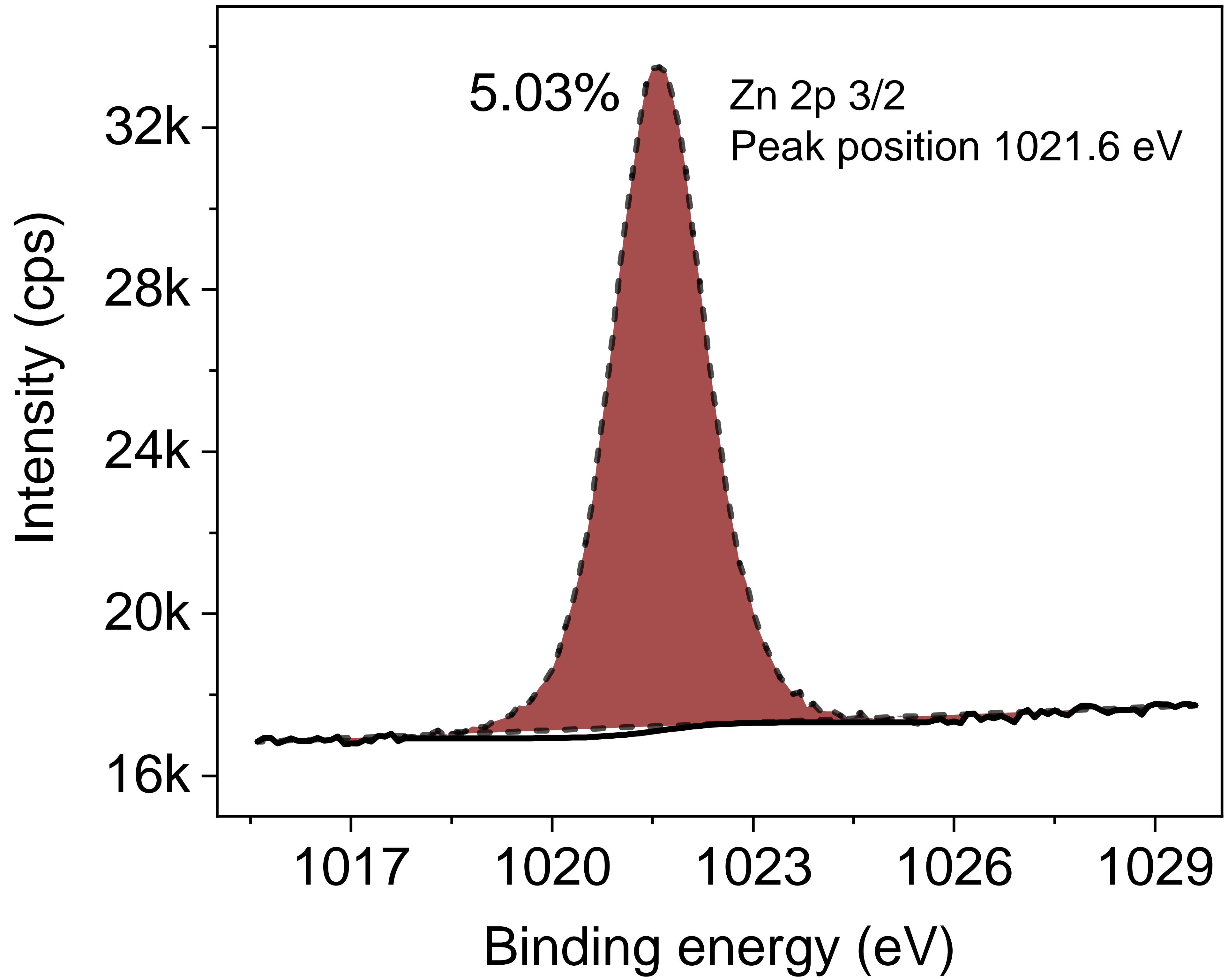

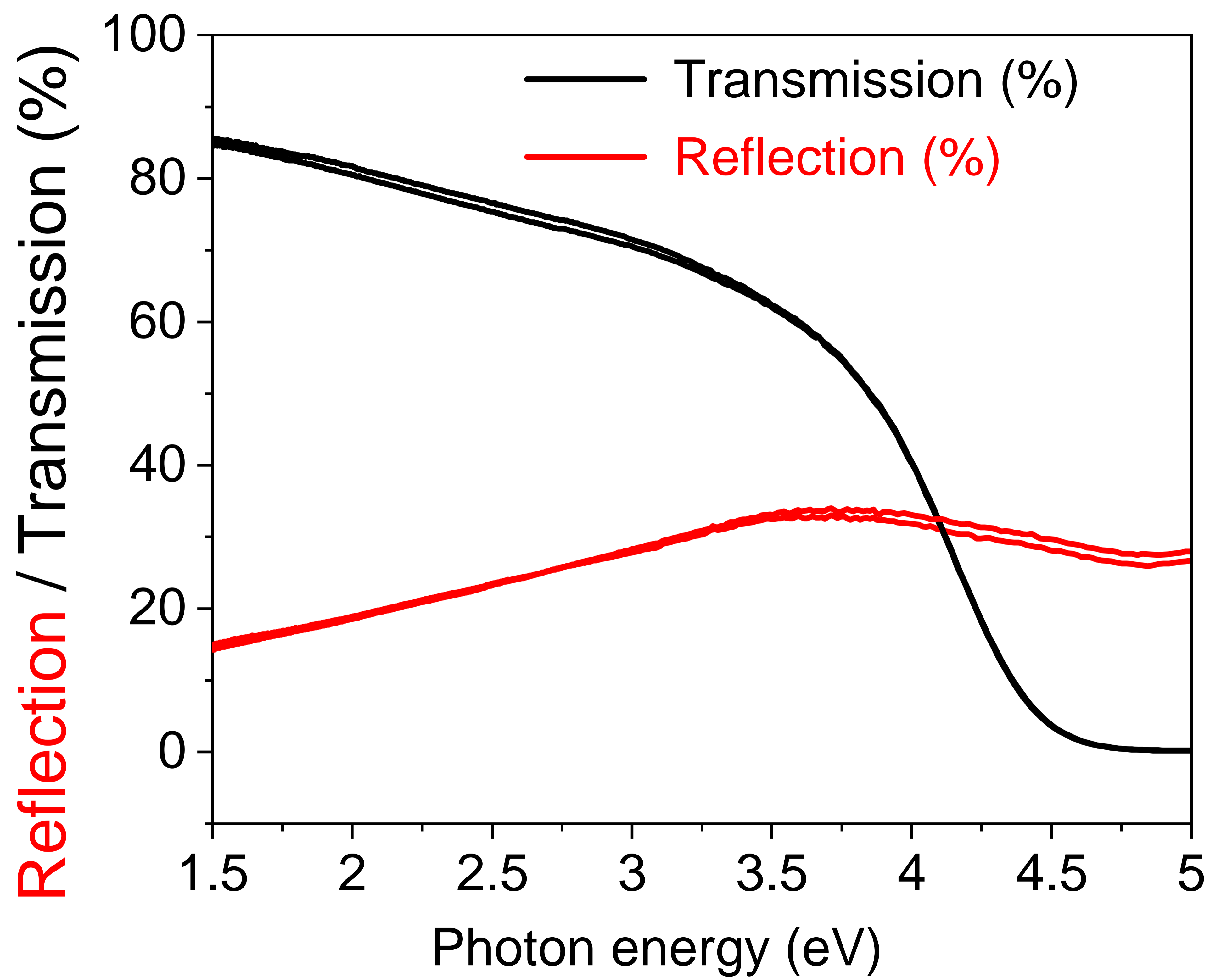

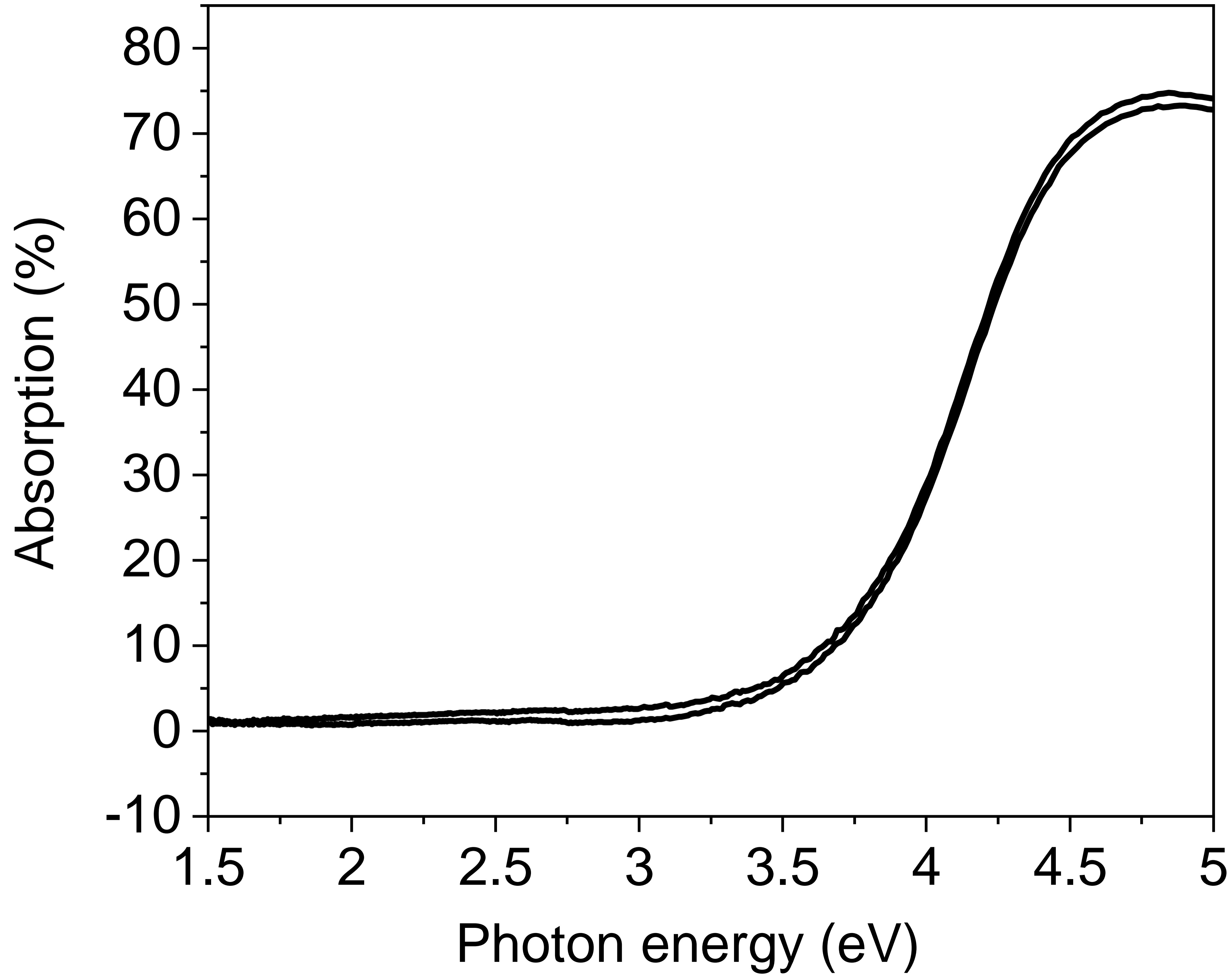

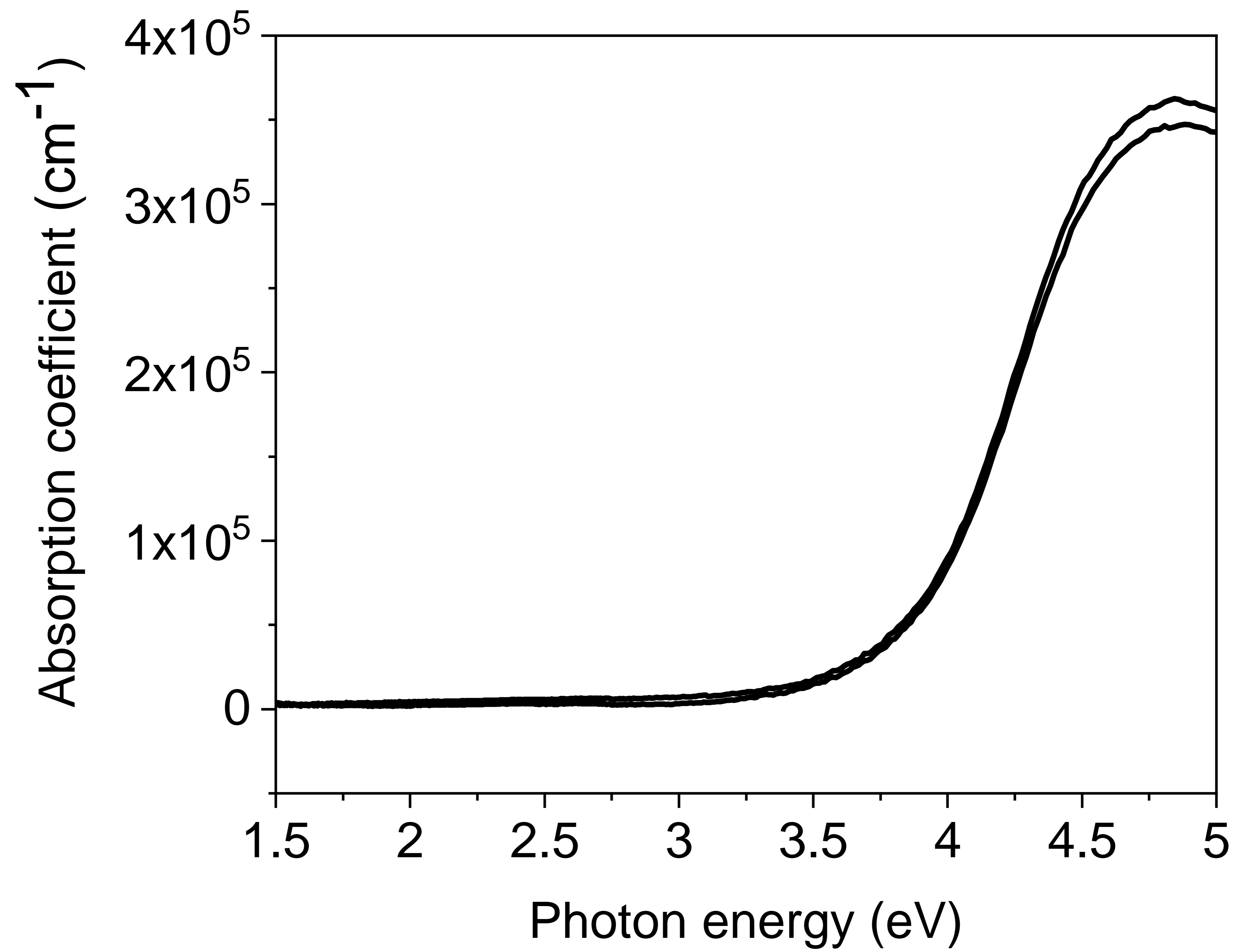

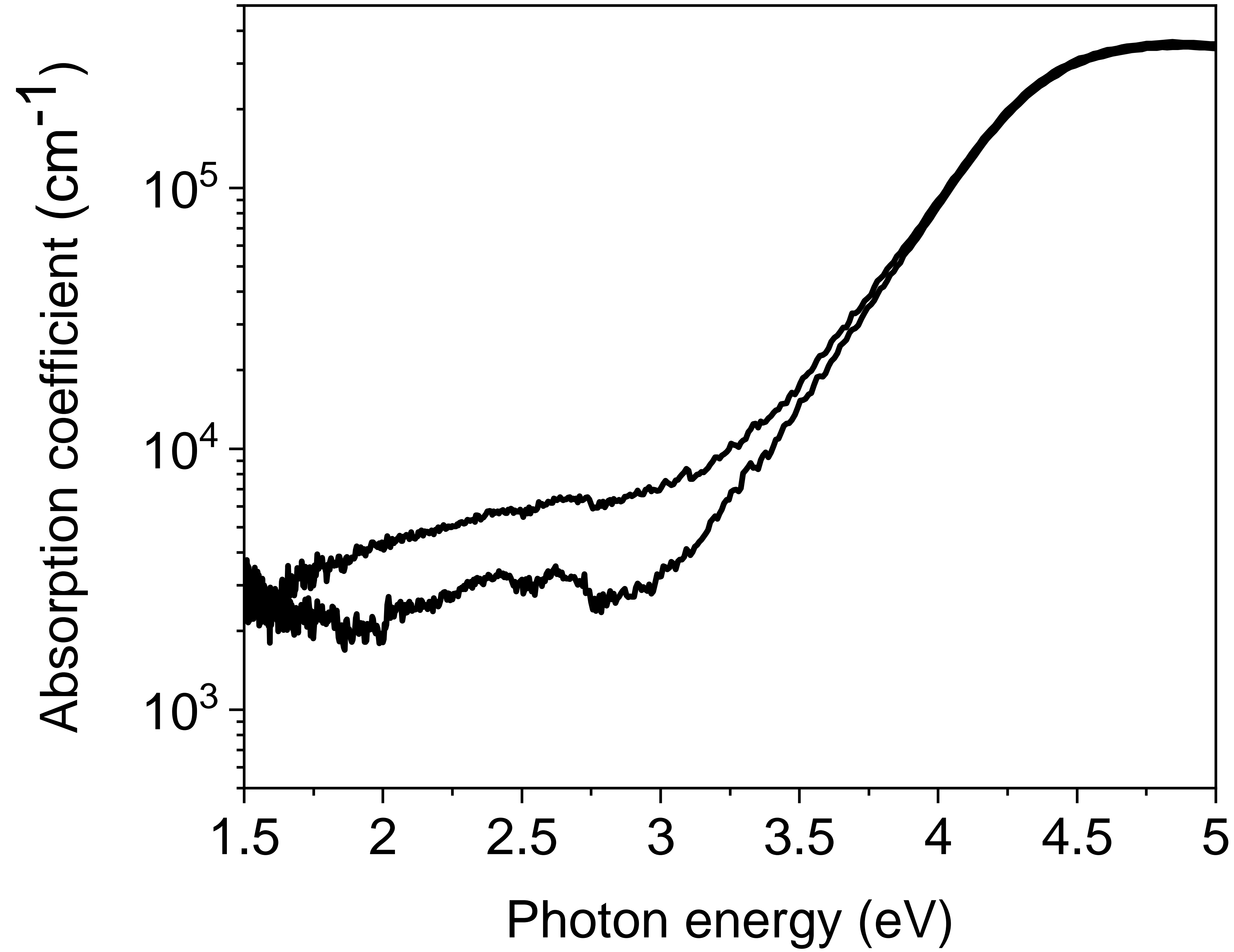

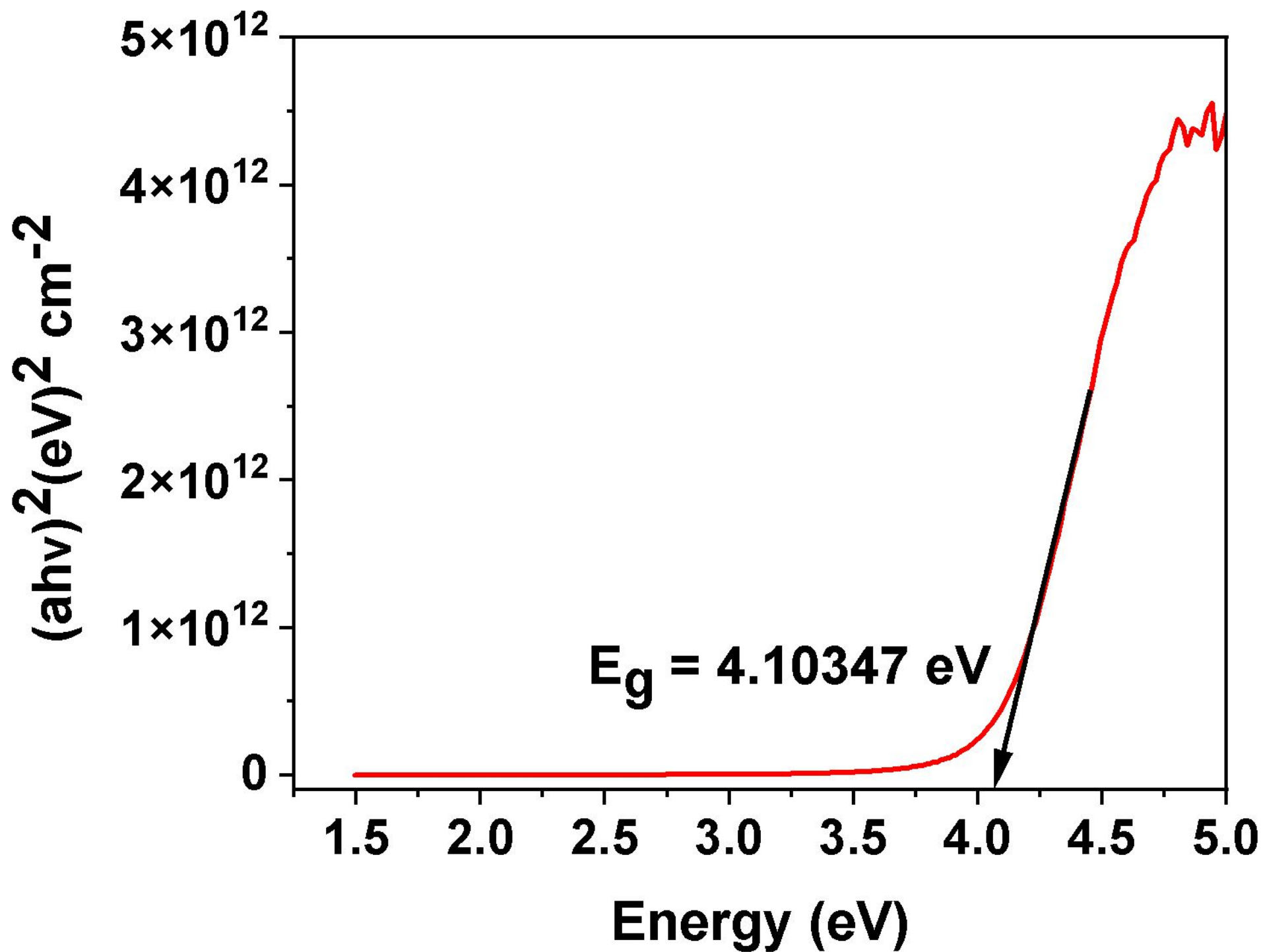

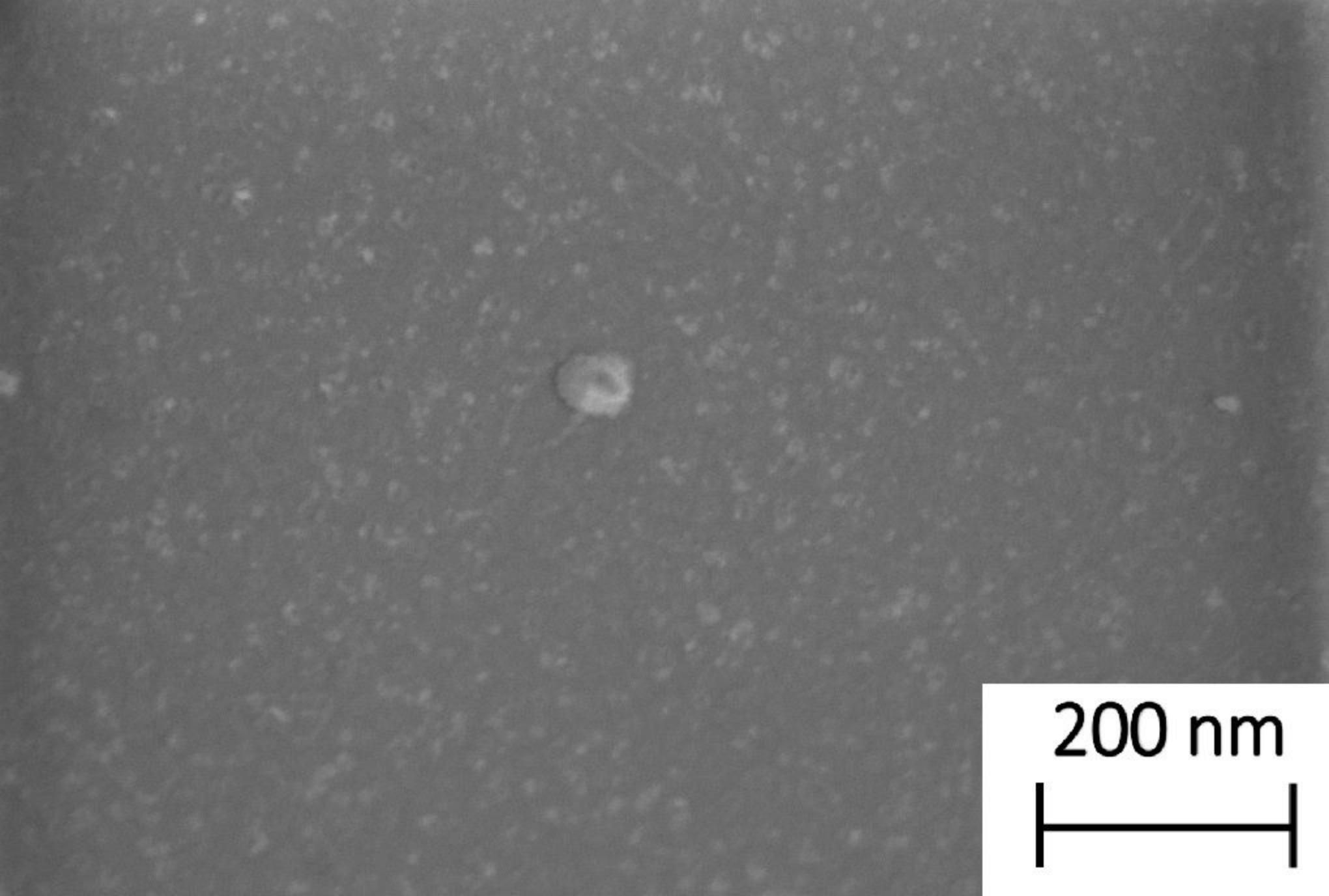

200 nm

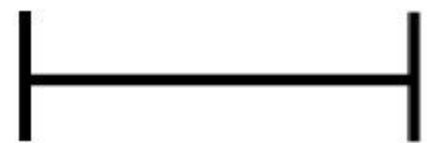

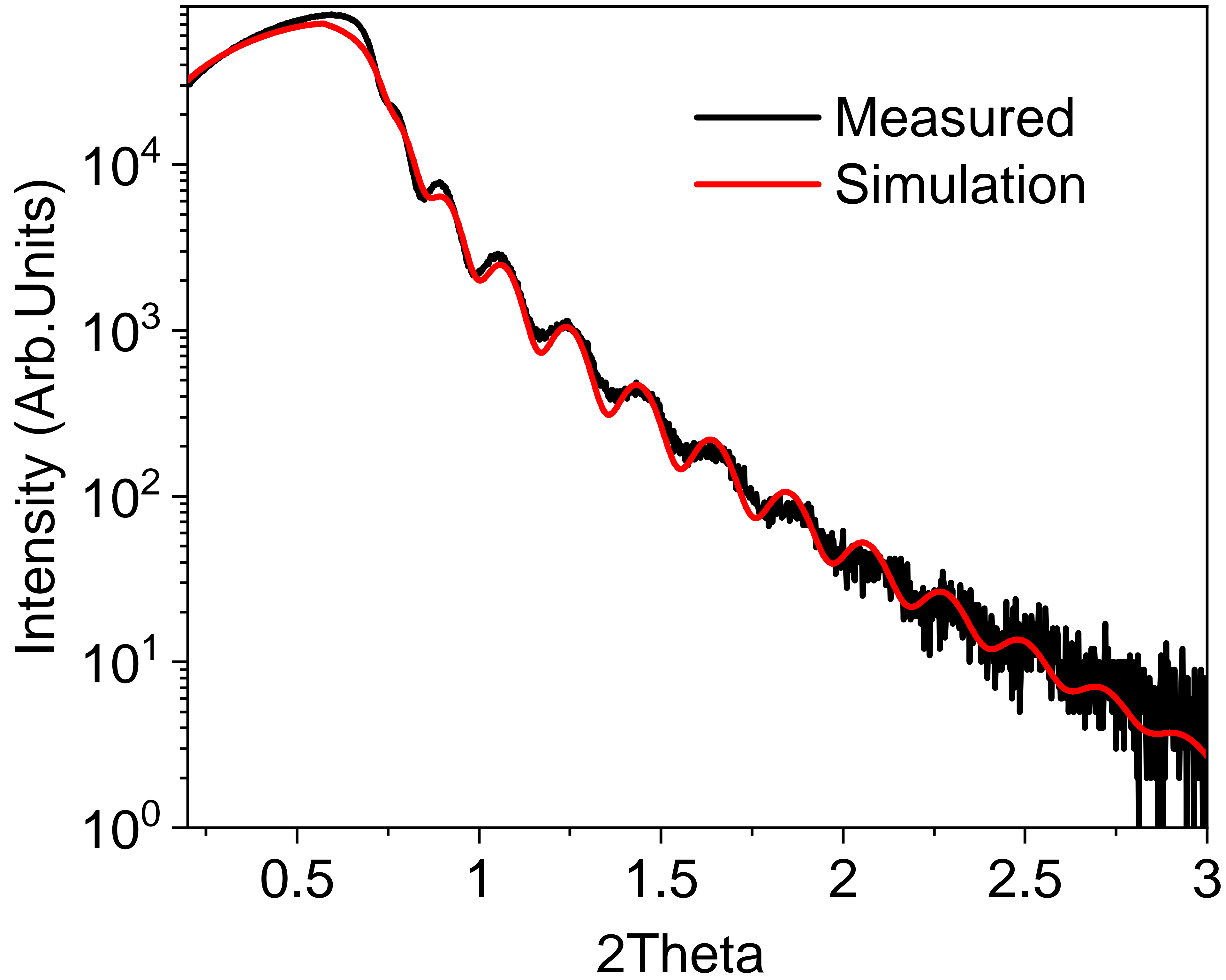

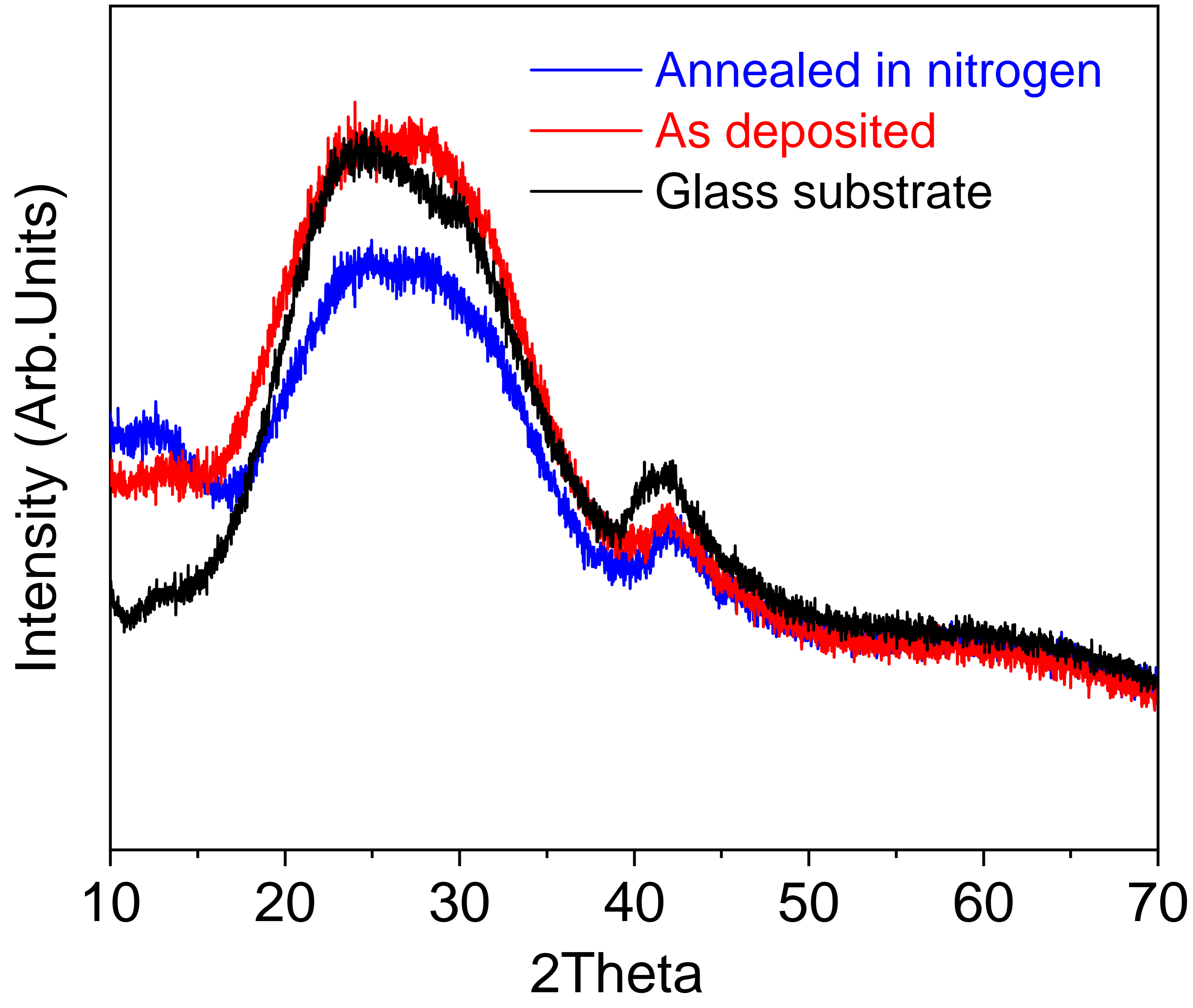

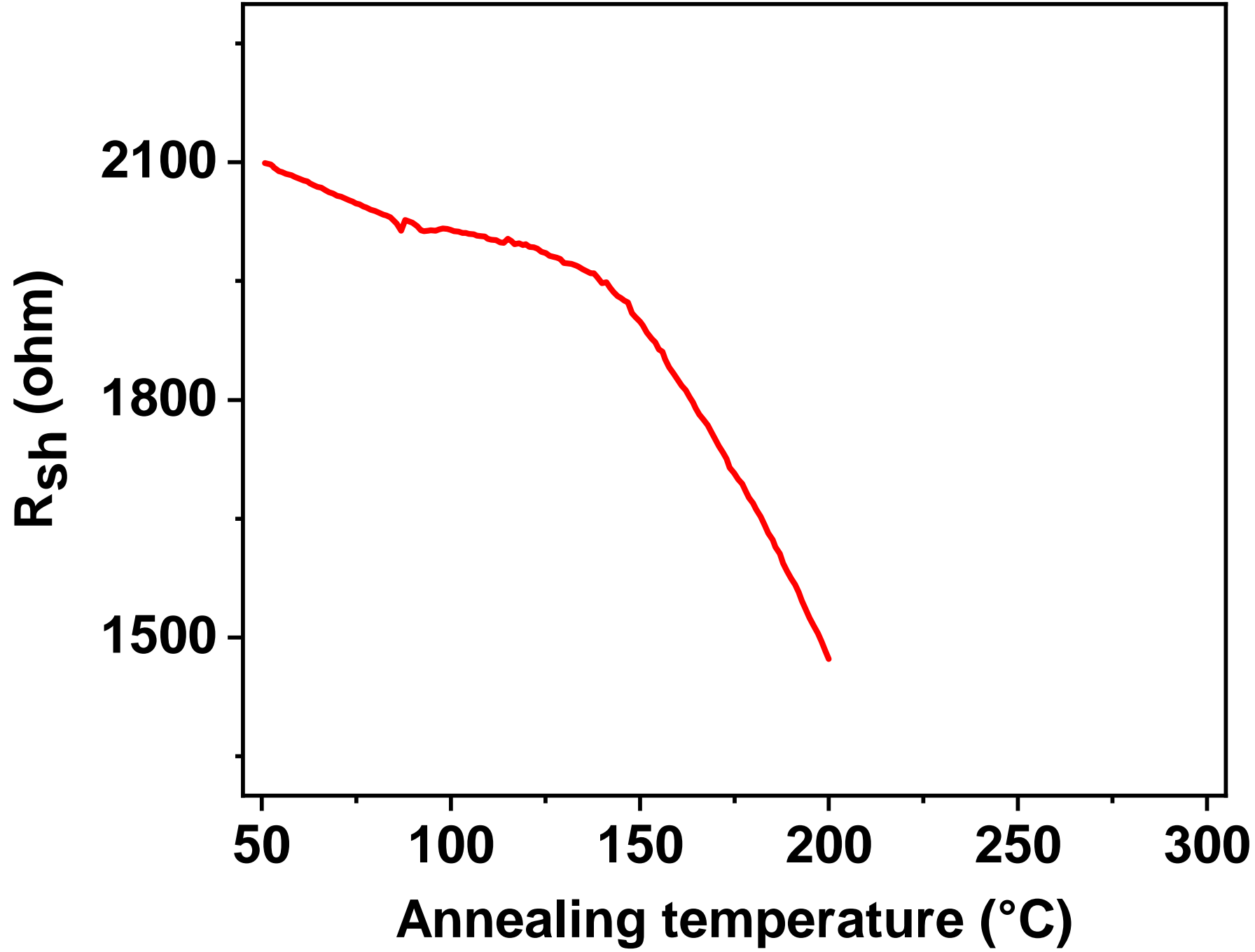

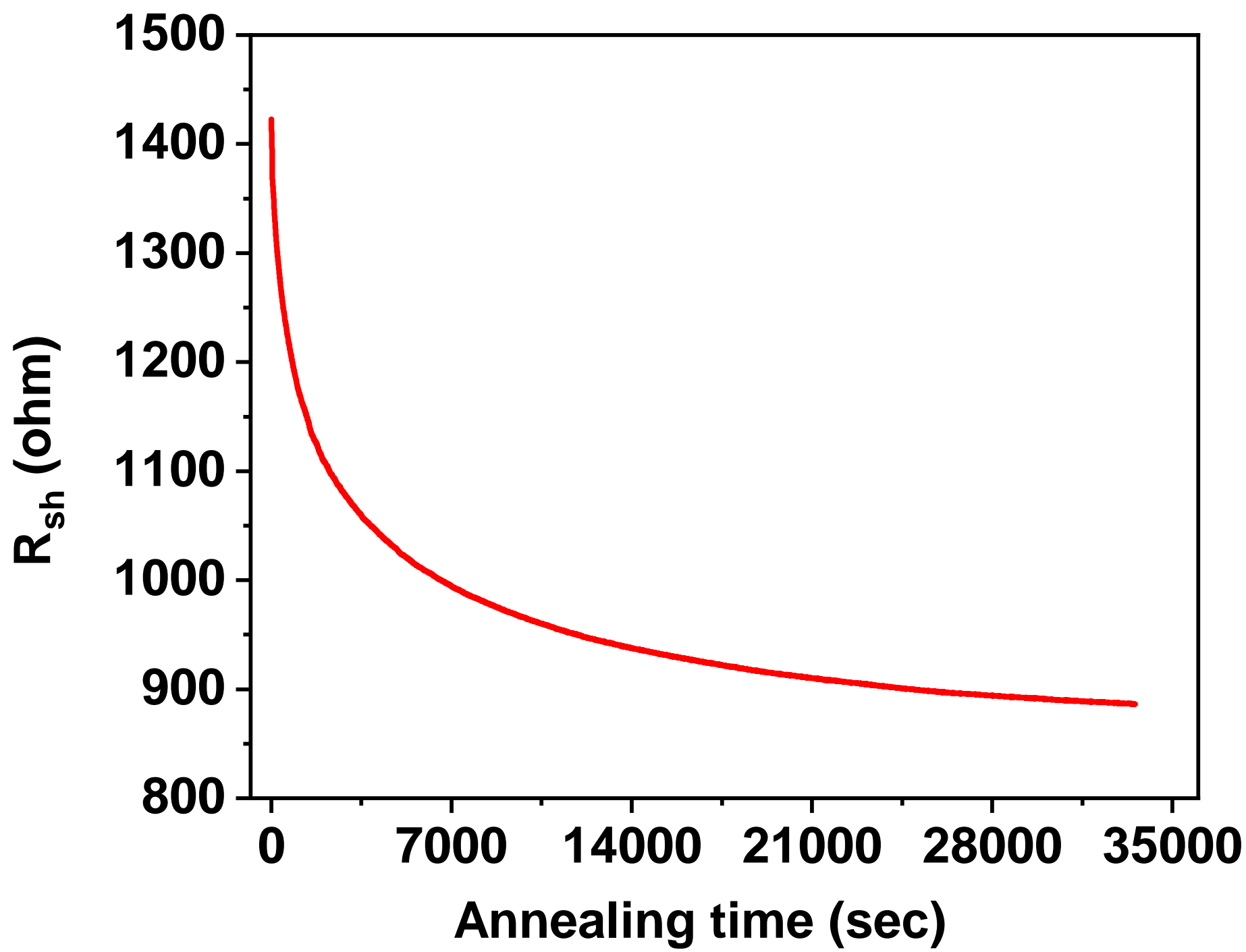

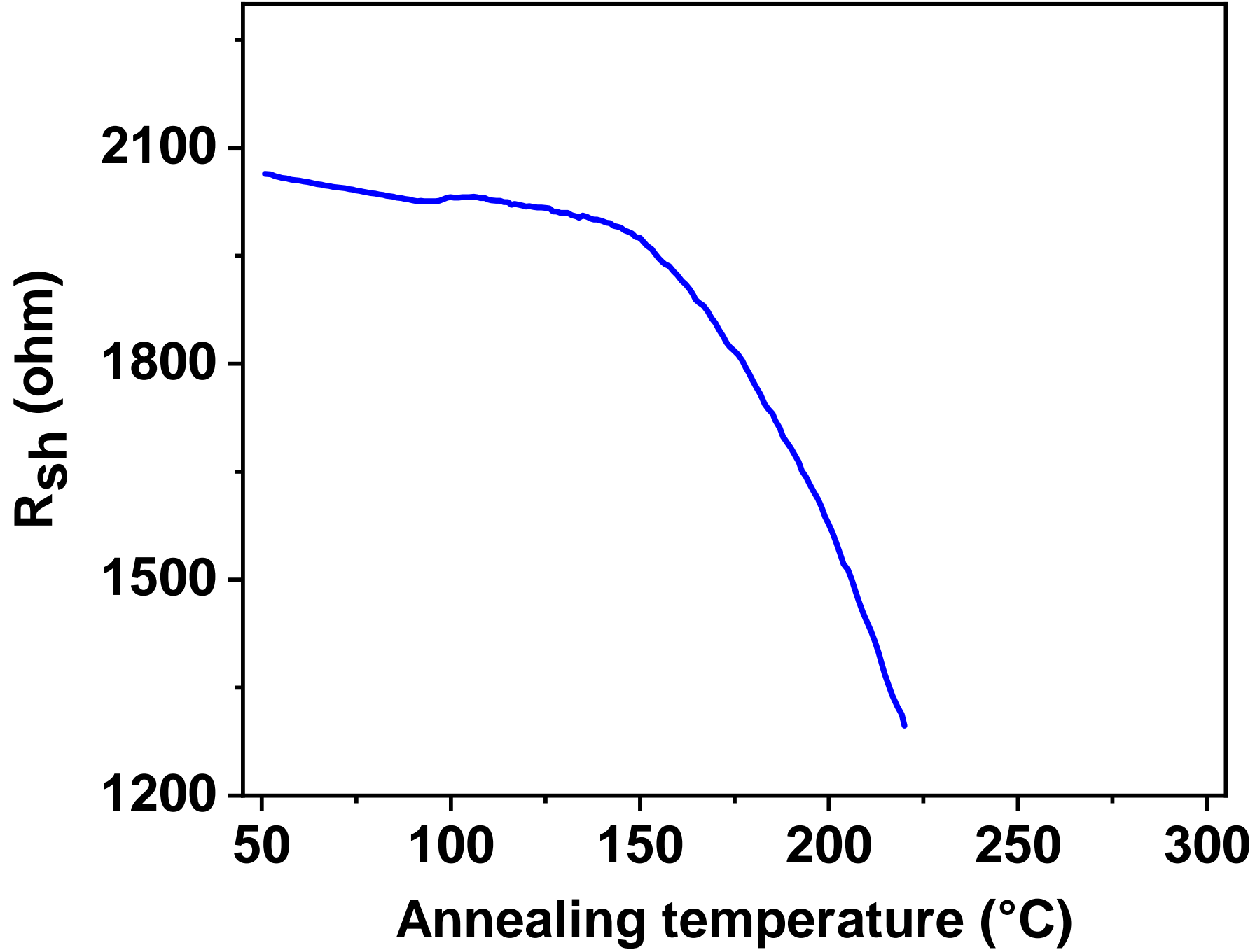

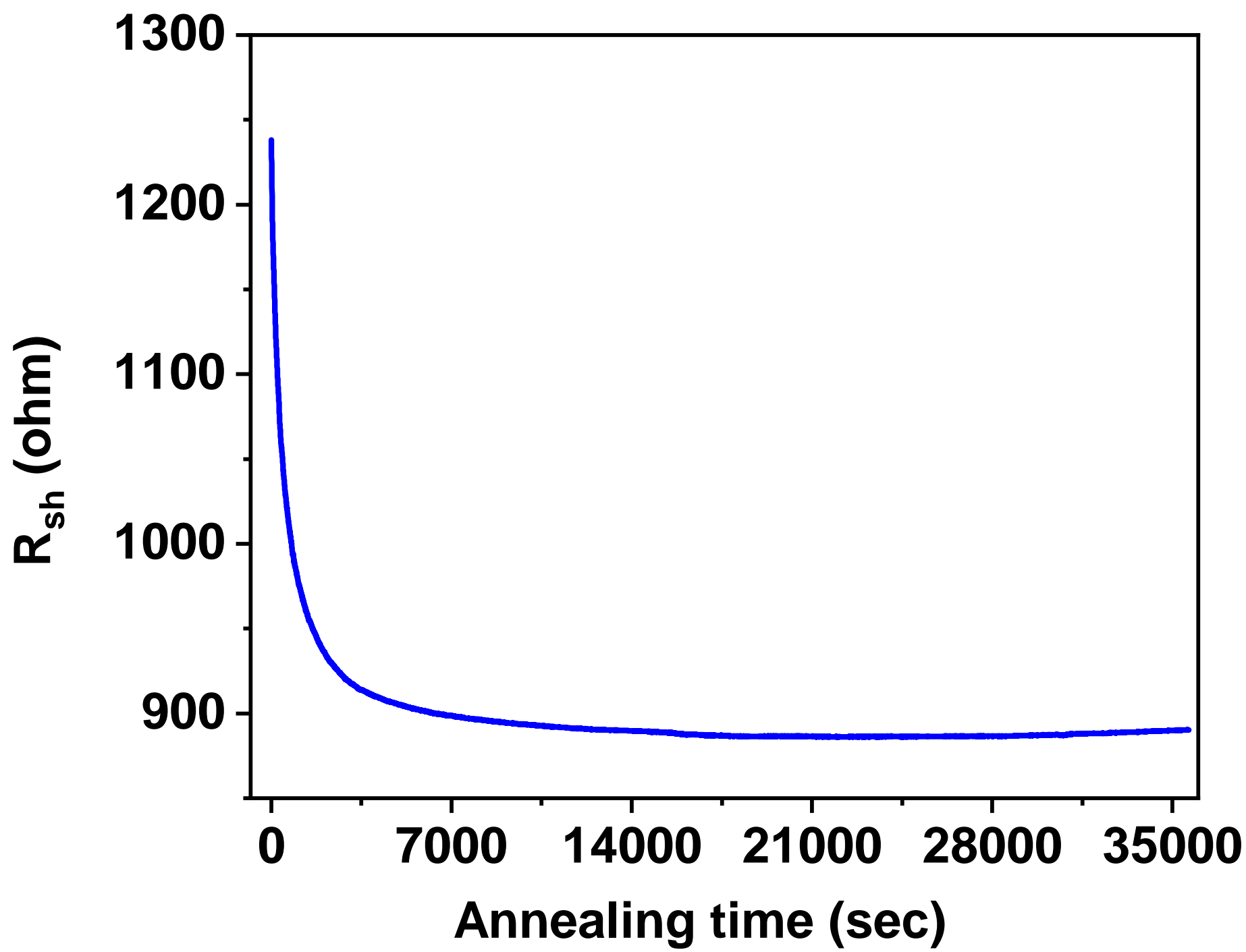

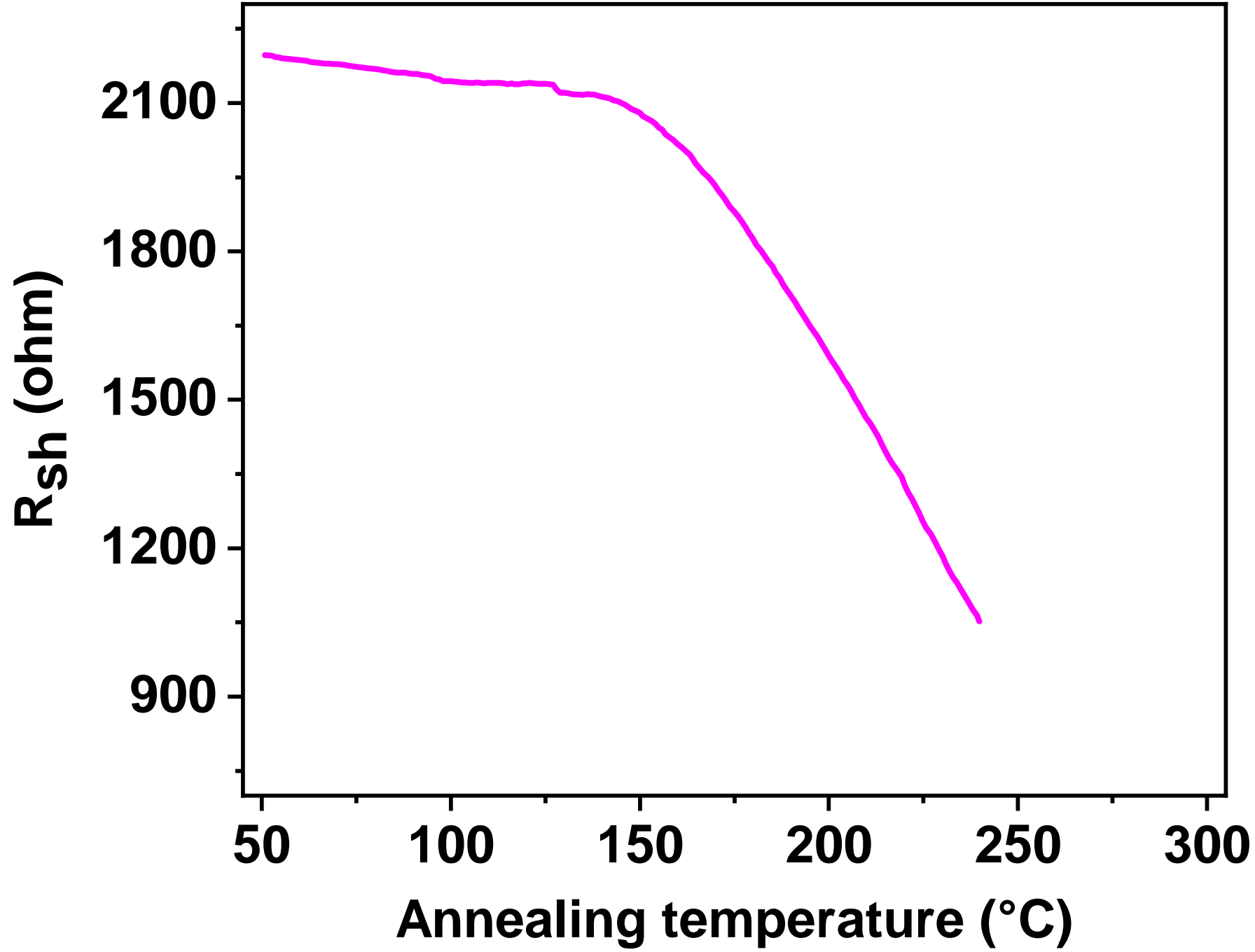

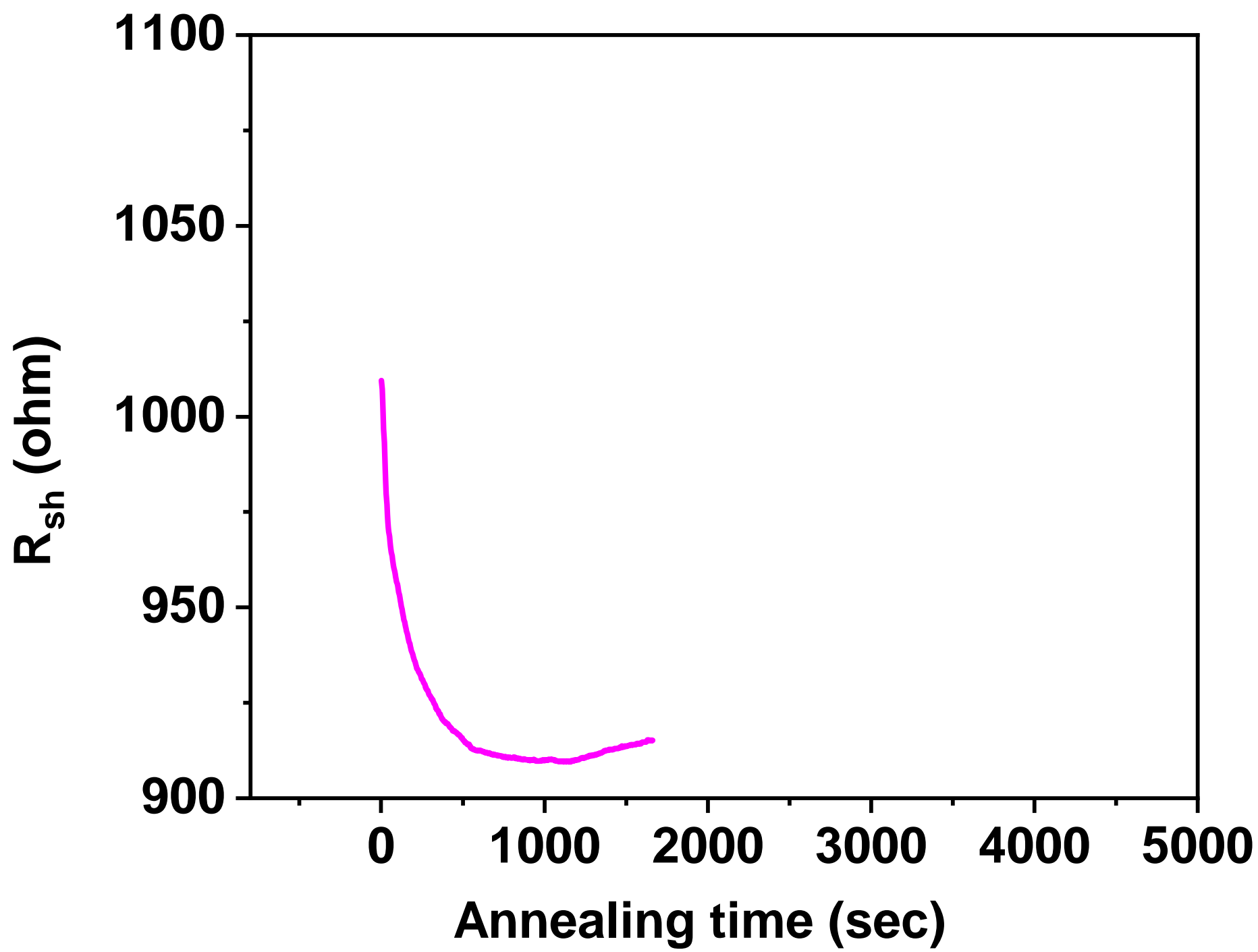

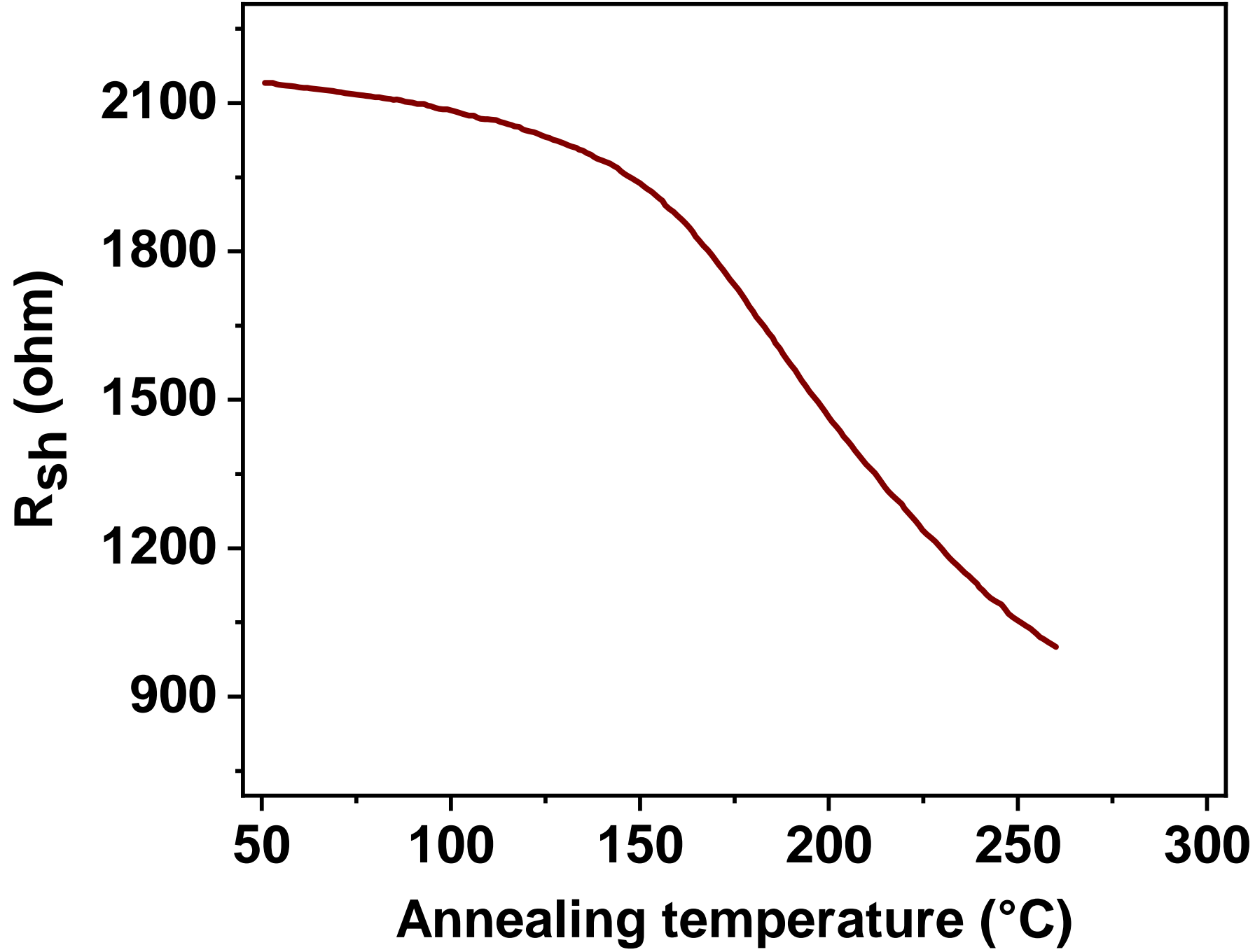

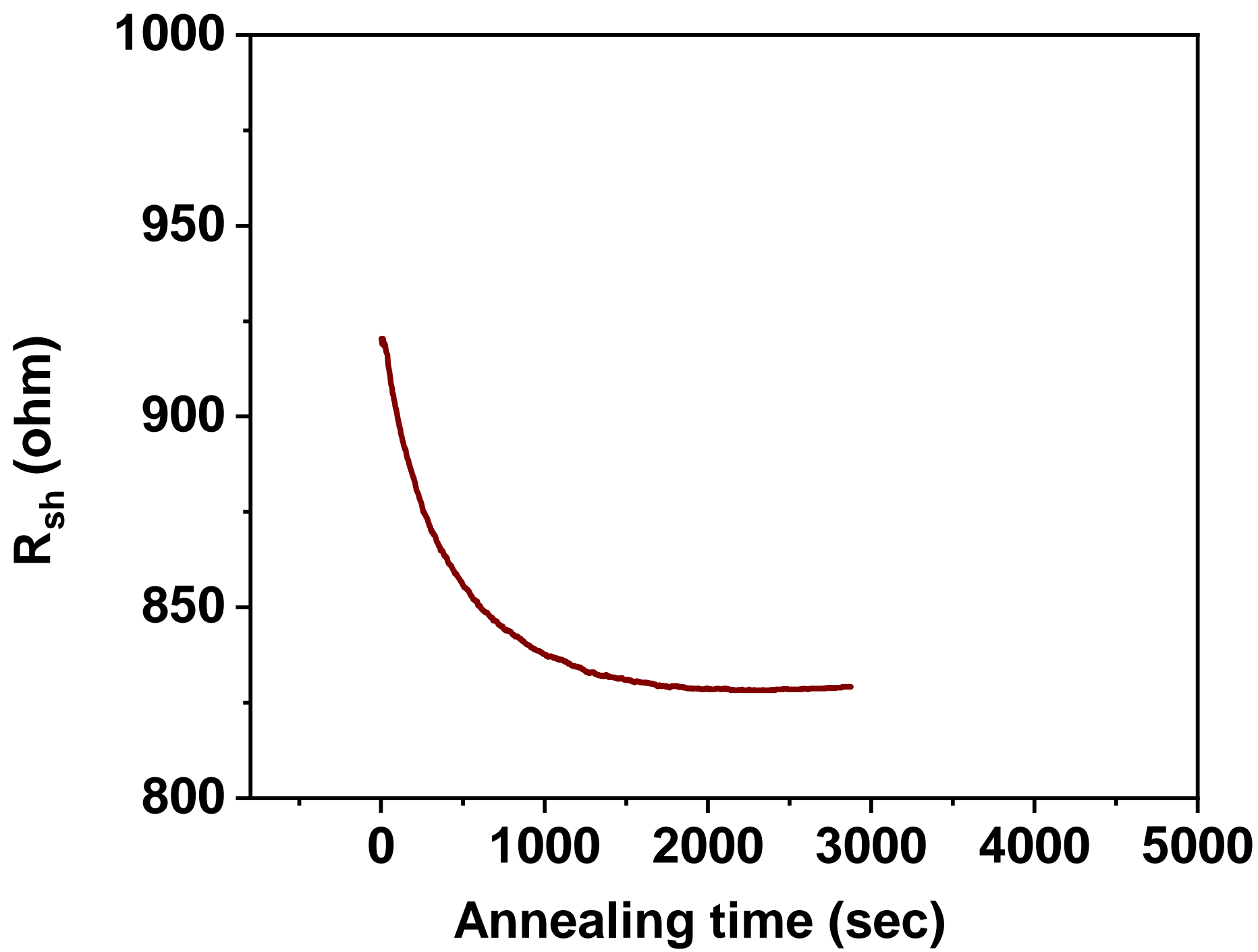

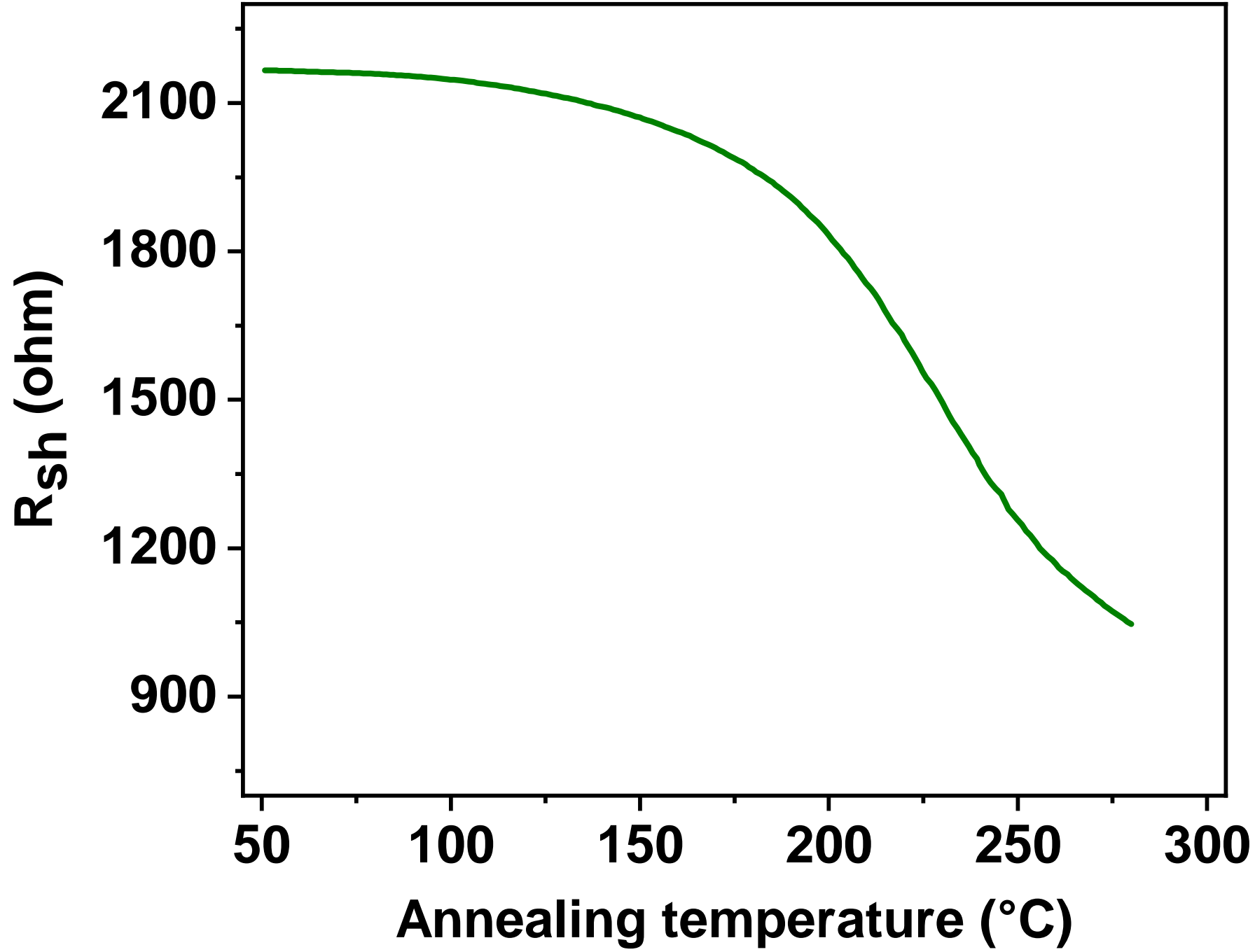

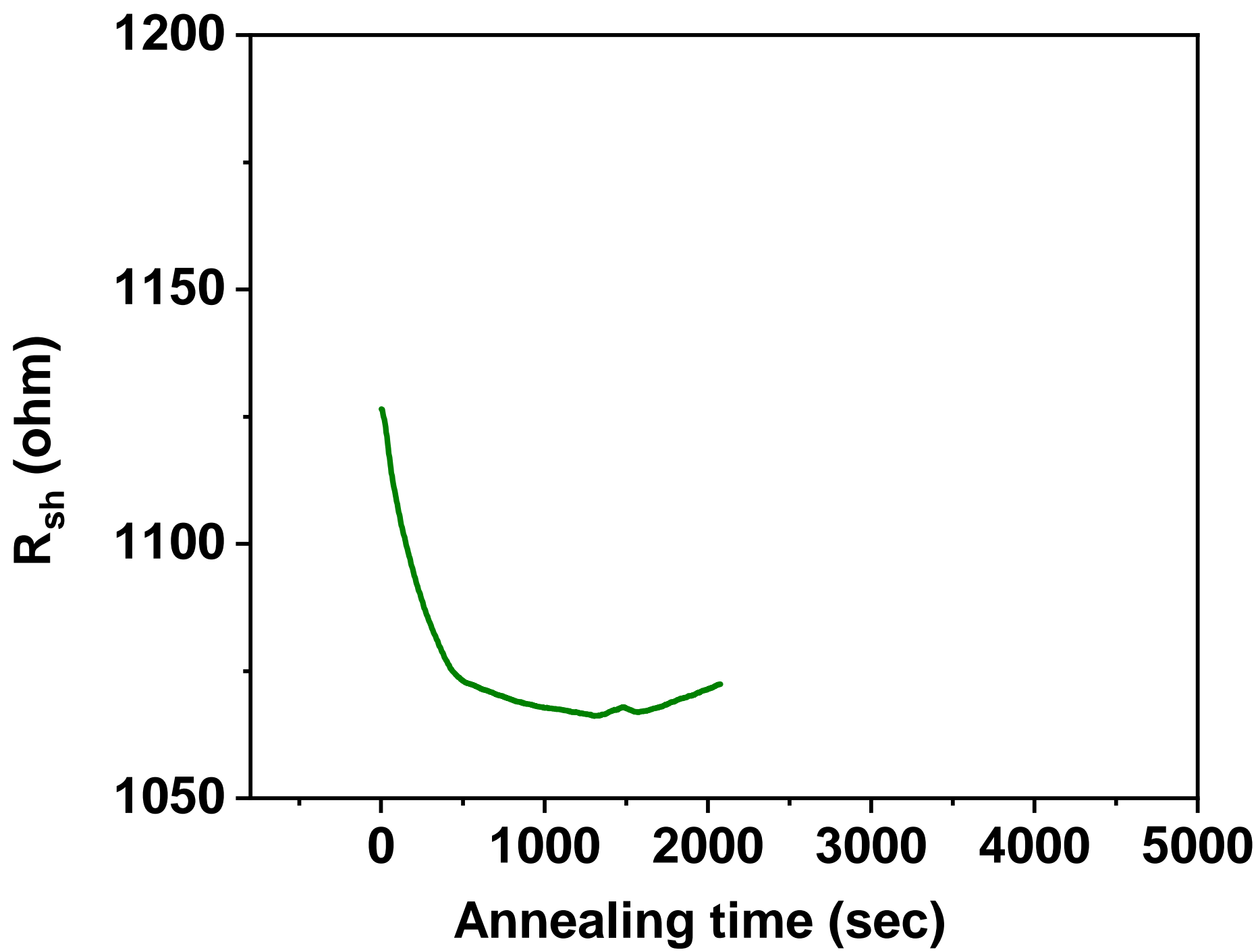

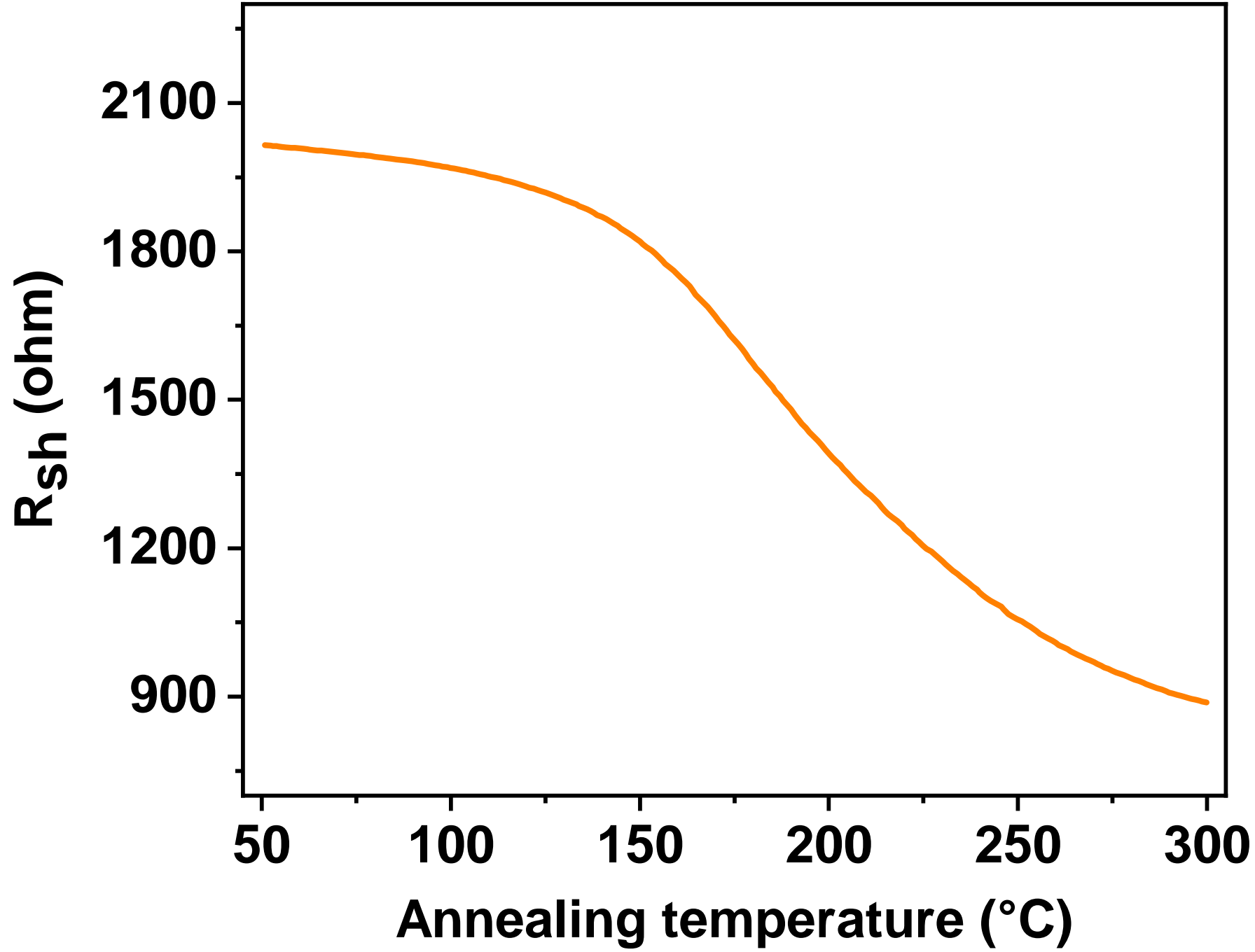

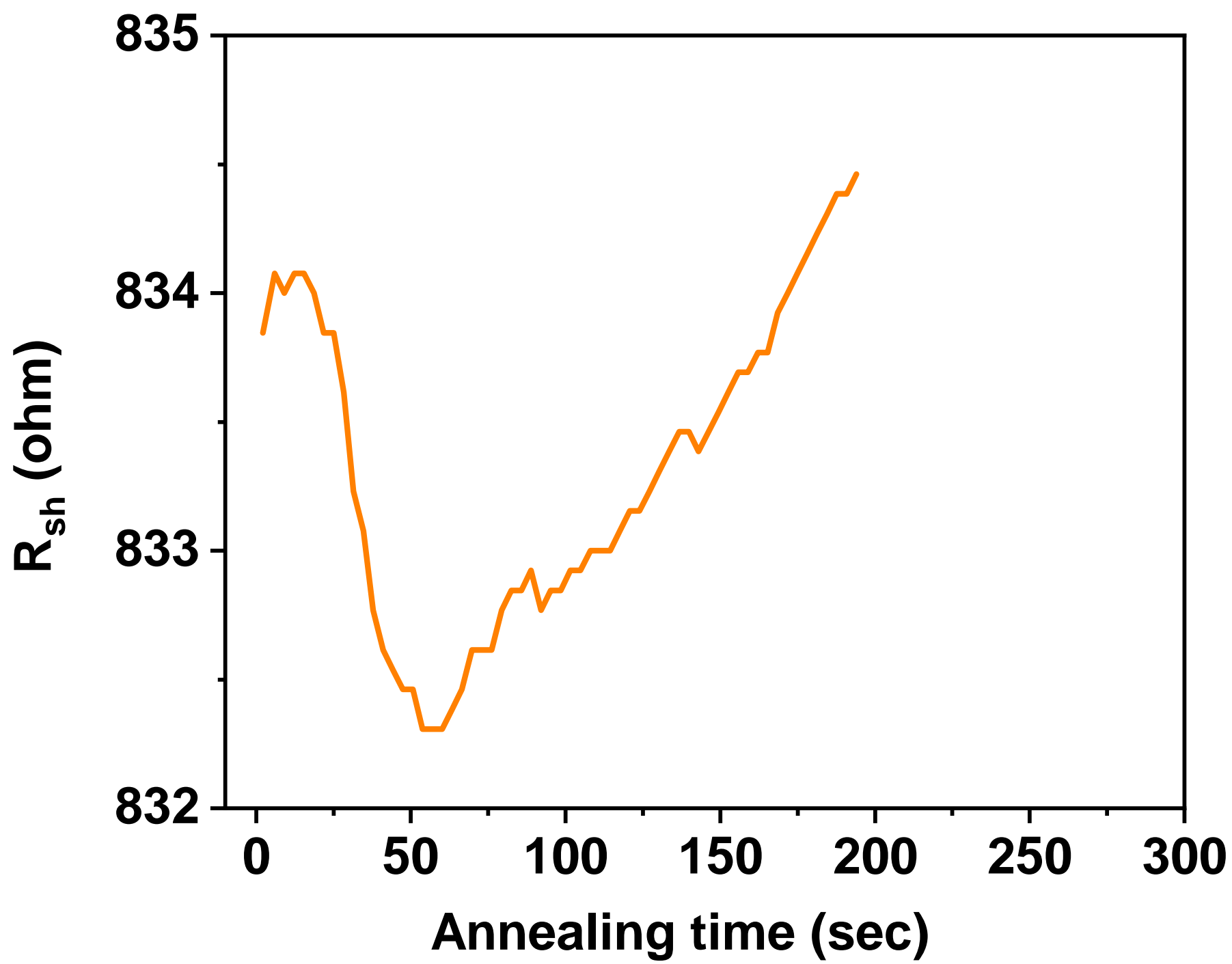

Supplement: Supplementary file 1 — ao4c01857_si_001.zip [file ao4c01857_si_001.zip › All Supporting Information files.pdf]

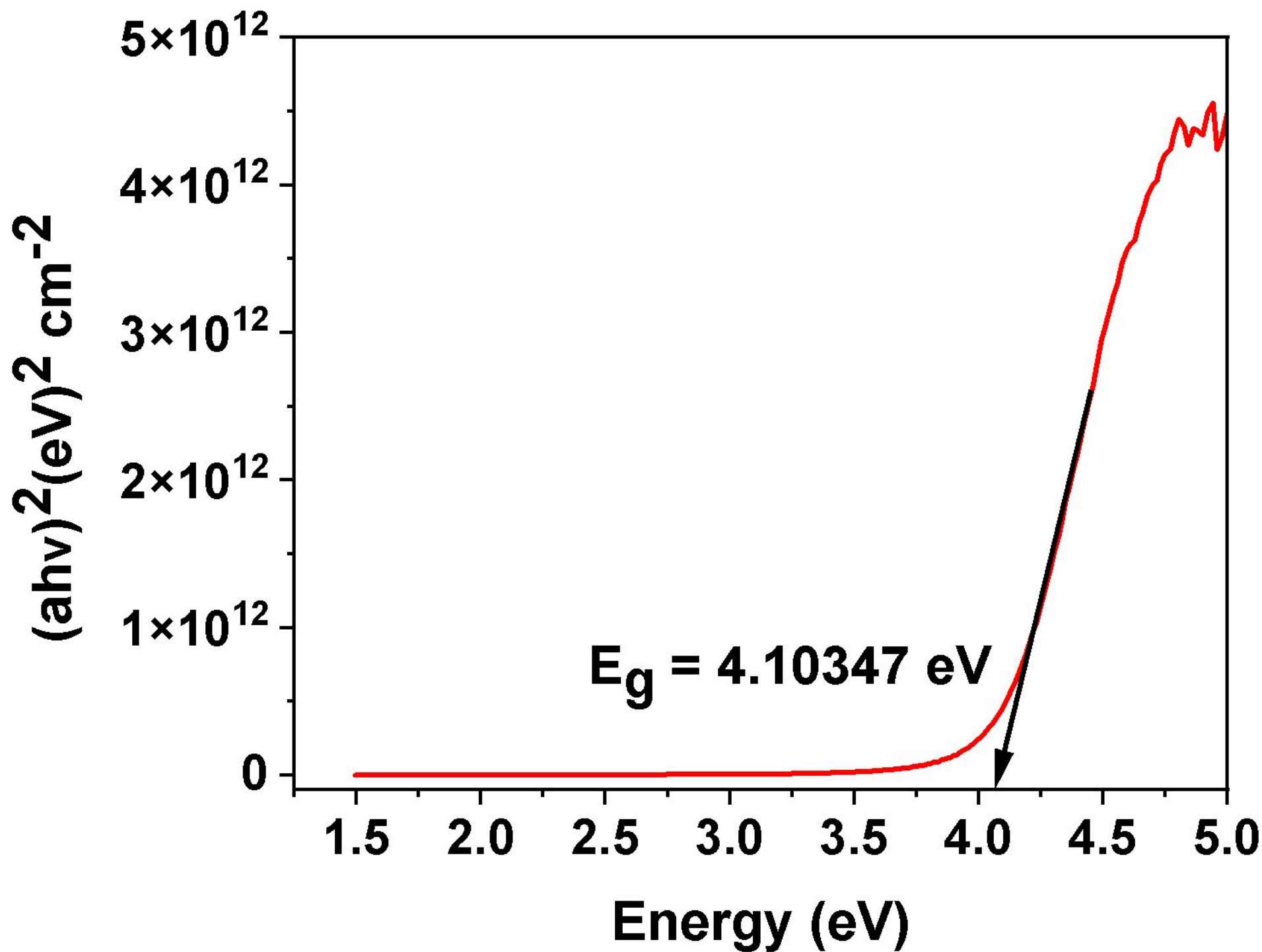

Supplement: Supplementary file 1 — ao4c01857_si_001.zip [file ao4c01857_si_001.zip › Figures/Bandgap-ZTO.pdf]

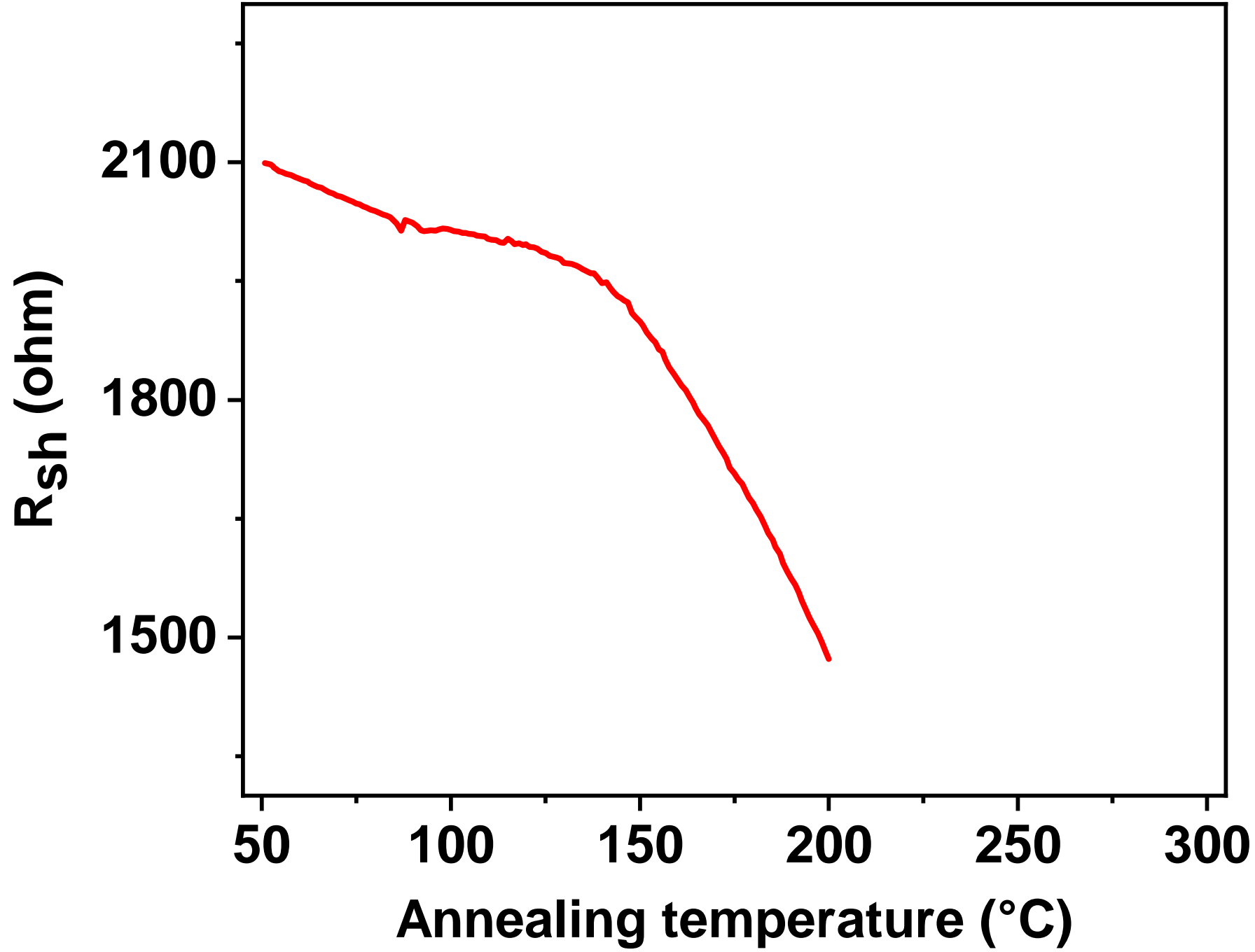

Supplement: Supplementary file 1 — ao4c01857_si_001.zip [file ao4c01857_si_001.zip › Figures/200C.pdf]

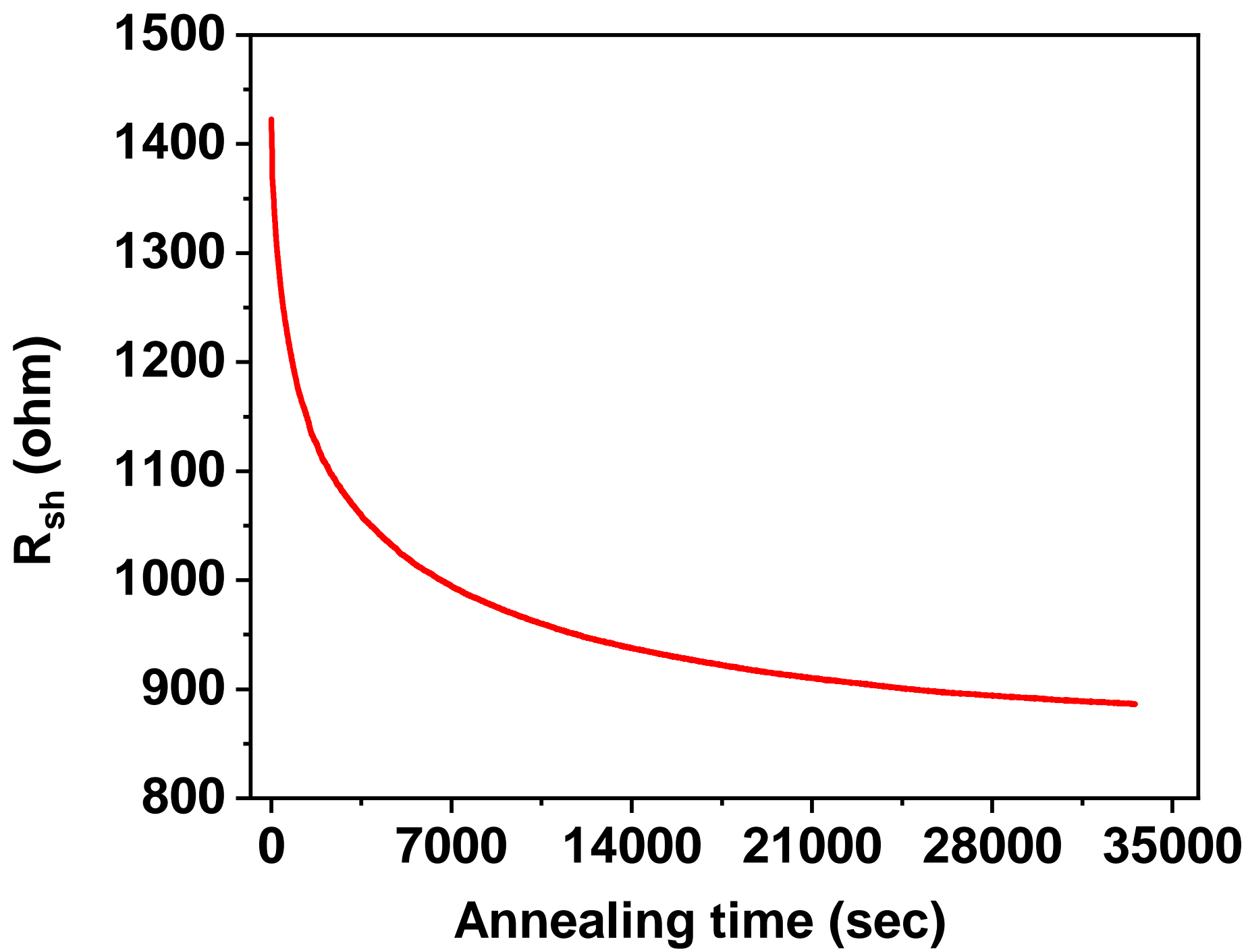

Supplement: Supplementary file 1 — ao4c01857_si_001.zip [file ao4c01857_si_001.zip › Figures/200C-stable.pdf]

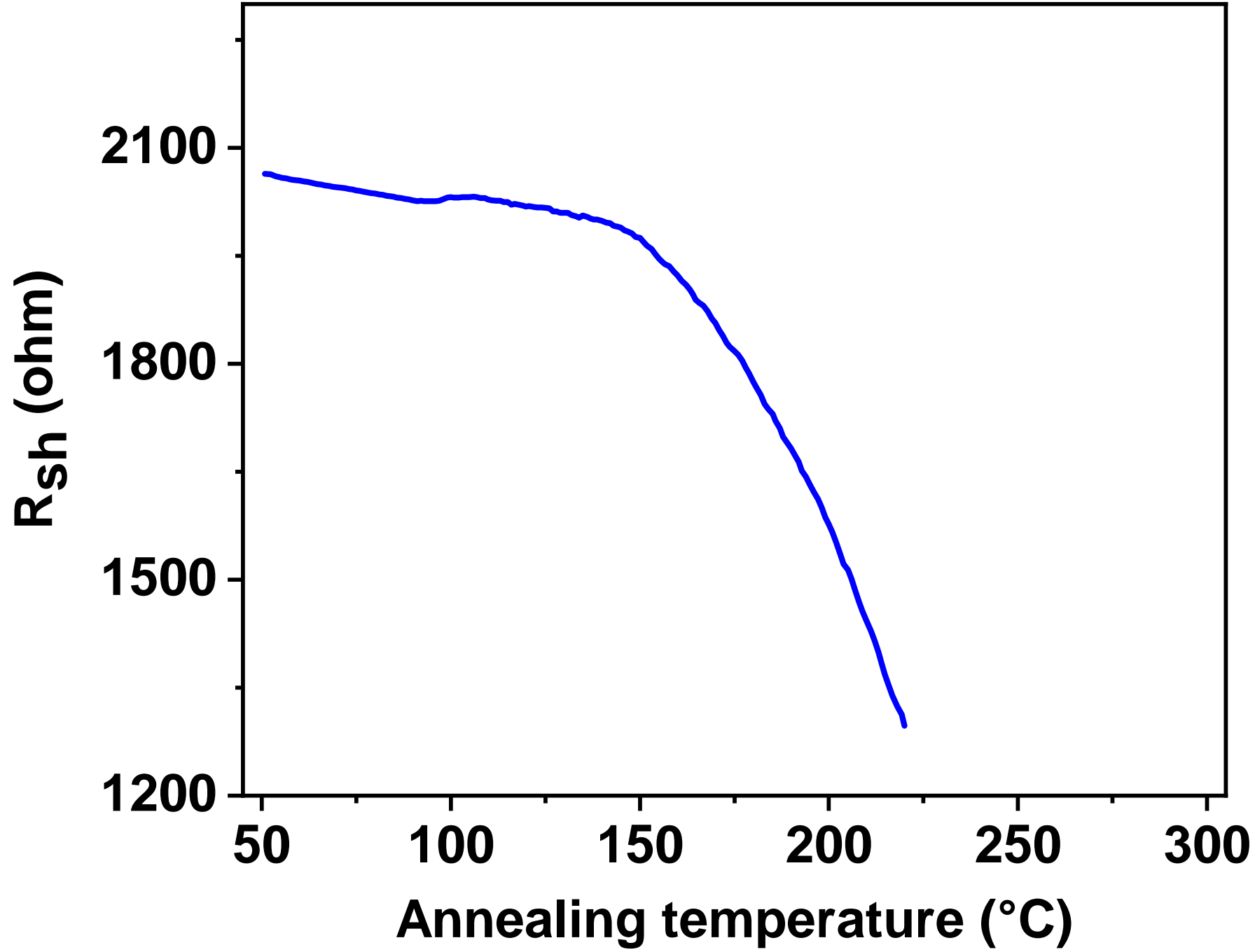

Supplement: Supplementary file 1 — ao4c01857_si_001.zip [file ao4c01857_si_001.zip › Figures/220C.pdf]

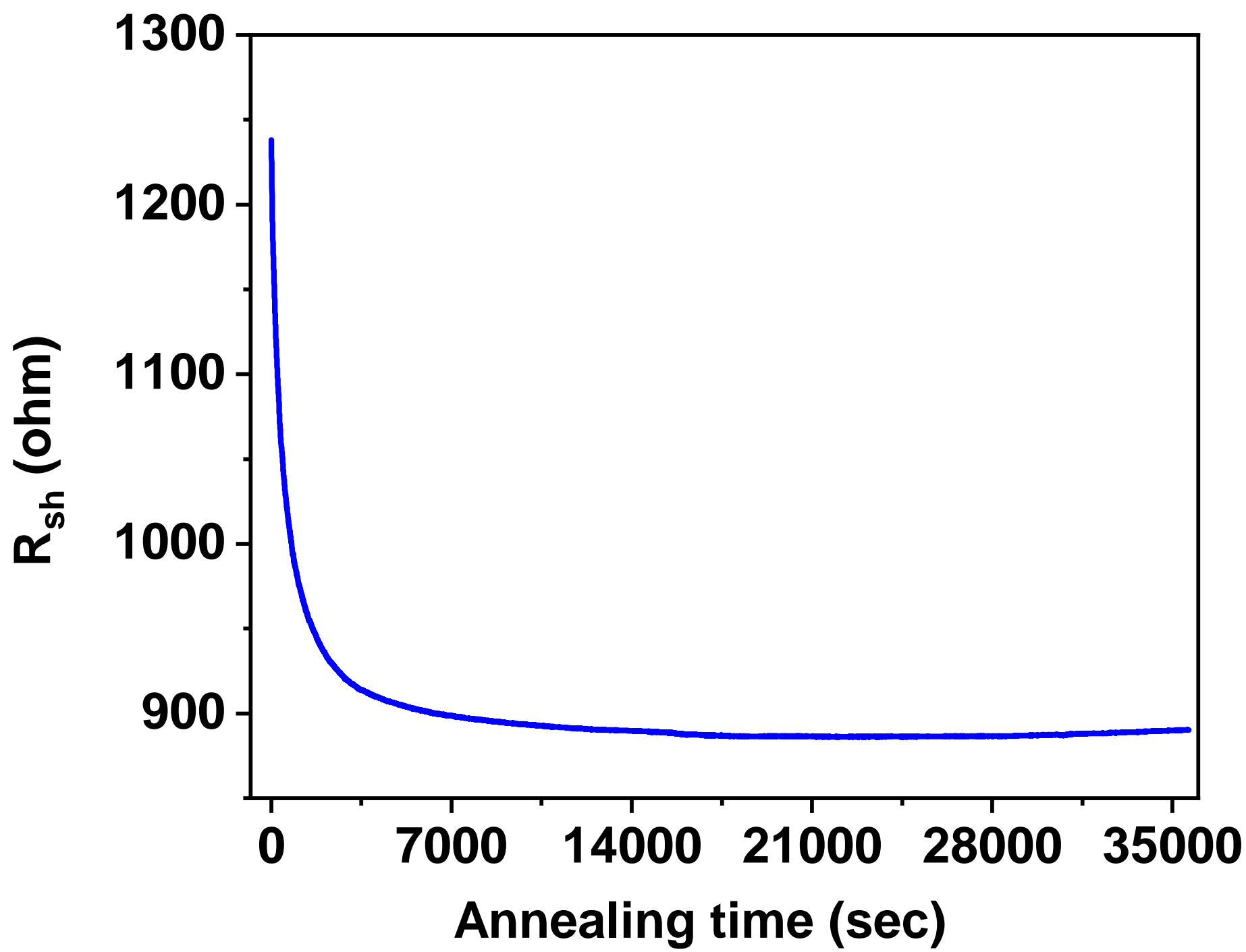

Supplement: Supplementary file 1 — ao4c01857_si_001.zip [file ao4c01857_si_001.zip › Figures/220C-stable.pdf]

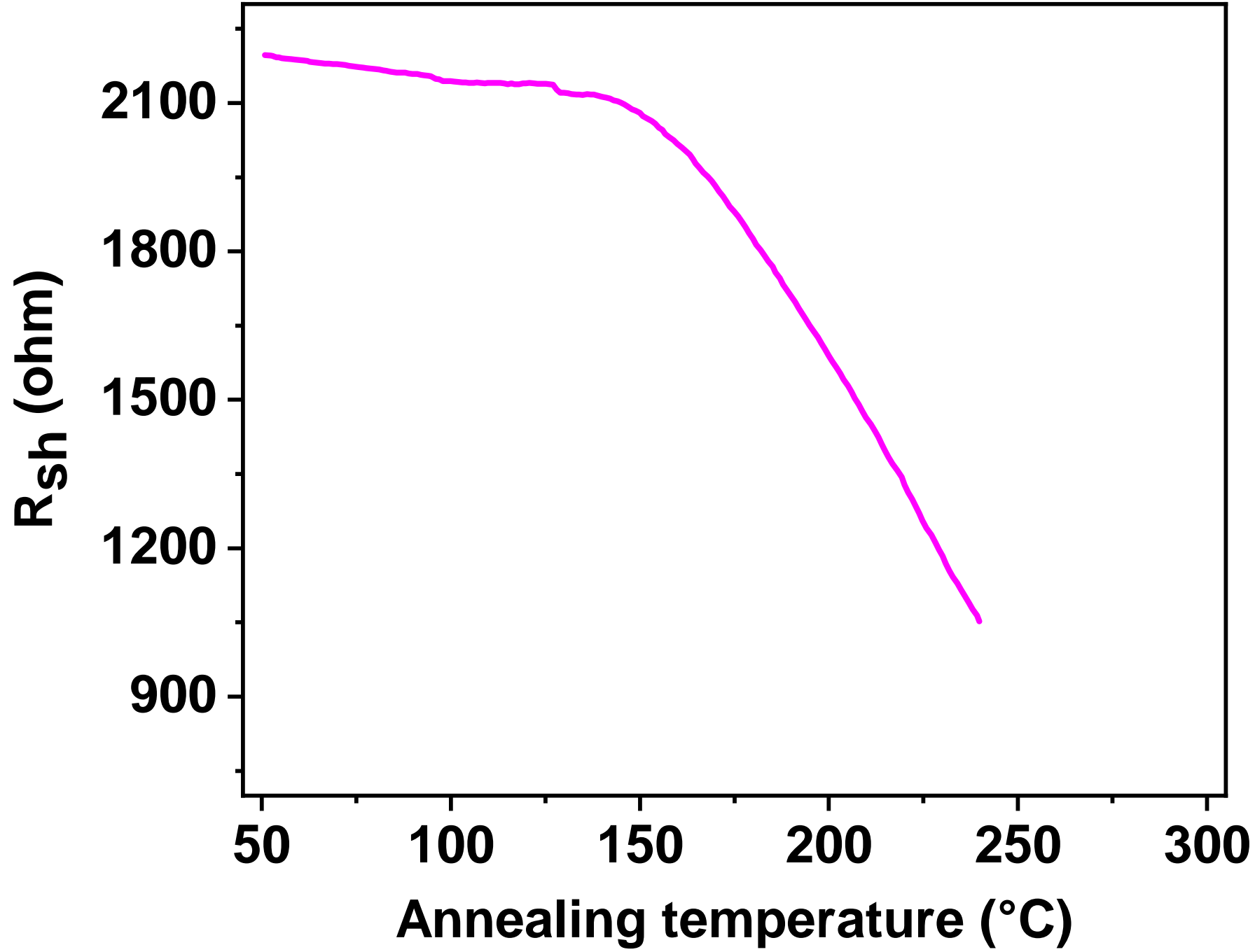

Supplement: Supplementary file 1 — ao4c01857_si_001.zip [file ao4c01857_si_001.zip › Figures/240C.pdf]

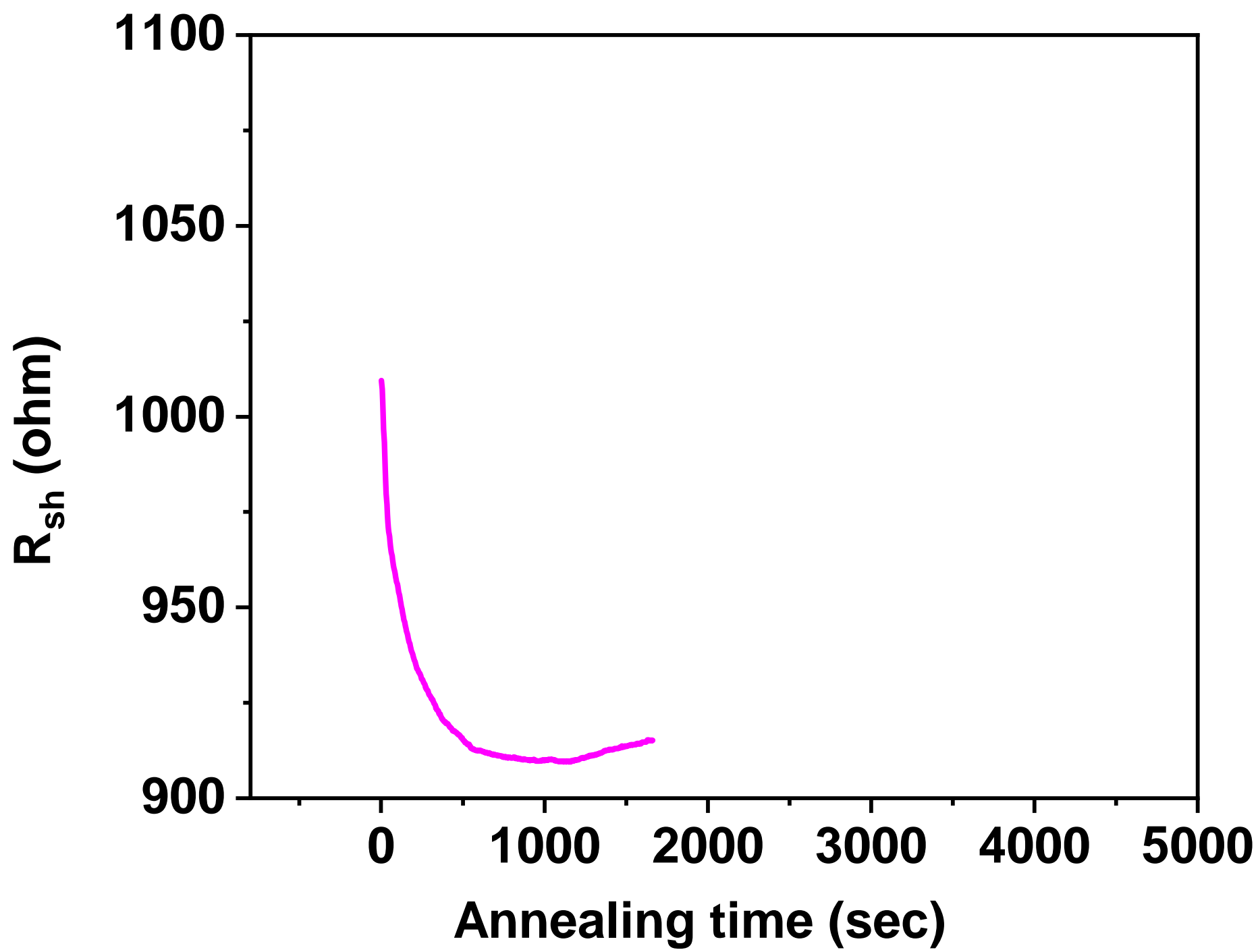

Supplement: Supplementary file 1 — ao4c01857_si_001.zip [file ao4c01857_si_001.zip › Figures/240C-stable.pdf]

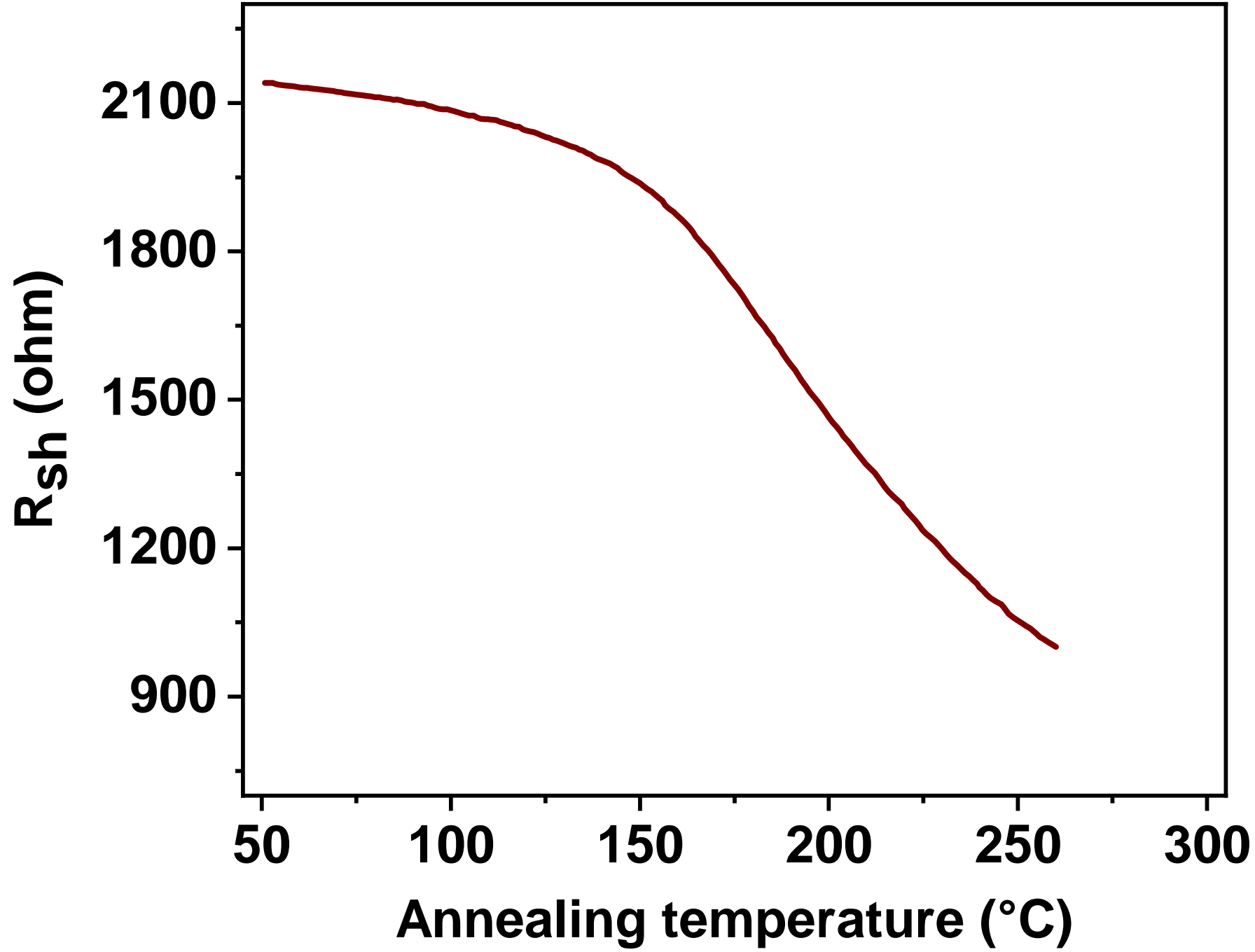

Supplement: Supplementary file 1 — ao4c01857_si_001.zip [file ao4c01857_si_001.zip › Figures/260C.pdf]

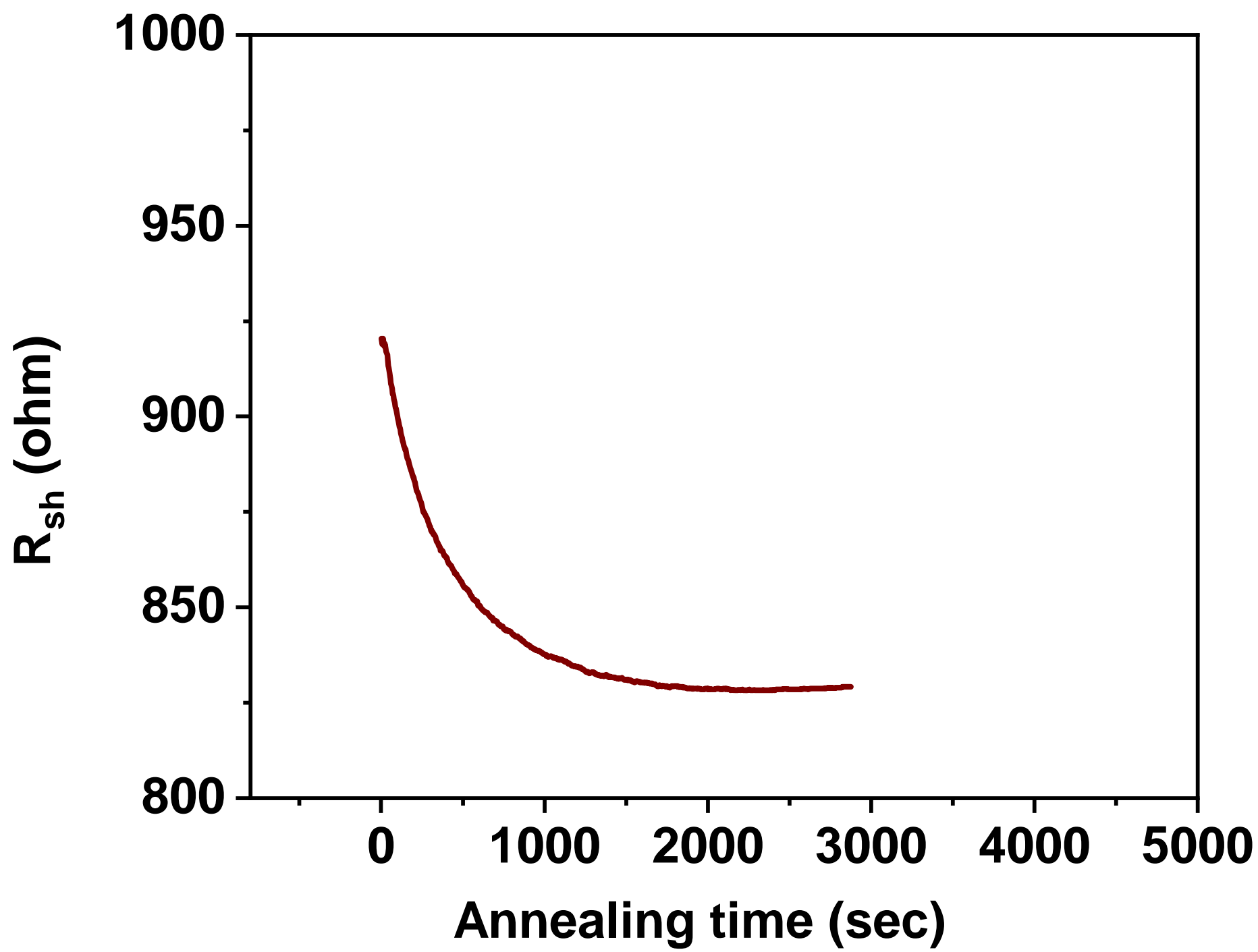

Supplement: Supplementary file 1 — ao4c01857_si_001.zip [file ao4c01857_si_001.zip › Figures/260C-stable.pdf]

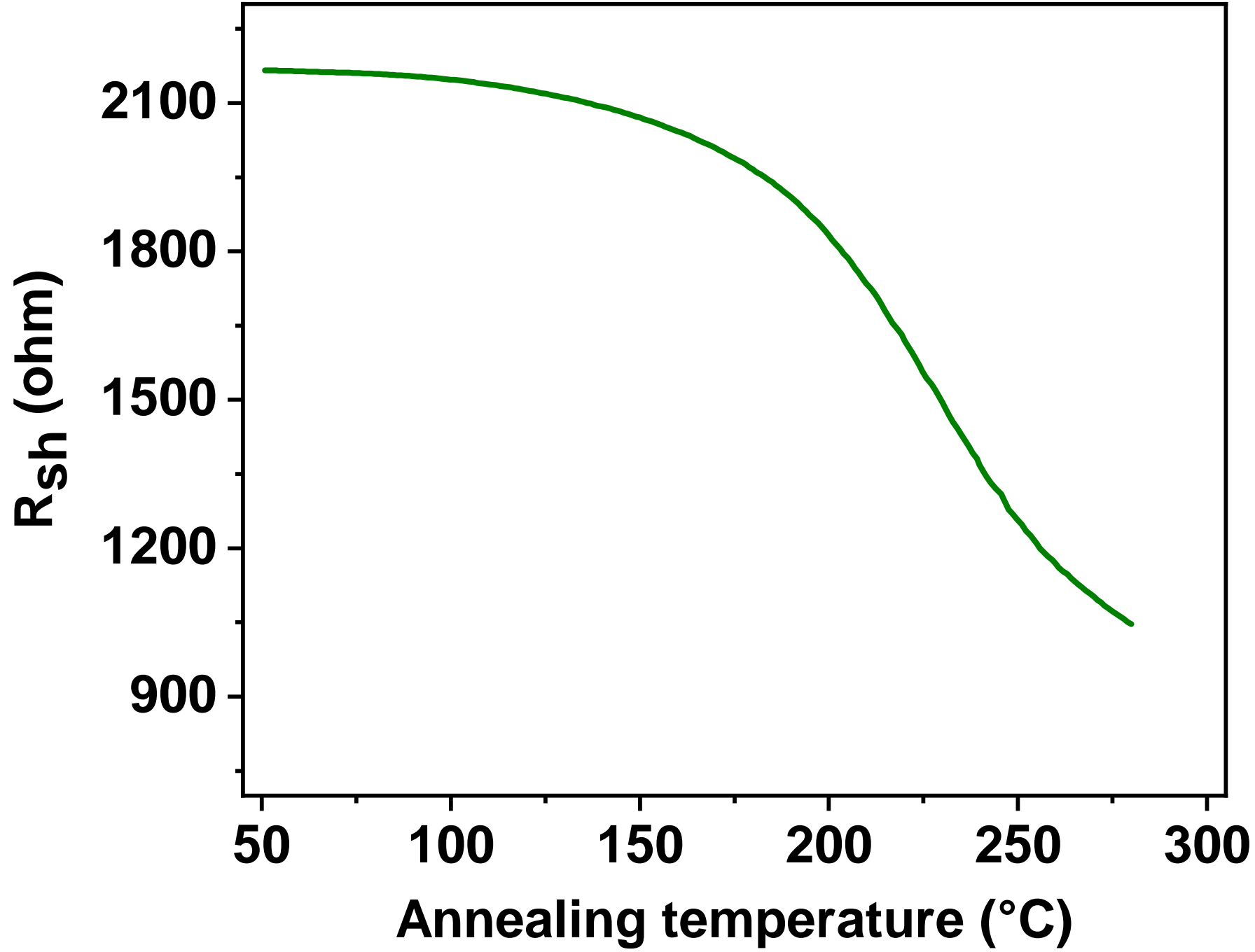

Supplement: Supplementary file 1 — ao4c01857_si_001.zip [file ao4c01857_si_001.zip › Figures/280C.pdf]

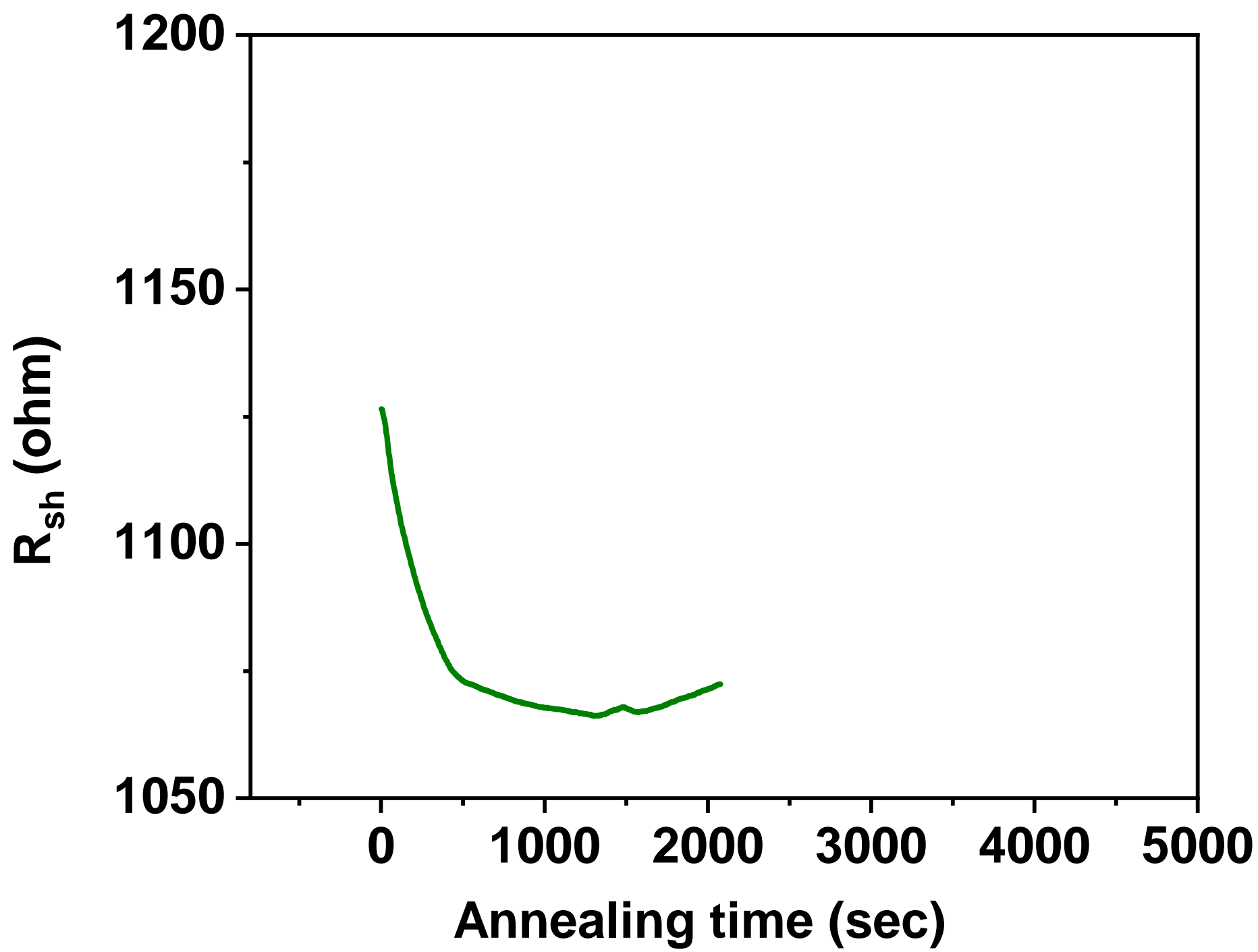

Supplement: Supplementary file 1 — ao4c01857_si_001.zip [file ao4c01857_si_001.zip › Figures/280C-stable.pdf]

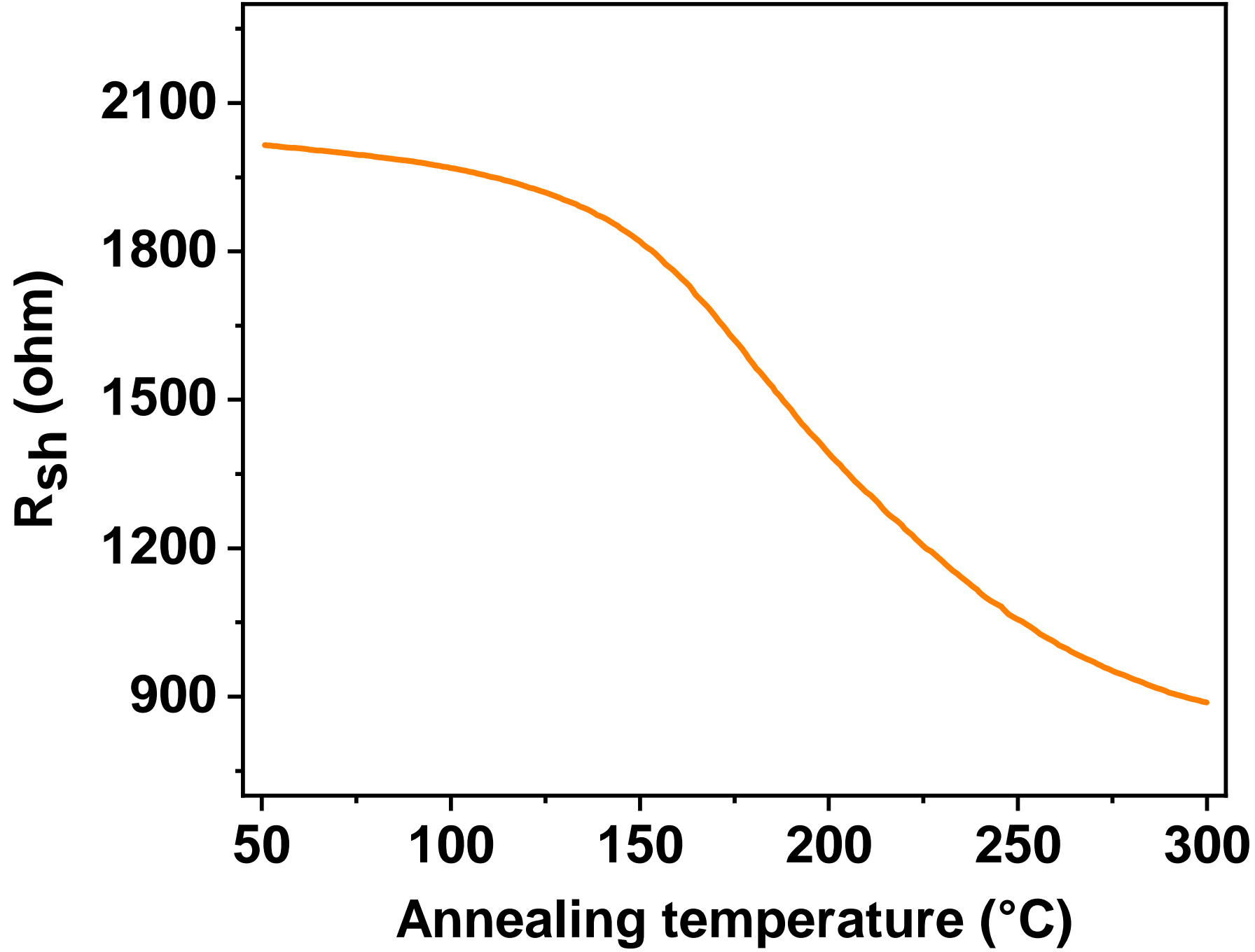

Supplement: Supplementary file 1 — ao4c01857_si_001.zip [file ao4c01857_si_001.zip › Figures/300C.pdf]

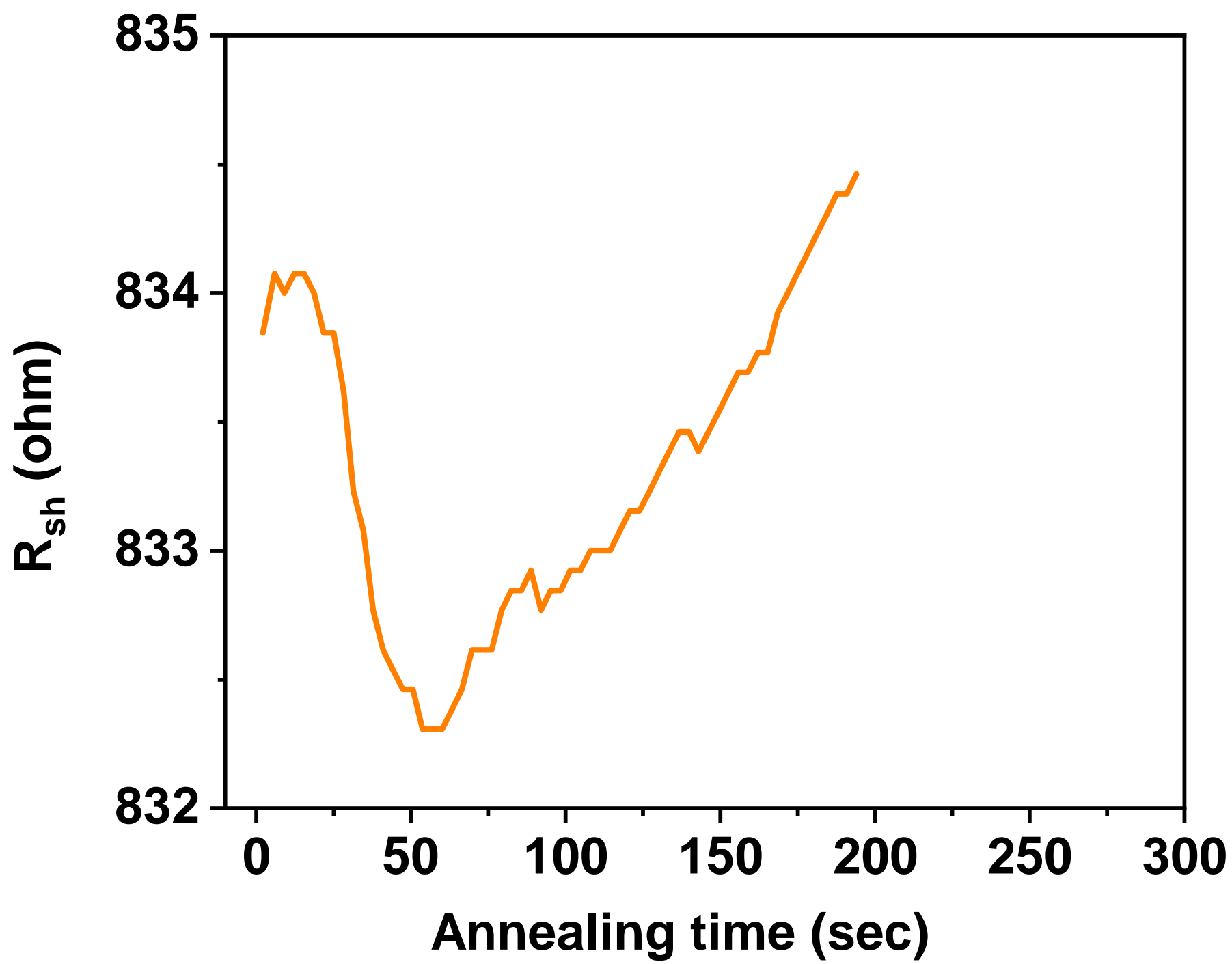

Supplement: Supplementary file 1 — ao4c01857_si_001.zip [file ao4c01857_si_001.zip › Figures/300C-stable.pdf]

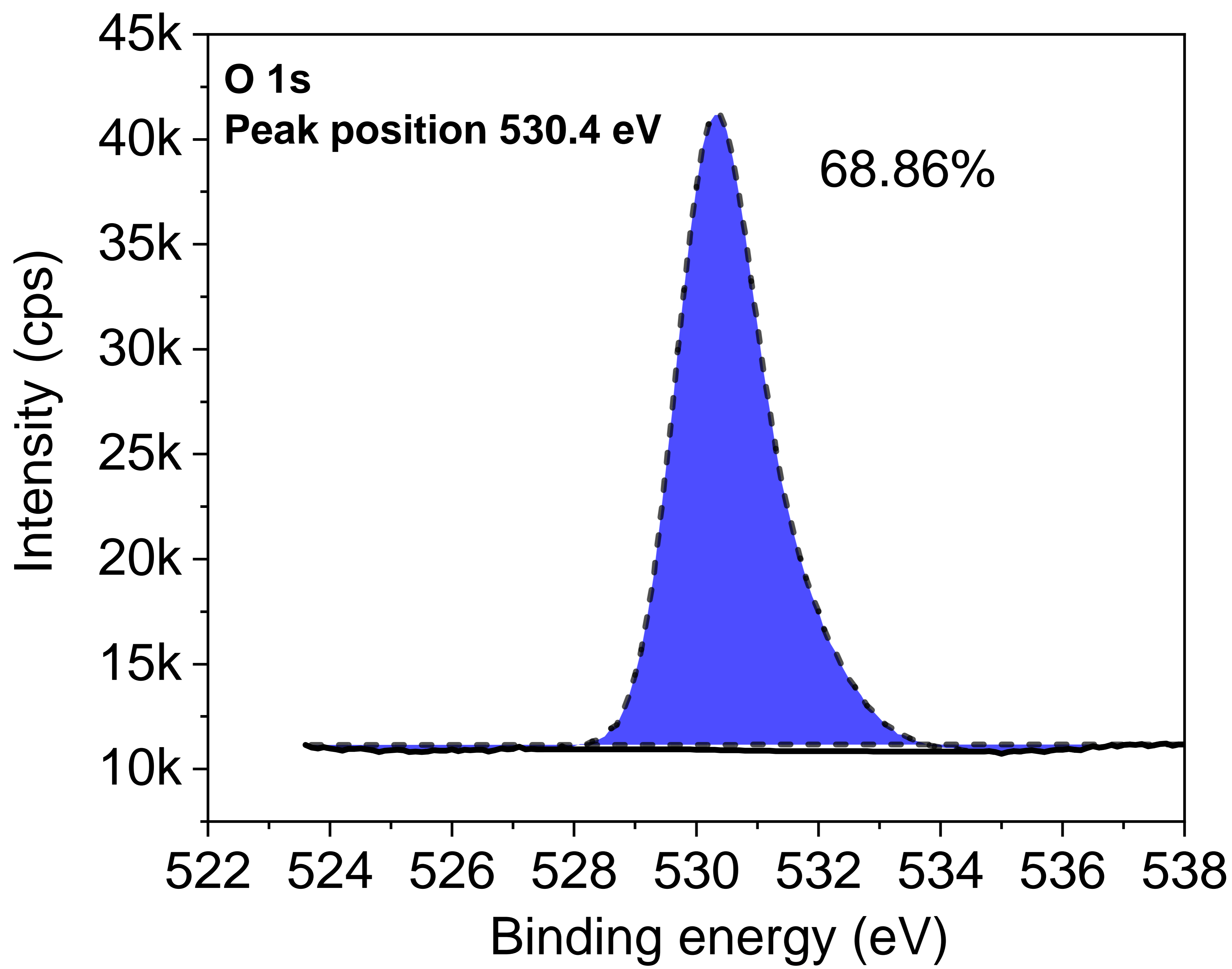

Supplement: Supplementary file 1 — ao4c01857_si_001.zip [file ao4c01857_si_001.zip › Figures/Oxygen-XPS-300c.pdf]

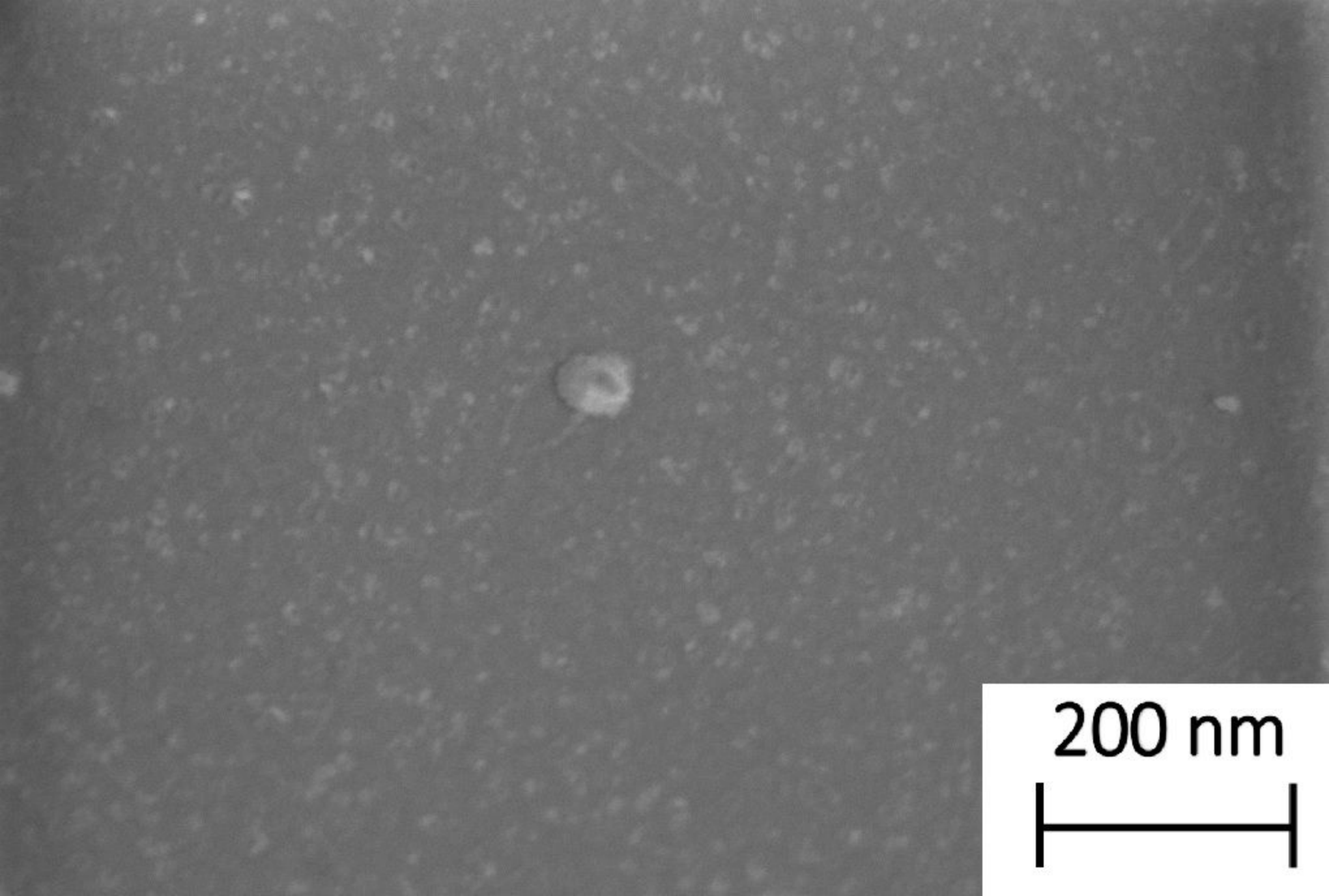

200 nm

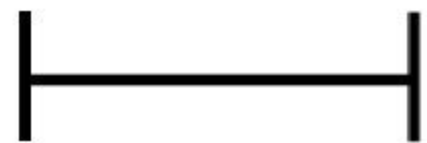

Supplement: Supplementary file 1 — ao4c01857_si_001.zip [file ao4c01857_si_001.zip › Figures/SEM-300c.pdf]

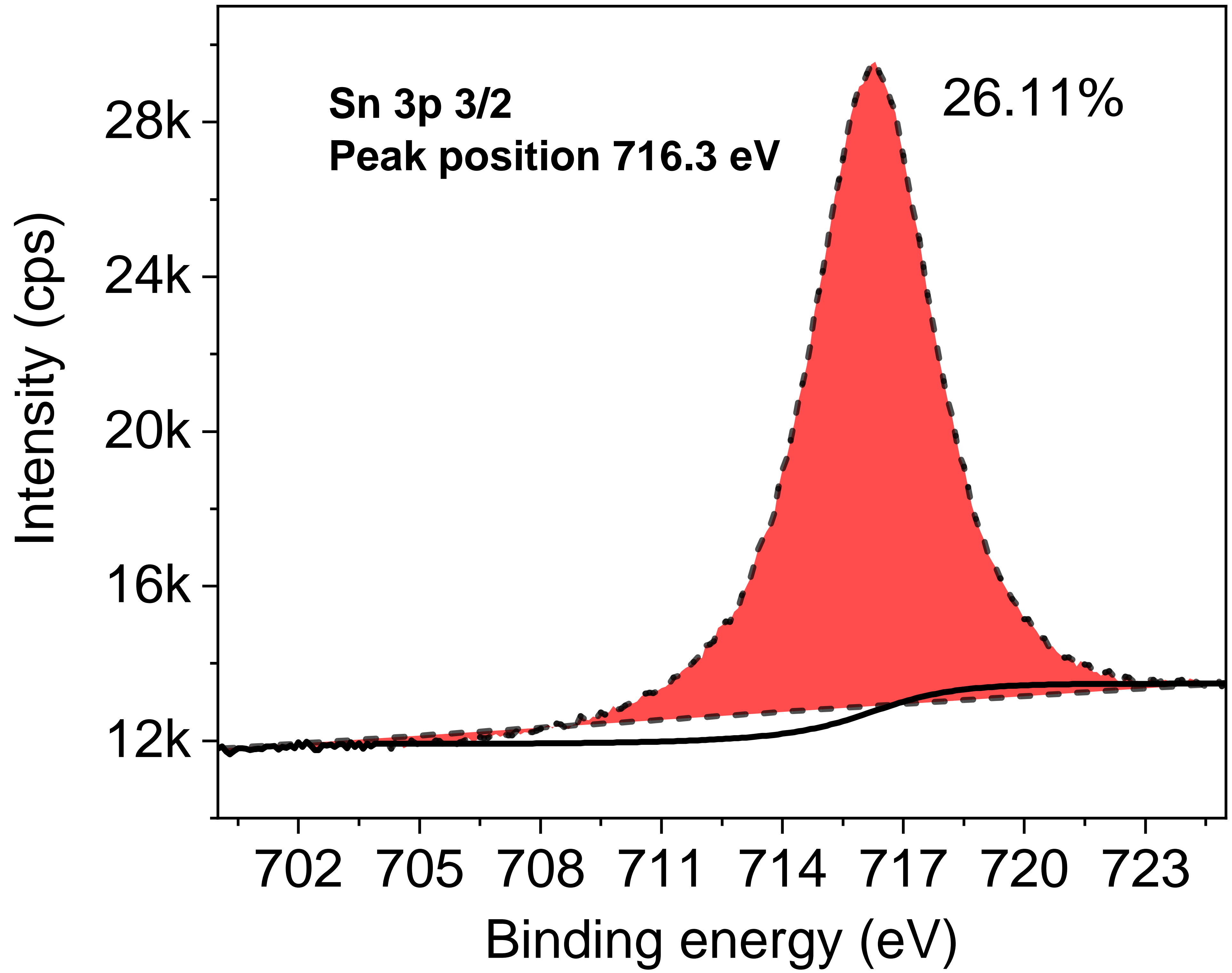

Supplement: Supplementary file 1 — ao4c01857_si_001.zip [file ao4c01857_si_001.zip › Figures/Tin-XPS-300c.pdf]

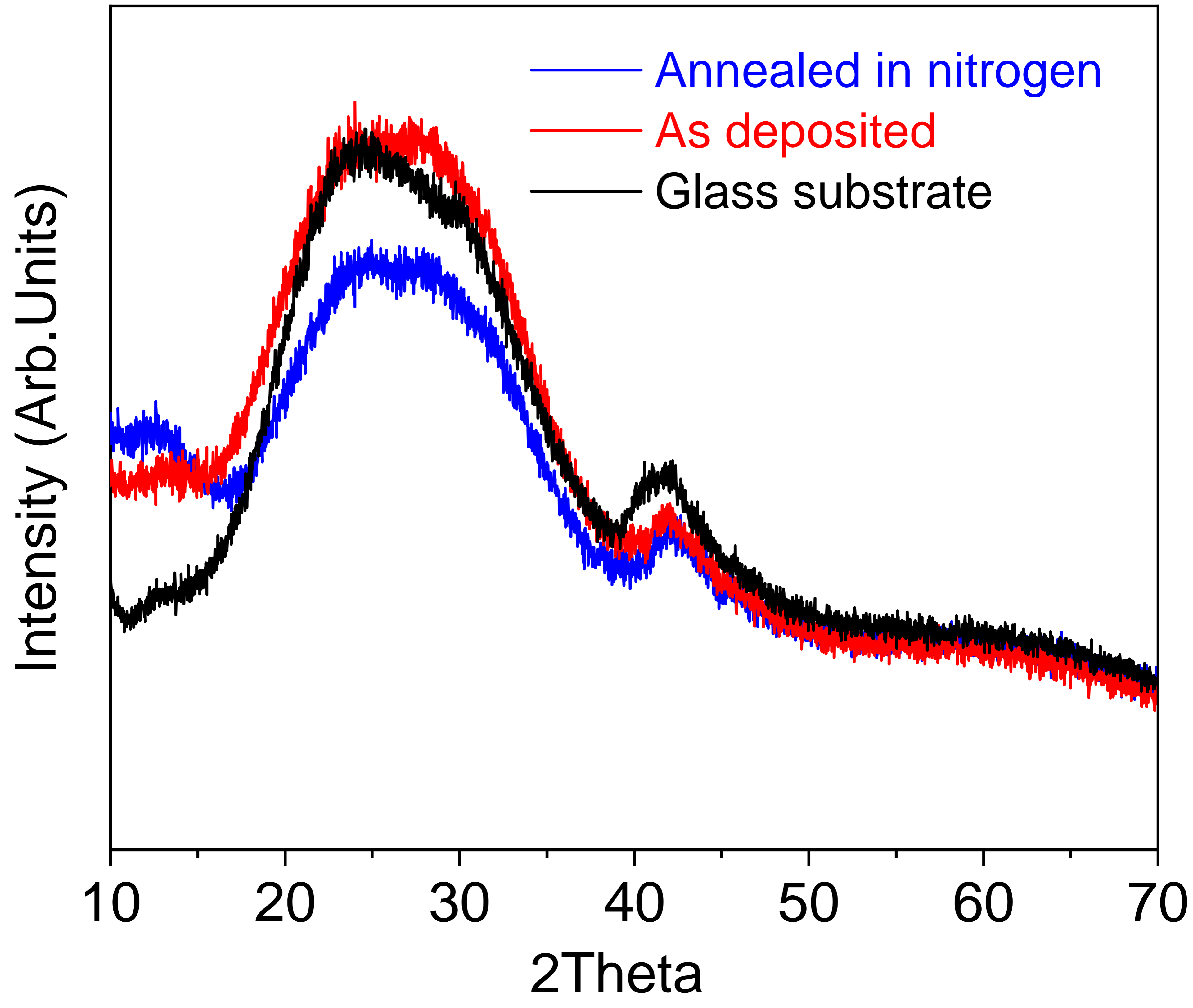

Supplement: Supplementary file 1 — ao4c01857_si_001.zip [file ao4c01857_si_001.zip › Figures/XRD-Annealing.pdf]

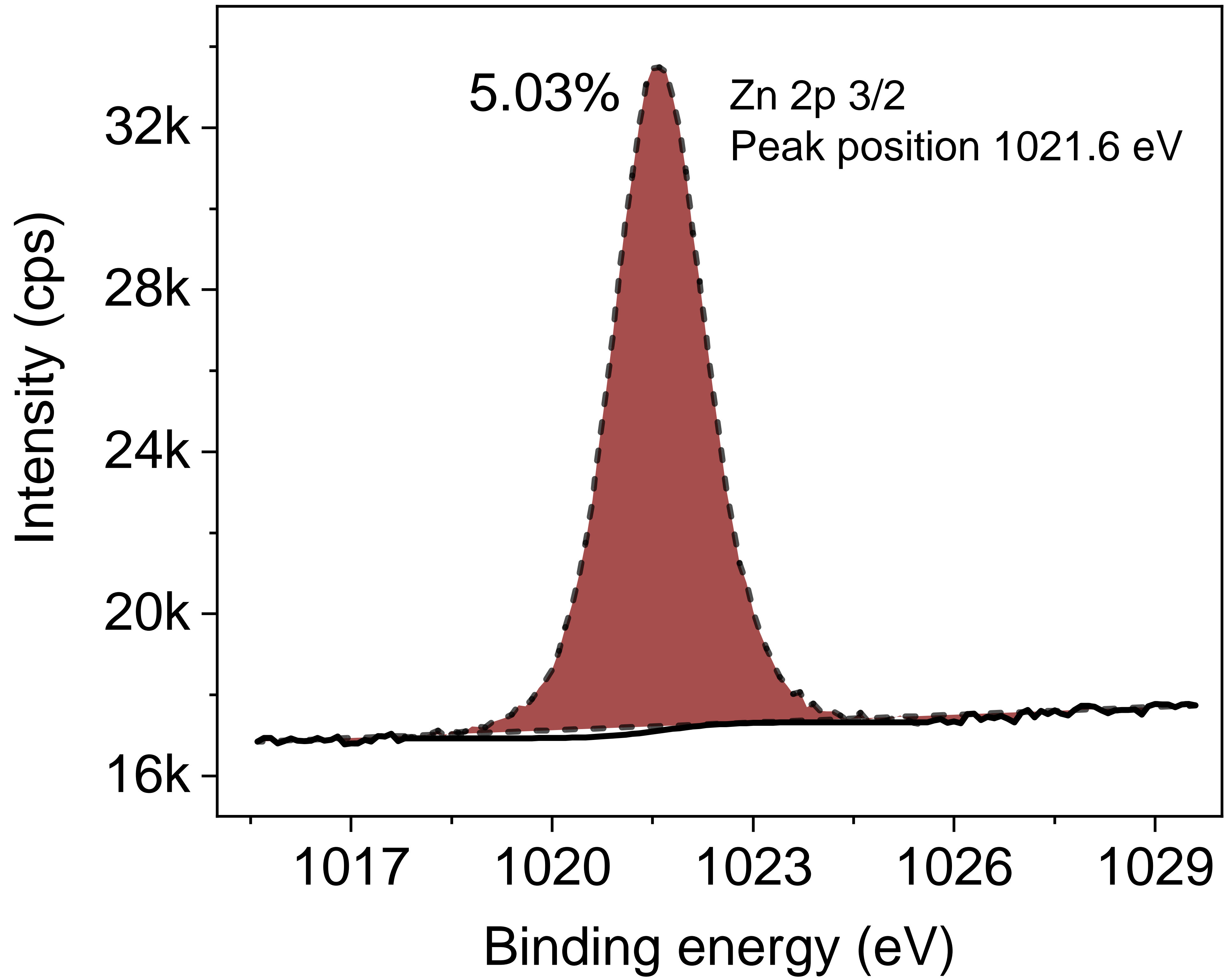

Supplement: Supplementary file 1 — ao4c01857_si_001.zip [file ao4c01857_si_001.zip › Figures/Zinc-XPS-300c.pdf]

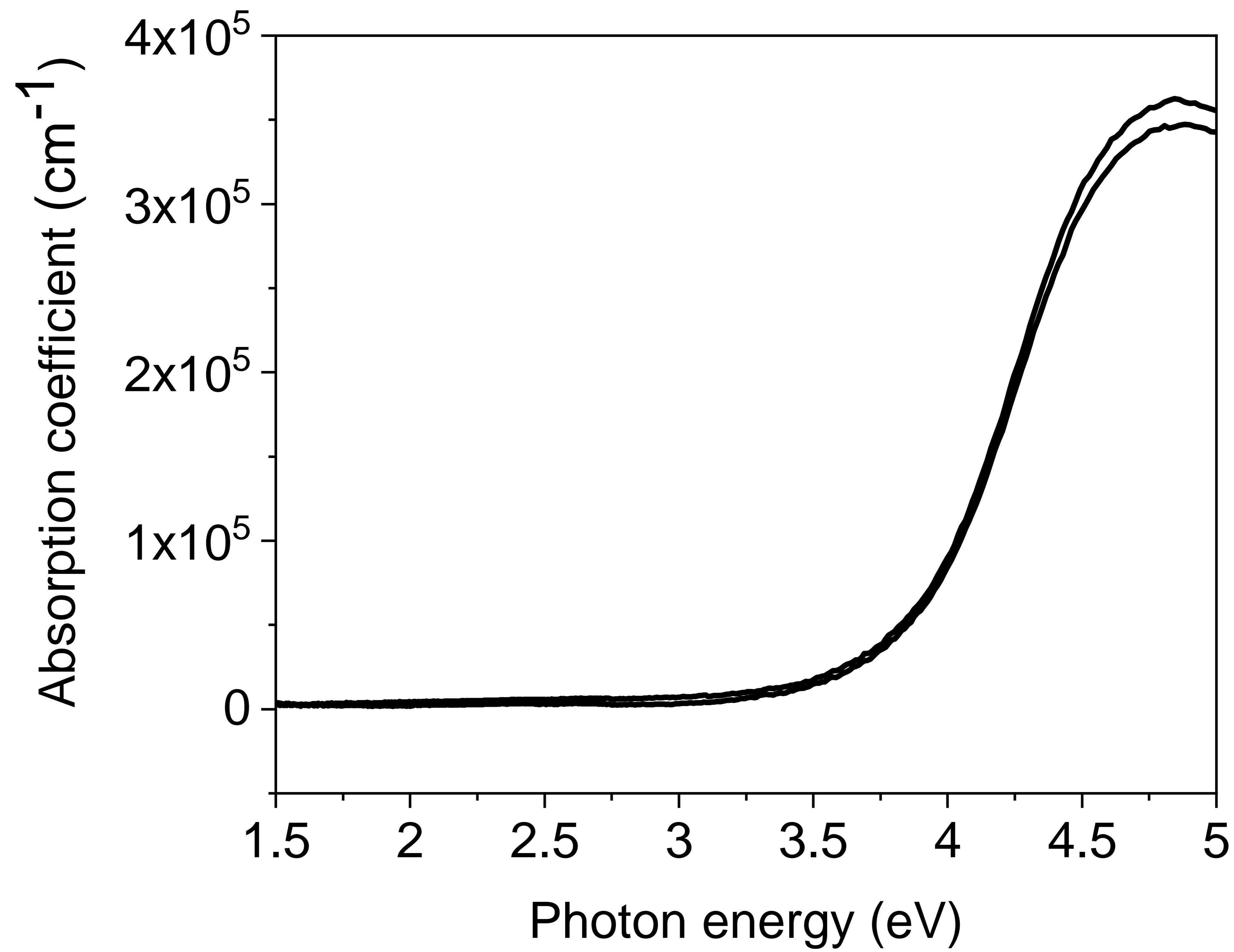

Supplement: Supplementary file 1 — ao4c01857_si_001.zip [file ao4c01857_si_001.zip › Figures/ZTO-ab-coef.pdf]

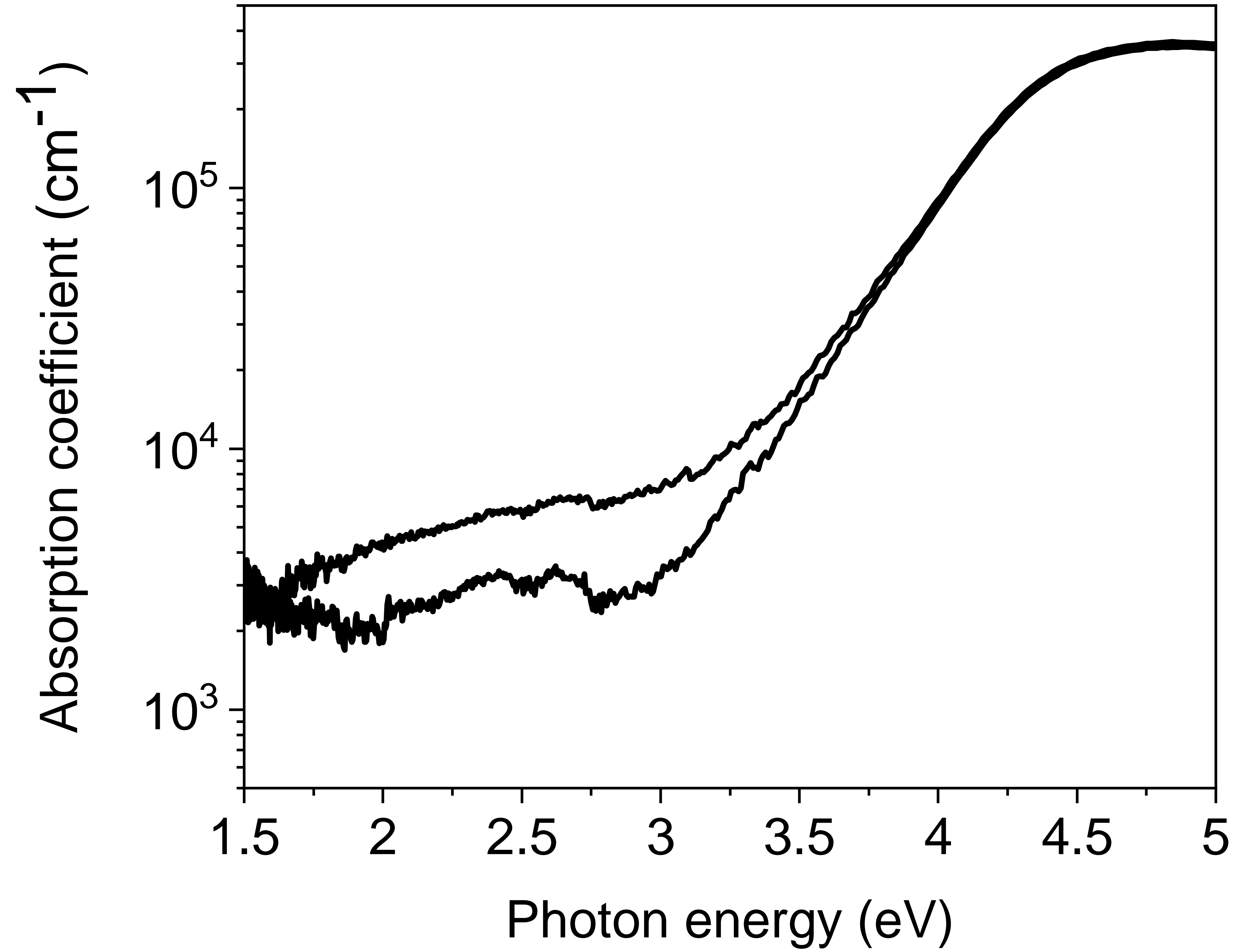

Supplement: Supplementary file 1 — ao4c01857_si_001.zip [file ao4c01857_si_001.zip › Figures/ZTO-ab-log.pdf]

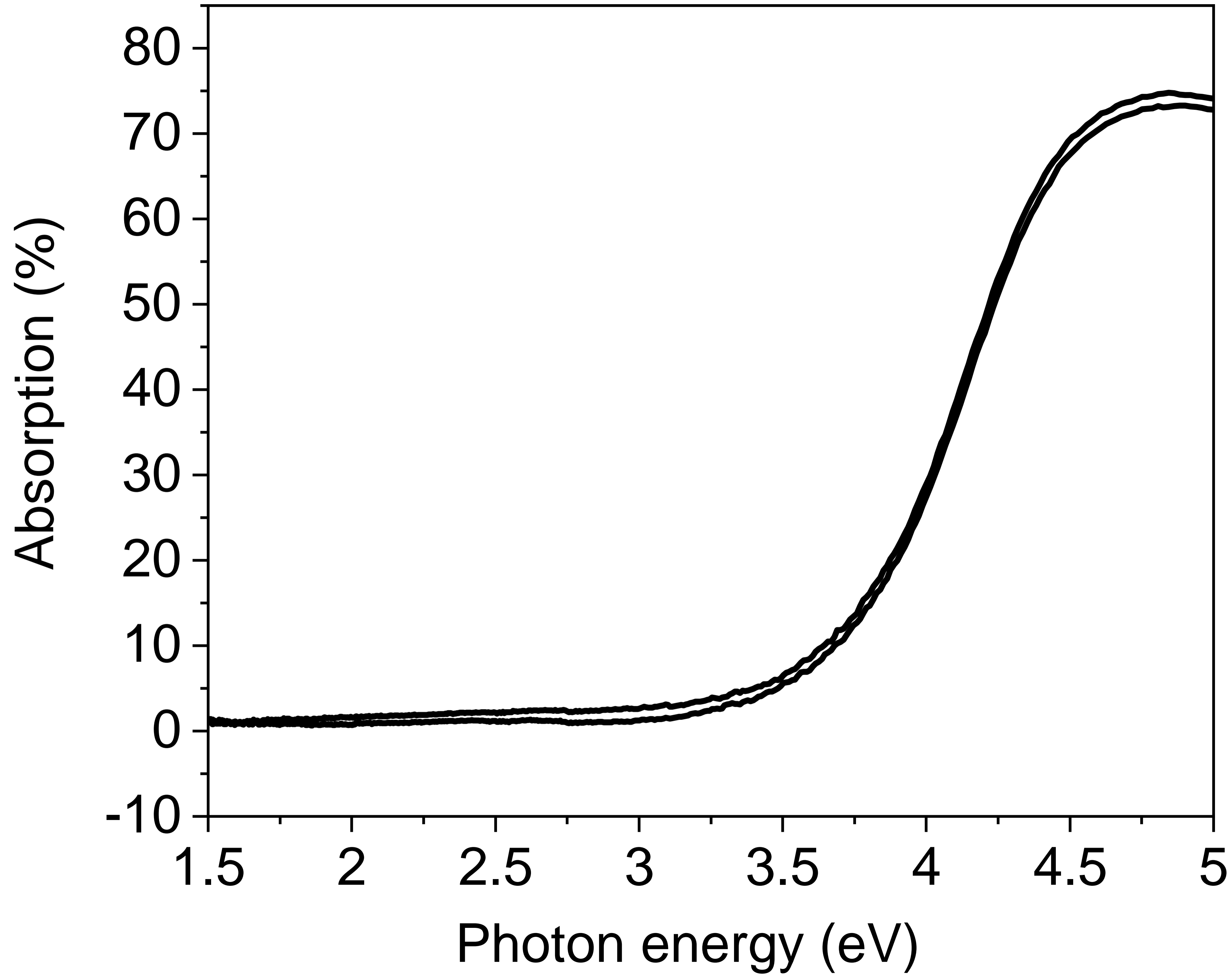

Supplement: Supplementary file 1 — ao4c01857_si_001.zip [file ao4c01857_si_001.zip › Figures/ZTO-ab.pdf]

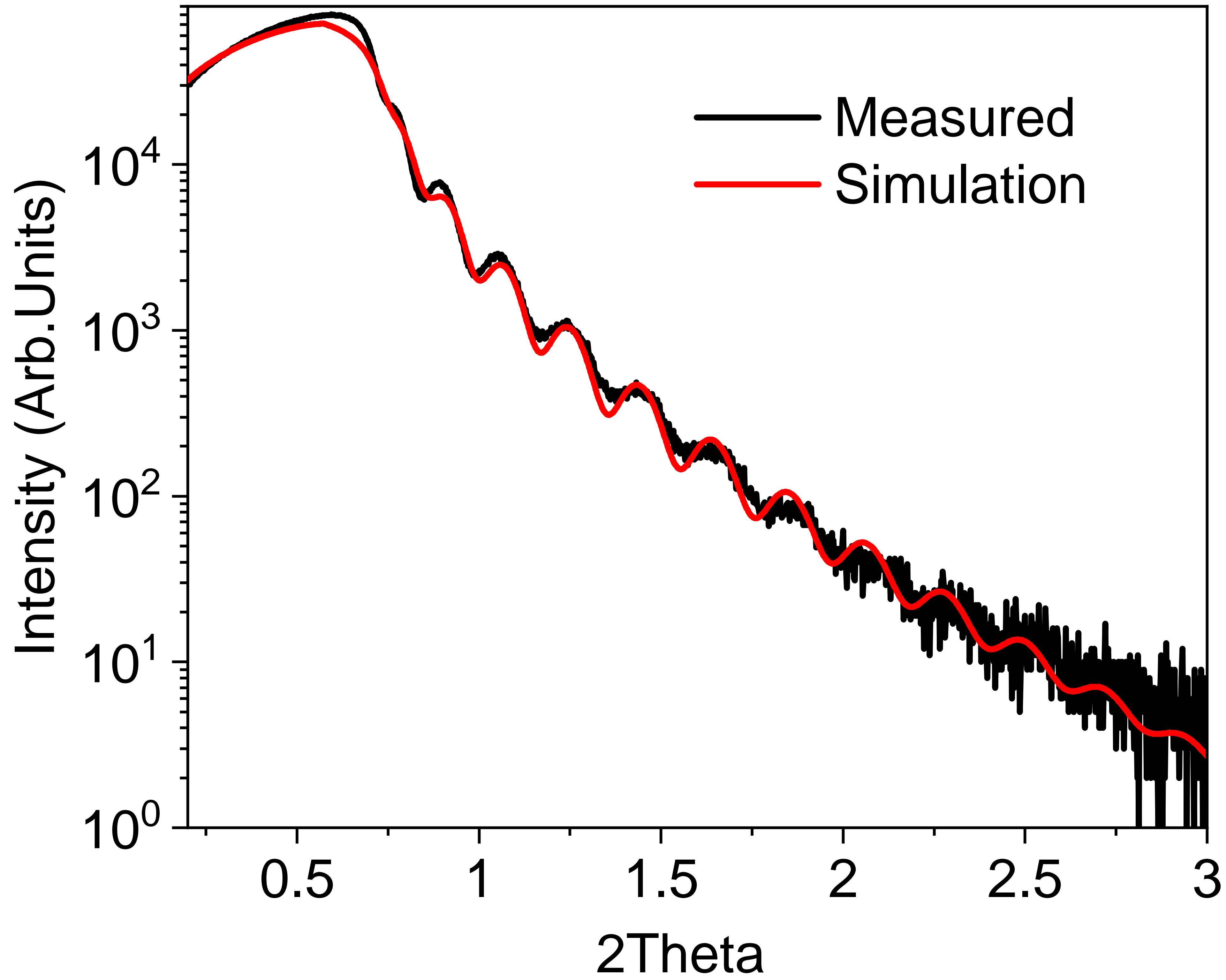

Supplement: Supplementary file 1 — ao4c01857_si_001.zip [file ao4c01857_si_001.zip › Figures/ZTO-annealing-38nm.pdf]

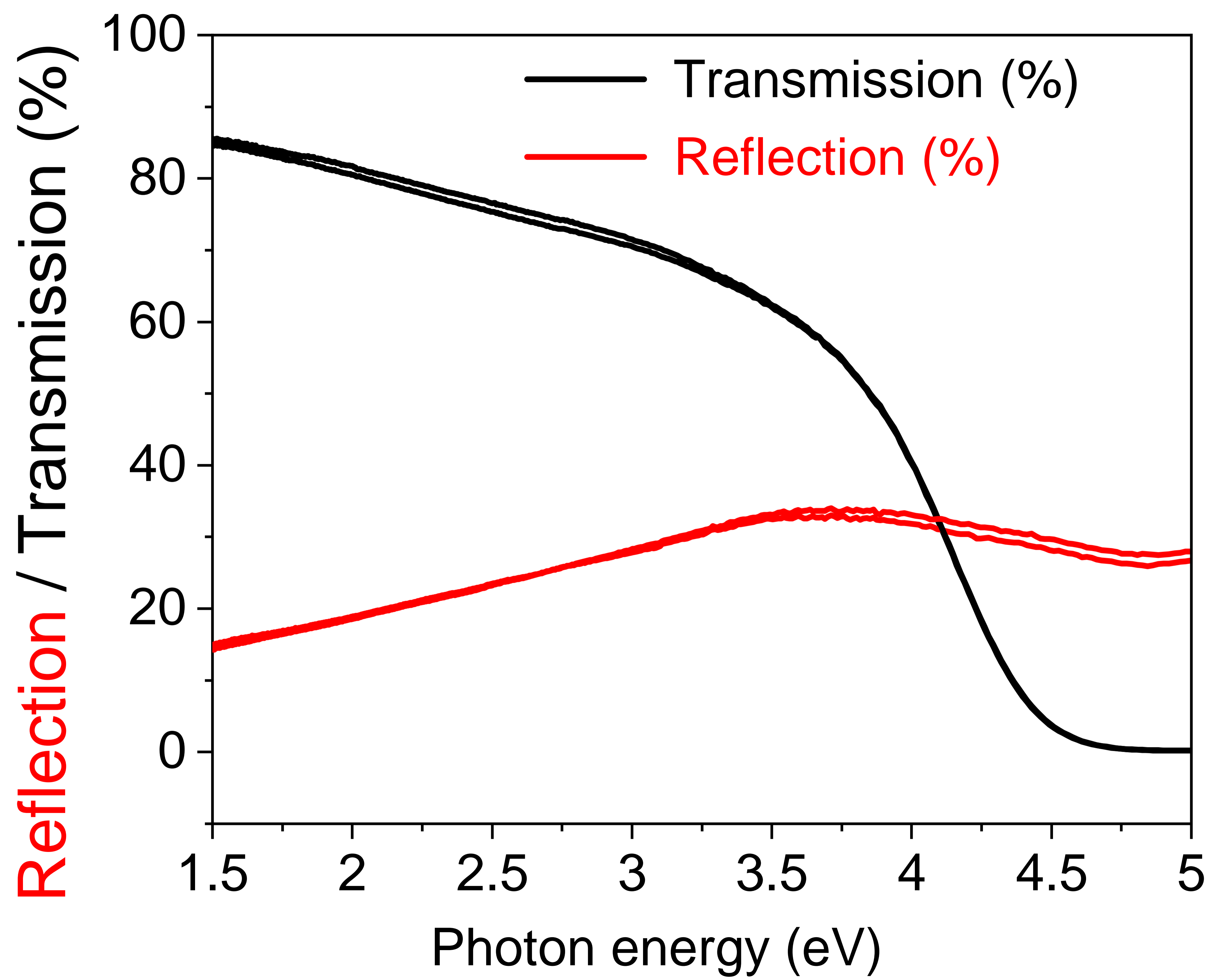

Supplement: Supplementary file 1 — ao4c01857_si_001.zip [file ao4c01857_si_001.zip › Figures/ZTO-TR.pdf]
