# Supplementary material for: Examining the Desirable Properties of ZnSnOy by Annealing Treatment with a Real-Time Observation of Resistivity
Source: ACS Omega. 2024 Jun 5;9(24):26205–12. doi: 10.1021/acsomega.4c01857 (PMC11191124; doi:10.1021/acsomega.4c01857)
Supplement: Supplementary file 2 — ao4c01857_si_002.pdf [file ao4c01857_si_002.pdf]

Examining the Desirable Properties of ZnSnO$_y$ by Annealing Treatment with a Real-Time Observation of Resistivity

Aitkazy Kaisha,[*,†,‡,¶] David Caffrey,[†] Ardak Ainabayev,[†,§] Olzat Toktarbaiuly,[¶] Ibraimov Margulan,[‖] Hongqiang Wang,[⊥] Nurxat Nuraje,[#,¶] and Igor V. Shvets[†]

†*School of Physics and Centre for Research on Adaptive Nanostructures and Nanodevices (CRANN), Trinity College Dublin, Dublin 2, Ireland*

‡*National Nanotechnology Laboratory of Open Type (NNLOT), Al-Farabi Kazakh National University, Al-Farabi Avenue 71, Almaty, 050040, Kazakhstan*

¶*Renewable Energy Laboratory, National Laboratory Astana (NLA), Nazarbayev University, Kabanbay Batyr 53, Astana, 010000, Kazakhstan*

§*Physics Department, School of Sciences and Humanities, Nazarbayev University, Qabanbay Batyr Ave 53, Astana, 010000, Kazakhstan*

‖*Faculty of Physics and Technology, 71 Al-Farabi Ave., Almaty 050040, Kazakhstan*

⊥*State Key Laboratory of Solidification Processing, Center for Nano Energy Materials, School of Materials Science and Engineering, Northwestern Polytechnical University and Shaanxi Joint Laboratory of Graphene (NPU), Xi'an, 710072, P. R. China*

#*Department of Chemical & Materials Engineering, School of Engineering & Digital Science, Nazarbayev University, Astana, 010000, Kazakhstan*

E-mail: kaishaa@tcd.ie

1 XPS data

These a-ZTO films were grown with a precise Zn/Sn ratio in order to indicate the consistency of the annealing studies. The elemental ratio of one of the a-ZTO films is as shown in Figure S1.

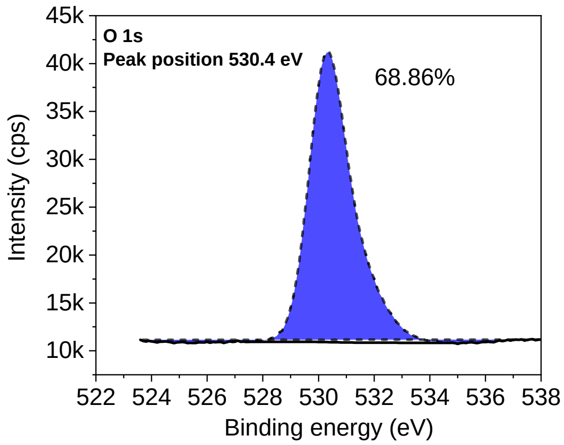

(a)

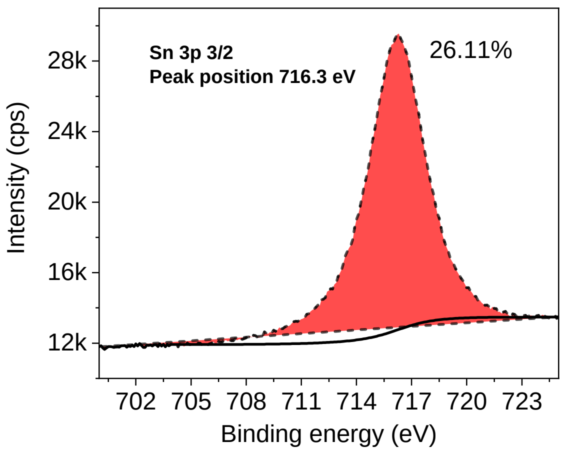

(b)

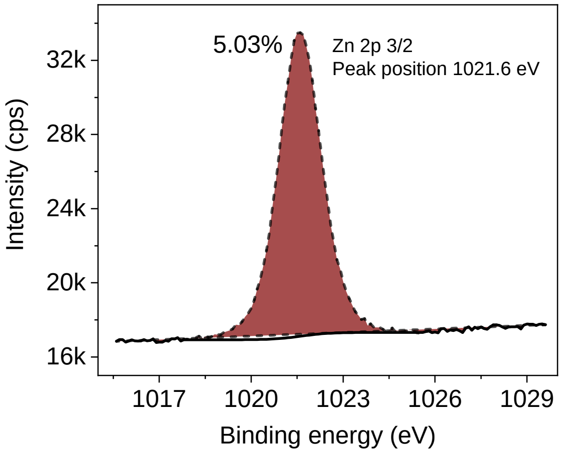

(c)

Figure S1: a-ZTO film deposited at 300 °C and composition study by XPS, where (a) - O XPS data, (b) - Sn XPS data, (c) - Zn XPS data.

2 UV-vis Spectra

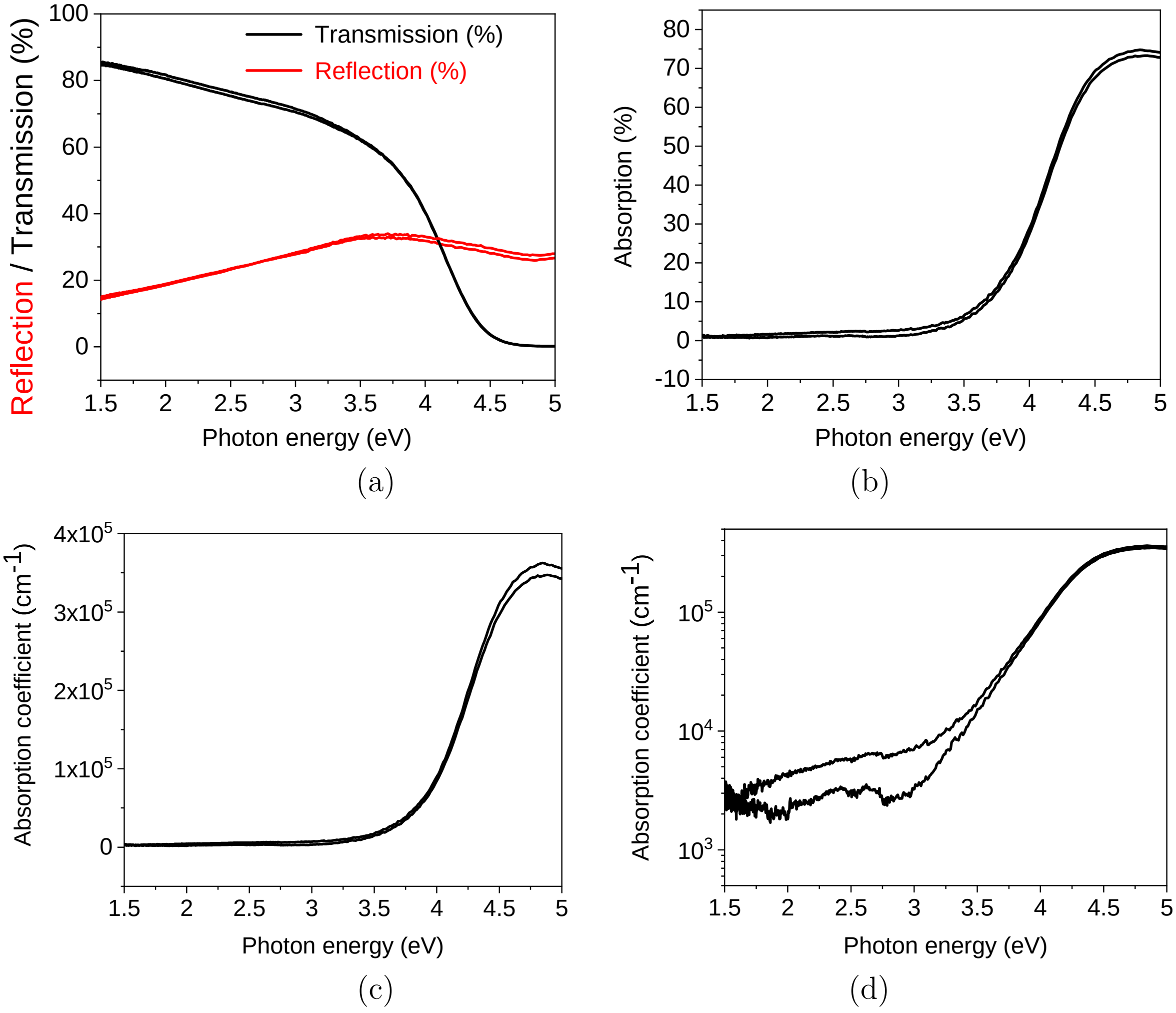

Figure S2: Optical properties of as deposited and annealed a-ZTO films, where (a) - transmission (black line) and reflection spectra (red line), (b) - absorption, (c) - absorption coefficient (linear), (d) - absorption coefficient (log) of a-ZTO films.

Figure S2 shows the transmission and reflectance spectra, absorption and absorption coefficient (linear) and absorption coefficient (log) of as-deposited and annealed a-ZTO films. It is clear that the transmission is around 80% ($\approx$ 18% of reflection) for films within the visible spectra region of light. No variation in transmission and reflectance spectra of films. However, 1-2% range noise level in our system results in a small alteration in absorption coefficients as shown in Figure S2 (d). All imply that a-ZTO films in this work show high optical transparency within the visible light spectrum range. Transparency is an essential

factor in fulfilling the high transparency requirement of some optoelectronic devices, such as TFTs.[1]

3 a-ZTO bandgap

Bandgap of 38 nm thick a-ZTO film on Glass substrate is also shown in Figure S3.

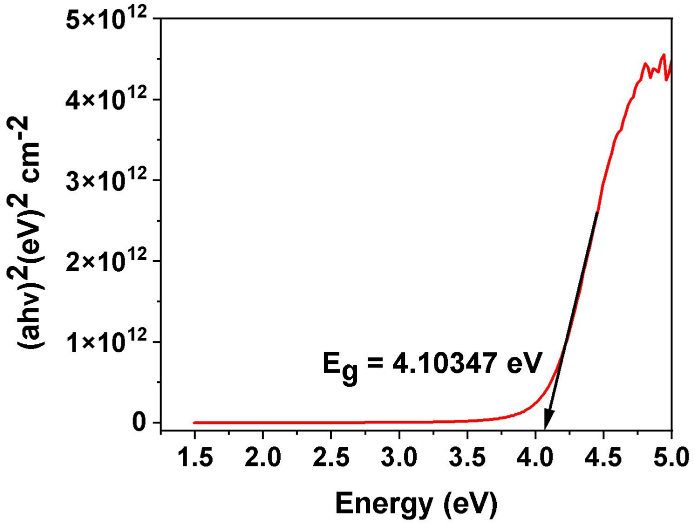

Figure S3: Bandgap of 38 nm thick a-ZTO film deposited at 300 °C on Glass substrate.

4 SEM data

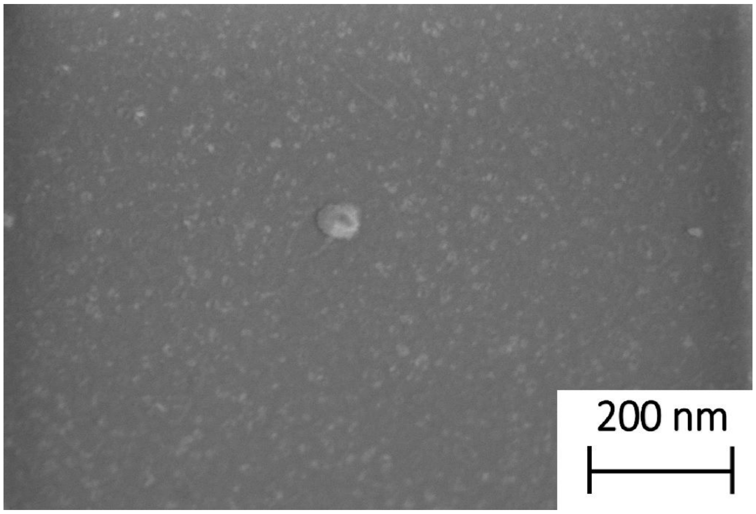

Figure S4: SEM image of 38 nm thick a-ZTO film deposited at 300 °C on Glass substrate.

a-ZTO film deposited at 300 °C and the surface morphology of it was examined by Scanning Electron Microscopy (SEM) as shown in Figure S4. SEM image confirms that there are no discontinuities and deterioration on the surface of a-ZTO film. This means that smooth surface of a-ZTO film was obtained by magnetron sputtering. The cross-section SEM images of a-ZTO films can be foun in our previous publication.[2]

5 XRR and XRD data

The thickness of the films was measured by the high-resolution X-ray Diffractometer. X-ray Diffraction (XRD) confirmed the amorphous nature of a-ZTO films.

Figure S5 (a) shows XRR data of 38 nm thick a-ZTO film. Fittings were done via the Bruker Leptos software program, where fitting matches very well between simulation and measured data of the sample. This also highlights that XRR data give thickness, roughness and density of the materials in this work. The average density of the 6.76 g/cm^3 and roughness of ≈ 0.42 nm for a-ZTO was obtained.

Fig S5 (b) shows a typical XRD pattern of the a-ZTO films. It is clear that three halo

peaks were observed at around 13°, 28°, and 42° due to the presence of amorphous bonds, which vary in length within the material. It is usually called a halo peak and is an indicator of an amorphous structure. The former two peaks originate from the thin film and part of the glass. The peak at 13° is from an amorphous structure. The glass peak is around 20-30°, and this halo peak is consistent with the previous report.[3] The peak at 40-45° is from the XRD sample holder. It can be seen from Figure S5 that sharp peaks were not observed by XRD. All a-ZTO samples in this work showed an amorphous structure.

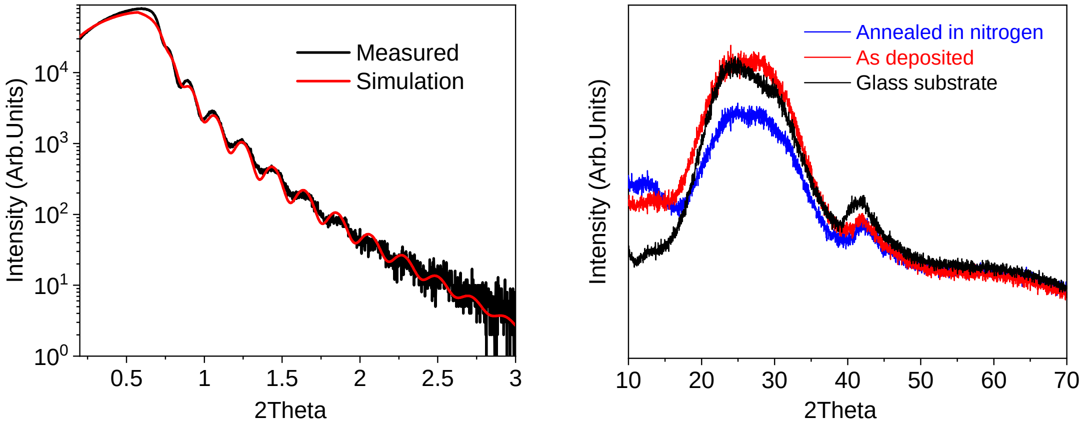

(a) (b)

Figure S5: XRR and XRD pattern of a-ZTO deposited on SiO_2 glass substrate by Magnetron sputtering, where (a) - XRR pattern of 38 nm thick a-ZTO, (b) - XRD pattern of glass, a-ZTO as deposited and annealed in nitrogen atmosphere.

6 Annealing graphs

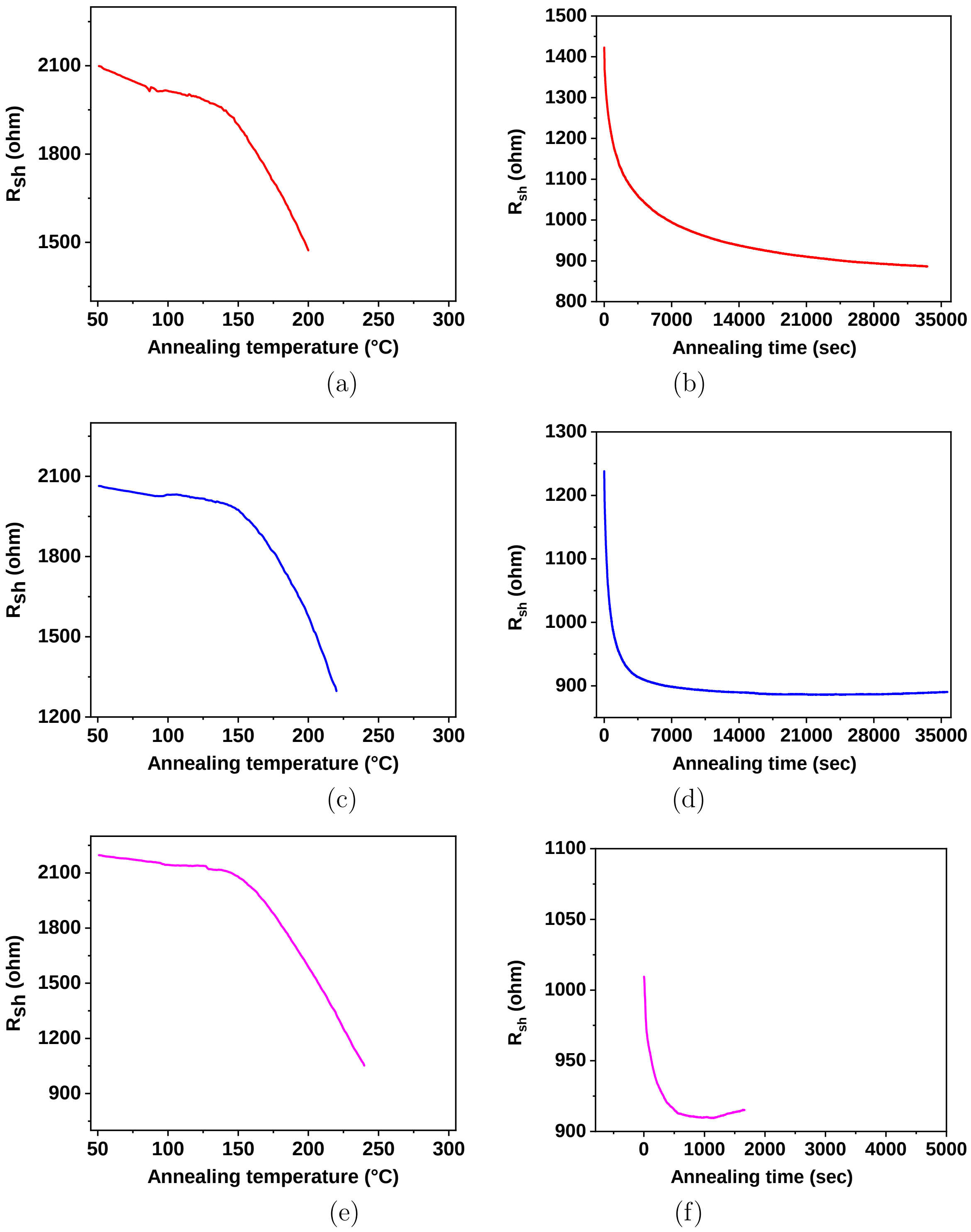

Figure S6: a-ZTO film annealed at different temperatures, where (a) and (b) - annealing temperature 200 °C, (c) and (d) - annealing temperature 220 °C, (e) and (f) - annealing temperature 240 °C.

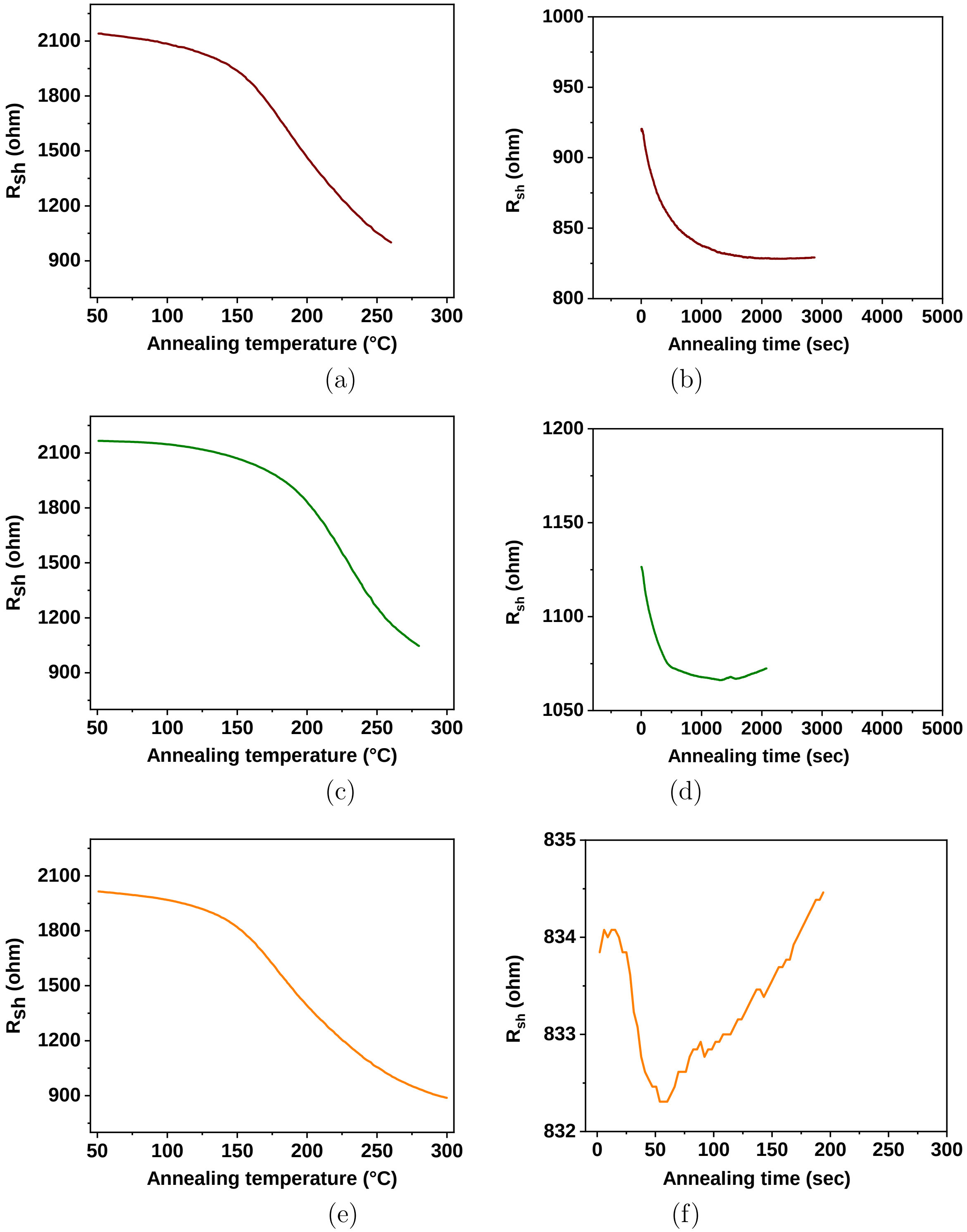

Figure S7: a-ZTO film annealed at different temperatures, where (a) and (b) - annealing temperature of 260 °C, (c) and (d) - annealing temperature of 280 °C, (e) and (f) - annealing temperature of 300 °C.

References

(1) Kumar, N.; Joshi, B.; Asokan, K. The effects of thermal annealing on the structural and electrical properties of zinc tin oxide thin films for transparent conducting electrode applications. *Physica B: Condensed Matter* **2019**, *558*, 5–9.

(2) Zhussupbekova, A.; Kaisha, A.; Vijayaraghavan, R. K.; Fleischer, K.; Shvets, I. V.; Caffrey, D. Importance of Local Bond Order to Conduction in Amorphous, Transparent, Conducting Oxides: The Case of Amorphous ZnSnOy. *ACS Applied Materials and Interfaces* **2019**, *11*, 44399–44405.

(3) Jiang, Q. J.; Wu, C. J.; Cheng, J. P.; Li, X. D.; Lu, B.; Ye, Z. Z.; Lu, J. G. Solvent sensors based on amorphous ZnSnO thin-film transistors. *RSC Advances* **2015**, *5*, 28242–28246.
